# Supplementary material for: Maintenance of chronic neuroinflammation in multiple sclerosis via interferon signaling and CD8 T cell-mediated cytotoxicity
Source: bioRxiv. 2025 Jun 11:2025.06.09.658729. Preprint. [Version 1] doi: 10.1101/2025.06.09.658729 (PMC12190403; doi:10.1101/2025.06.09.658729)

M11 greenyellow module

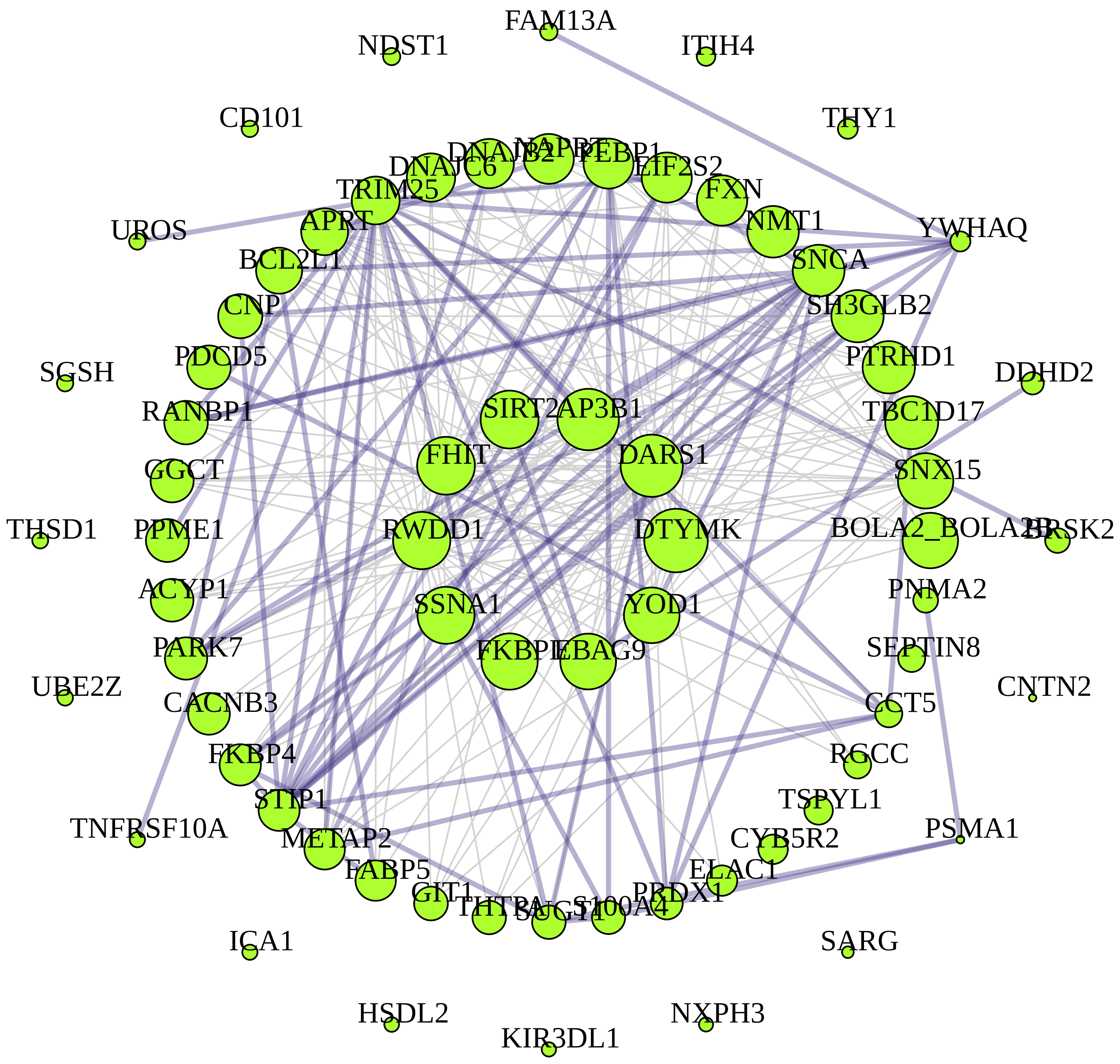

M11 greenyellow module hubs connected by top 374 TOM edges: HUB^{degree}

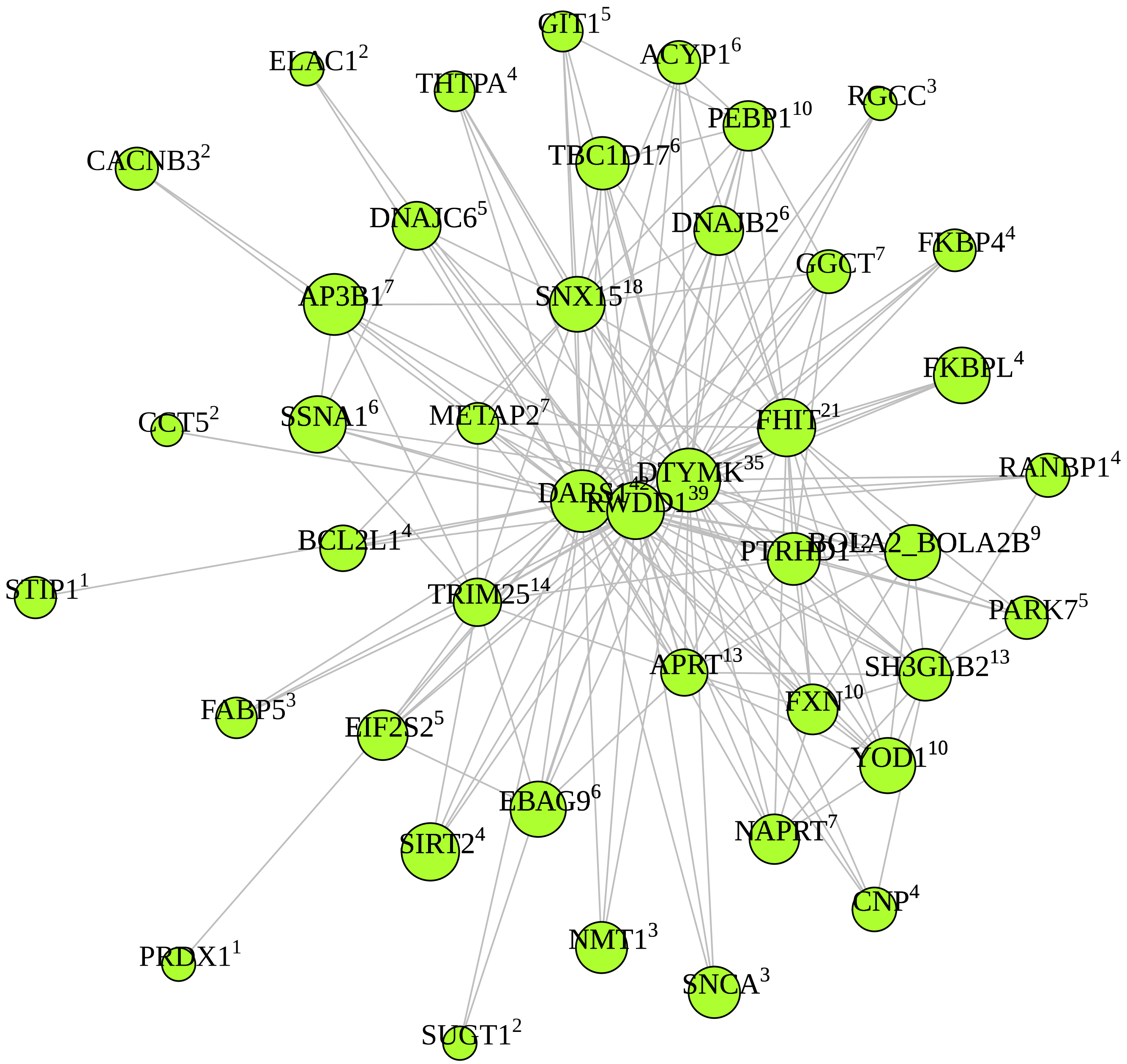

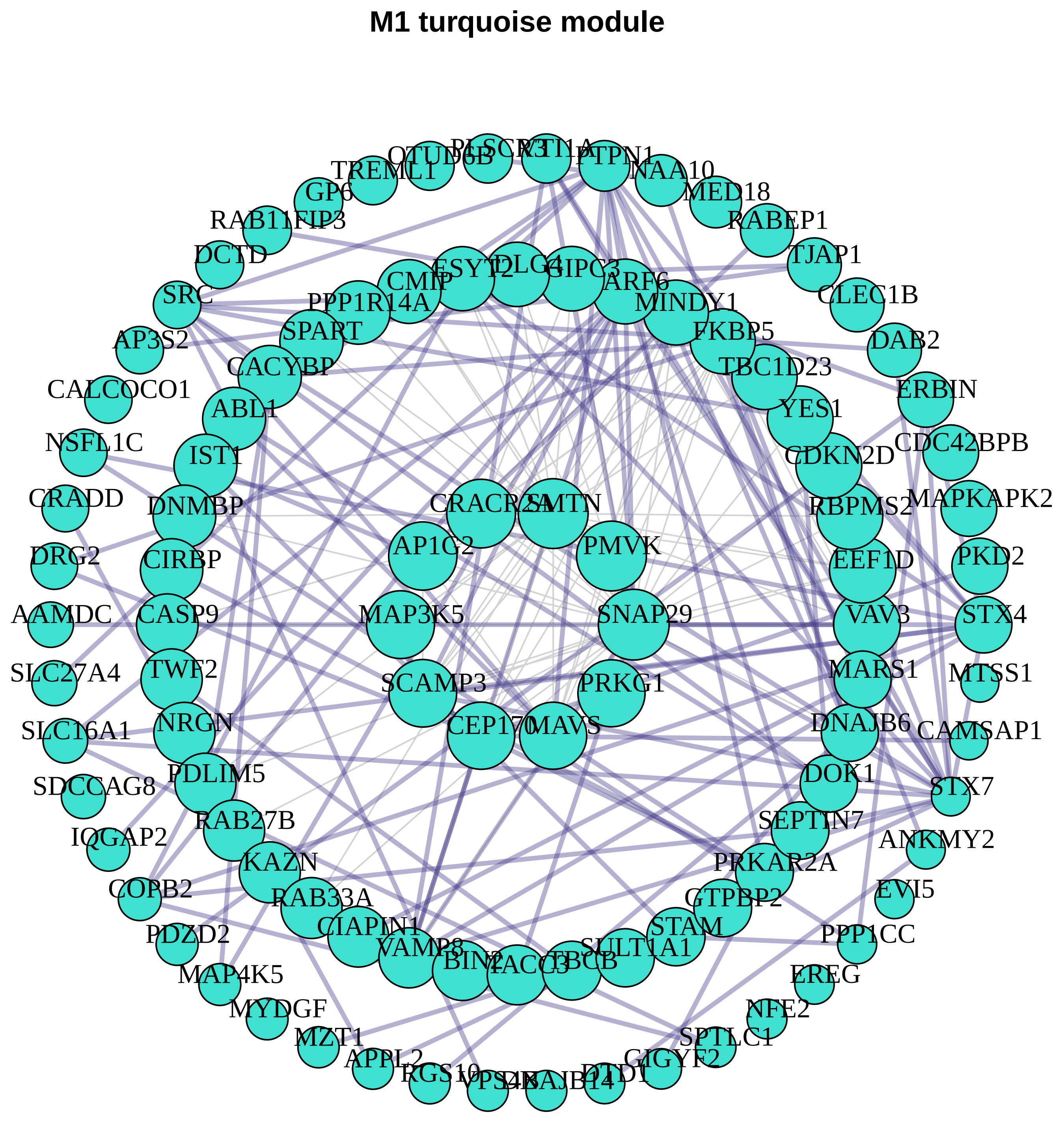
M1 turquoise module
PTPN1
NAA10
MED18
RABEP1
TJAP1
CLEC1B
DAB2
ERBIN
CDC42BPB
MAPKAPK2
PKD2
STX4
MTSS1
CAMSAP1
STX7
ANKMY2
EVI5
PPP1CC
EREG
NFE2
SPTLC1
GIGYF2
APPL2
MZT1
MYDGF
MAP4K5
PDZD2
COPB2
IQGAP2
SDCCAG8
SLC16A1
SLC27A4
AAMDC
DRG2
CRADD
NSFL1C
CALCOCO1
AP3S2
SRC
DCTD
RAB11FIP3
GP6
TREML1
CMIP
ESYT2
GIPC3
ARF6
MINDY1
FKBP5
TBC1D23
YES1
CDKN2D
RBPMS2
EEF1D
VAV3
MARS1
DNAJB6
DOK1
SEPTIN7
PRKAR2A
GTPBP2
STAM
SULT1A1
TBCB
BIN2
VAMP8
CIAPIN1
RAB33A
KAZN
RAB27B
PDLIM5
NRGN
TWF2
CASP9
CIRBP
DNMBP
IST1
ABL1
CACYBP
SPART
PPP1R14A
AP1G2
MAP3K5
SCAMP3
CEP170
MAVS
PRKG1
SNAP29
PMVK
SMTN

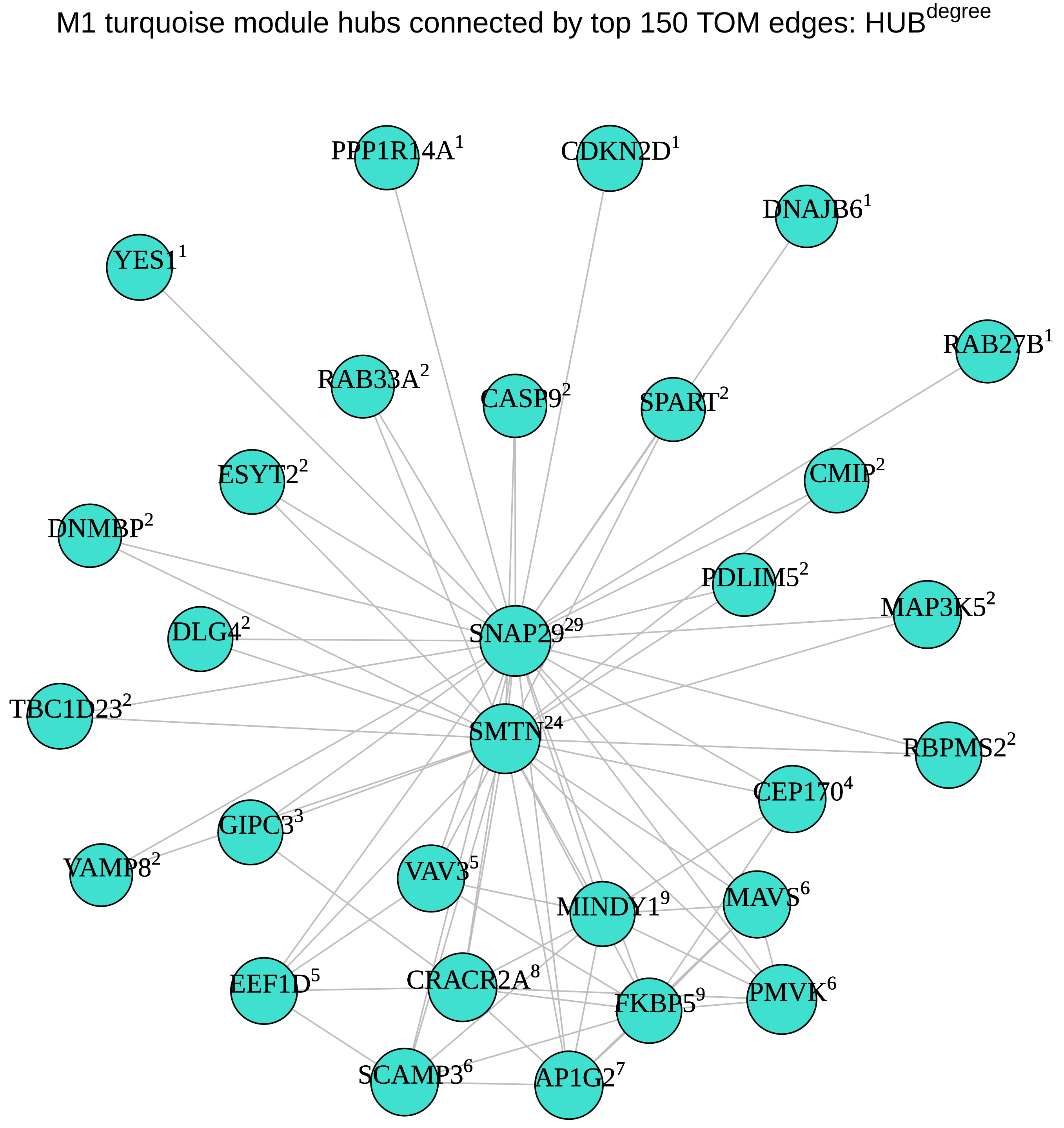
M1 turquoise module hubs connected by top 150 TOM edges: HUB^{degree}
PPP1R14A^{1}
CDKN2D^{1}
DNAJB6^{1}
YES1^{1}
RAB27B^{1}
RAB33A^{2}
CASP9^{2}
SPART^{2}
ESYT2^{2}
CMIP^{2}
DNMBP^{2}
PDLIM5^{2}
MAP3K5^{2}
DLG4^{2}
SNAP29^{29}
TBC1D23^{2}
SMTN^{24}
RBPMS2^{2}
CEP170^{4}
GIPC3^{3}
VAMP8^{2}
VAV3^{5}
MINDY1^{9}
MAVS^{6}
EEF1D^{5}
CRACR2A^{8}
FKBP5^{9}
PMVK^{6}
SCAMP3^{6}
AP1G2^{7}

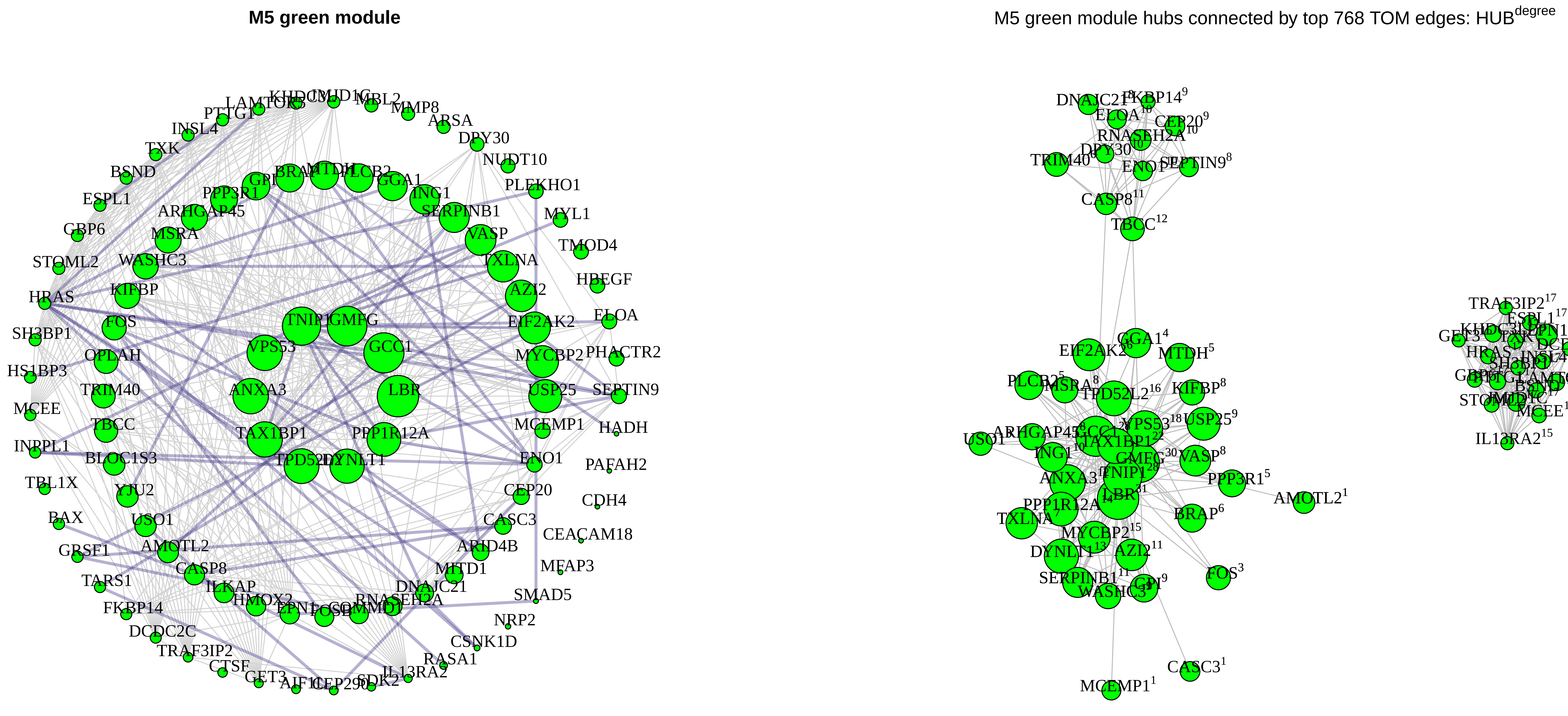
M5 green module
M5 green module hubs connected by top 768 TOM edges: HUB^degree
MBL2
MMP8
ARSA
DPY30
NUDT10
PLEKHO1
MYL1
TMOD4
HBEGF
ELOA
PHACTR2
SEPTIN9
HADH
PAFAH2
CDH4
CEACAM18
MFAP3
SMAD5
NRP2
CSNK1D
RASA1
IL13RA2
SDK2
CTSF
GET3
TRAF3IP2
DCDC2C
FKBP14
TARS1
GRSF1
BAX
TBL1X
INPPL1
MCEE
HS1BP3
SH3BP1
HRAS
STOML2
GBP6
ESPL1
BSND
TXK
INSL4
PTTG1
TNIP1
GMFG
VPS53
GCC1
ANXA3
LBR
TAX1BP1
PPP1R12A
DYNLT1
MYCBP2
USP25
EIF2AK2
AZI2
TXLNA
VASP
SERPINB1
ING1
GGA1
PLCB2
MTDH
ARHGAP45
MSRA
WASHC3
KIFBP
FOS
OPLAH
TRIM40
TBCC
BLOC1S3
YJU2
USO1
AMOTL2
CASP8
ILKAP
HMOX2
EPN1
COMMD1
DNAJC21
MITD1
ARID4B
CASC3
CEP20
ENO1
MCEMP1
MCEMP1^1
CASC3^1
AMOTL2^1
FOS^3
BRAP^6
PPP3R1^5
TBCC^12
CASP8^11
IL13RA2^15
MCEE^17
TRAF3IP2^17

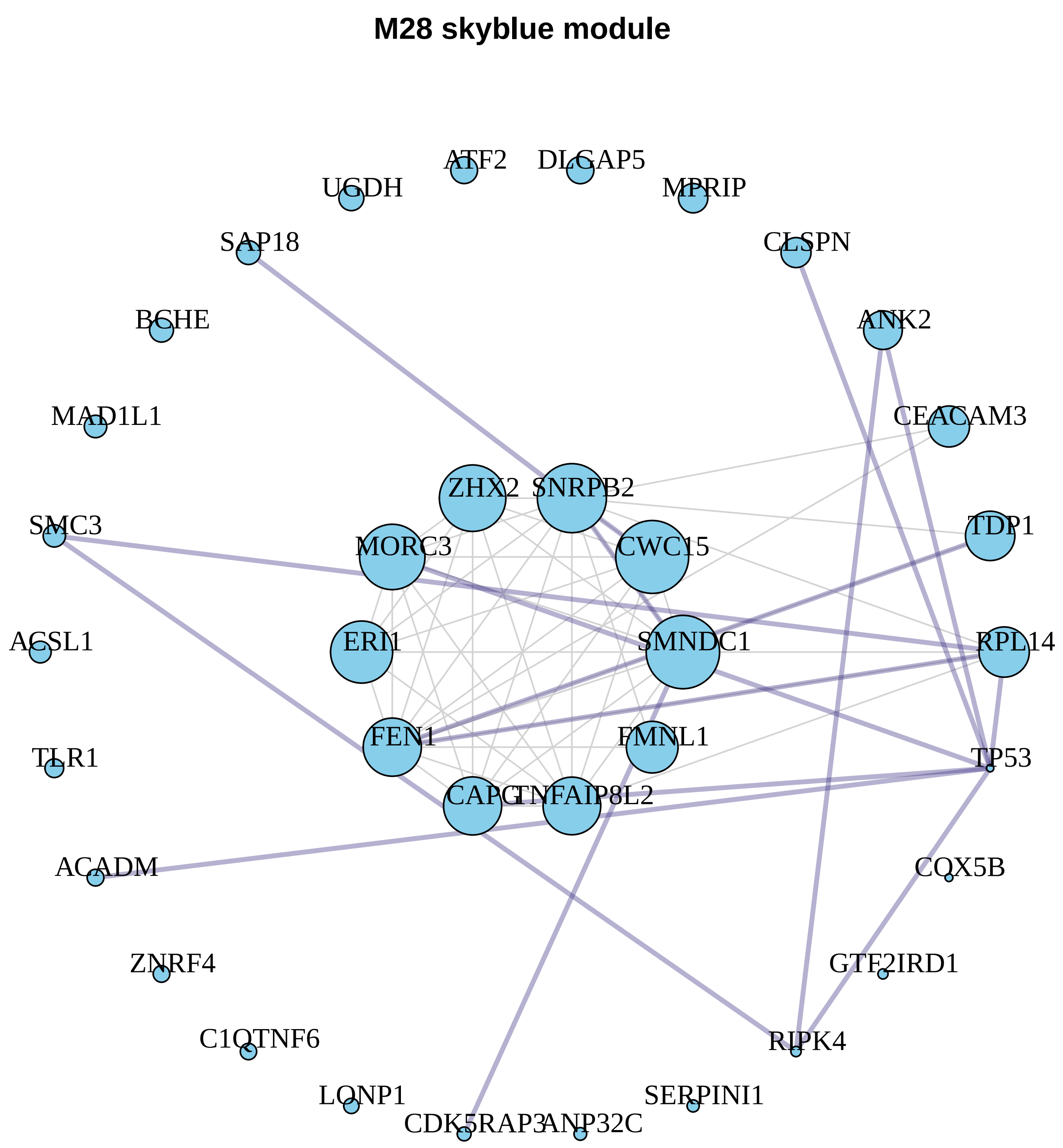
M28 skyblue module
ATF2
DLGAP5
UGDH
MPRIP
SAP18
CLSPN
BCHE
ANK2
MAD1L1
CEACAM3
ZHX2
SNRPB2
SMC3
TDP1
MORC3
CWC15
ACSL1
ERI1
SMNDC1
RPL14
TLR1
FEN1
FMNL1
TP53
CAPG
TNFAIP8L2
ACADM
COX5B
ZNRF4
GTF2IRD1
C1QTNF6
RIPK4
LONP1
SERPINI1
CDK5RAP3
ANP32C

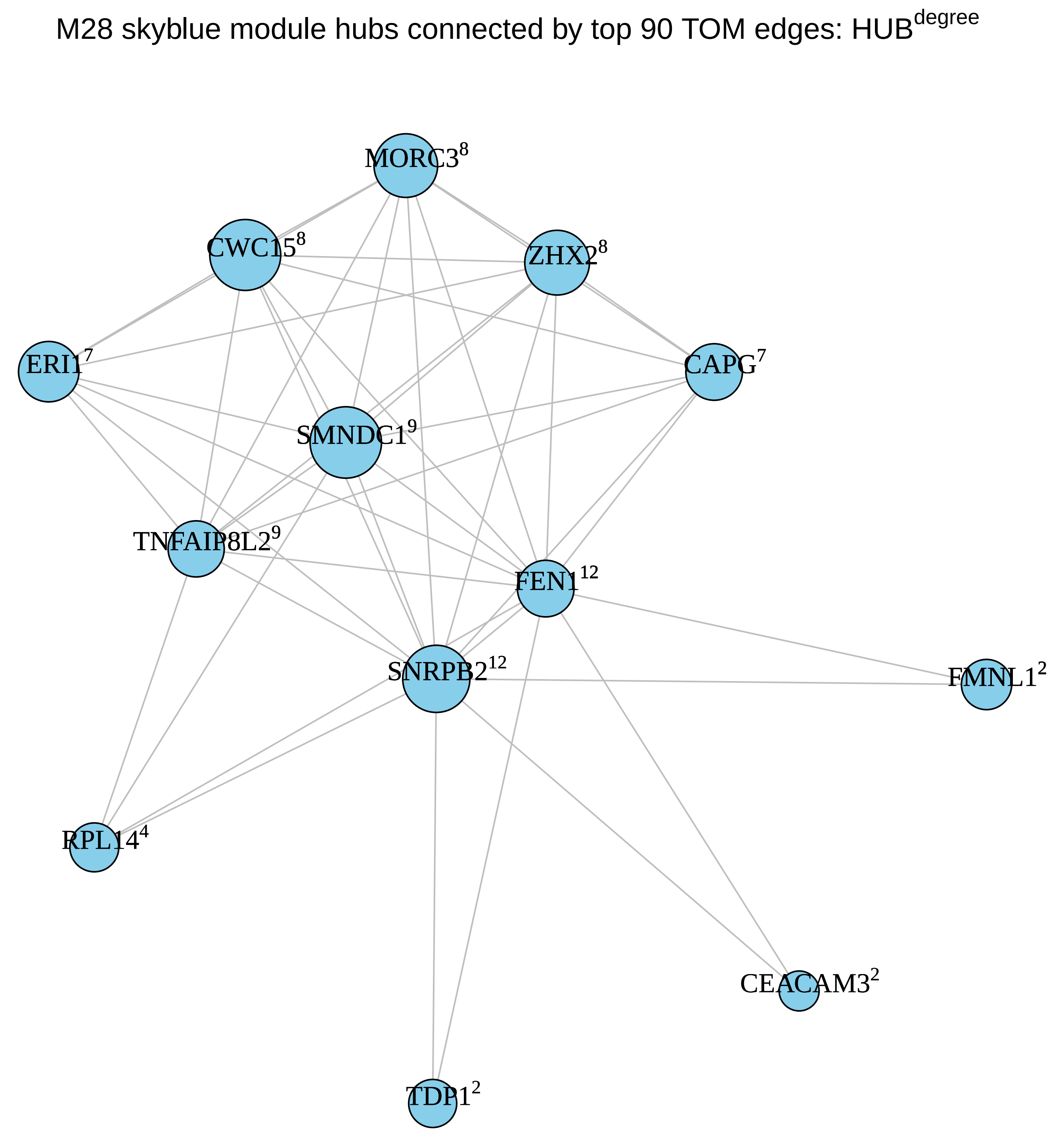
M28 skyblue module hubs connected by top 90 TOM edges: HUB degree
MORC3 8
CWC15 8
ZHX2 8
ERI1 7
CAPG 7
SMNDC1 9
TNFAIP8L2 9
FEN1 12
SNRPB2 12
FMNL1 2
RPL14 4
CEACAM3 2
TDP1 2

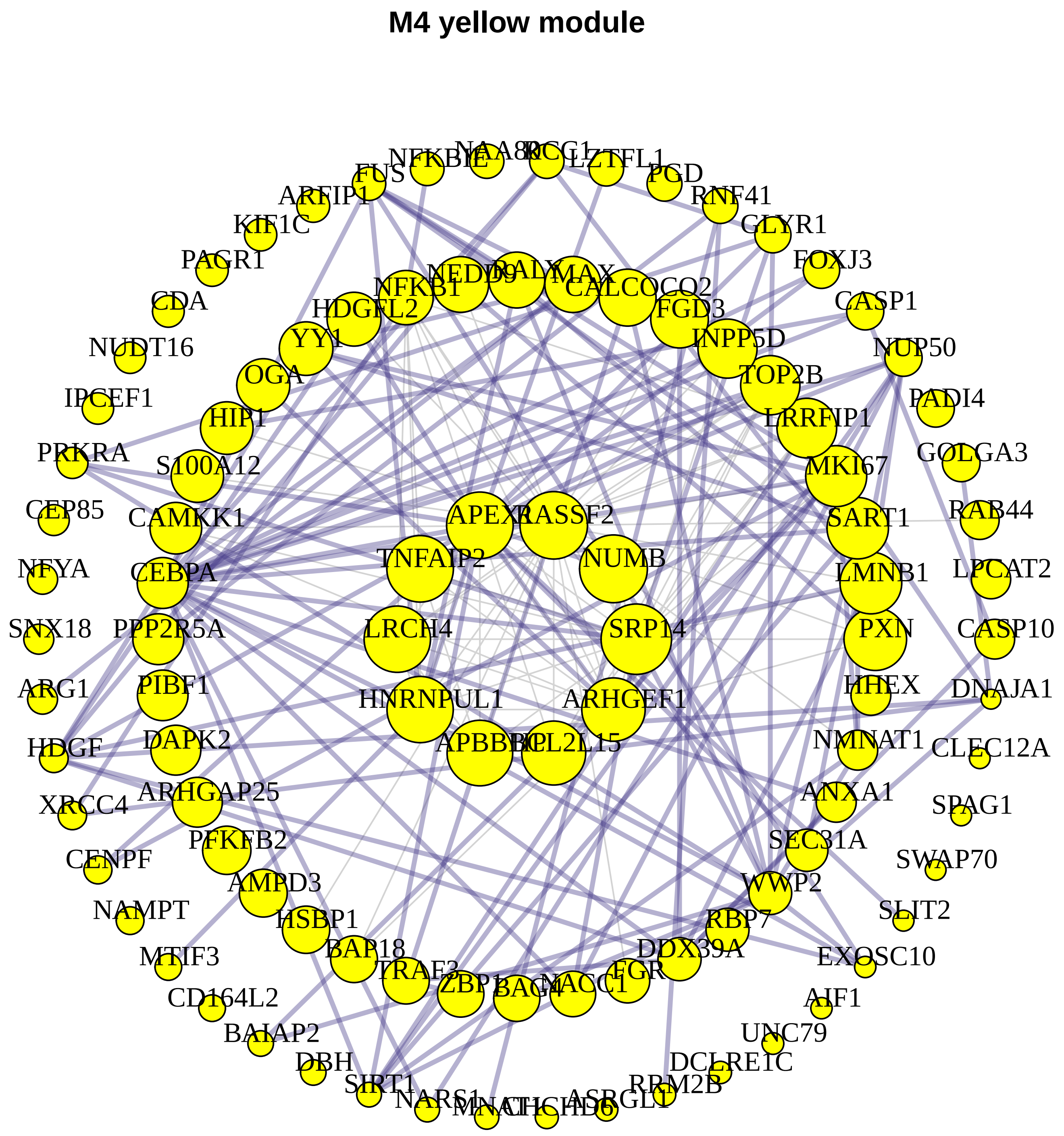
M4 yellow module

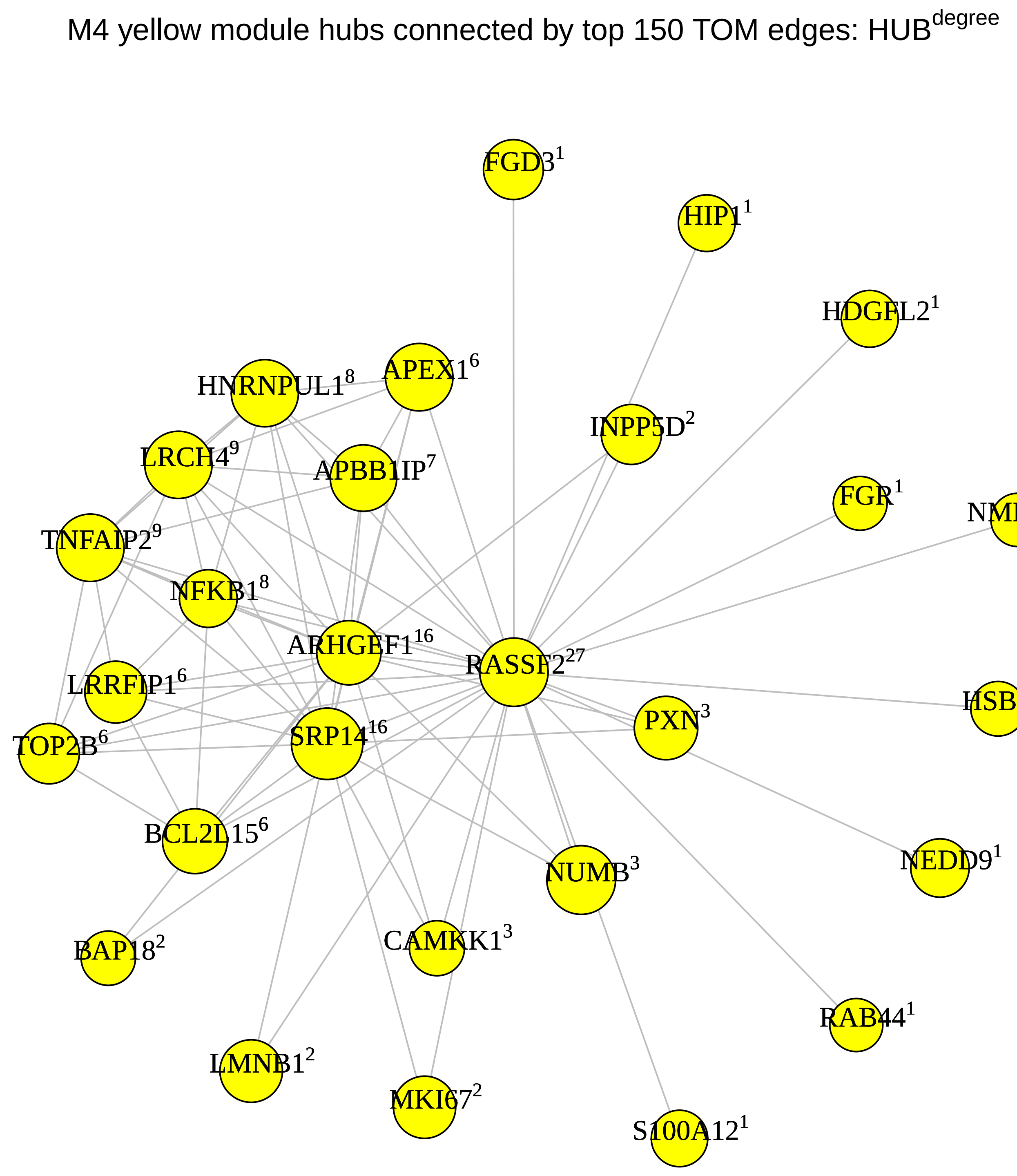
M4 yellow module hubs connected by top 150 TOM edges: HUB^degree
FGD3 1
HIP1 1
HDGFL2 1
HNRNPUL1 8
APEX1 6
INPP5D 2
LRCH4 9
APBB1IP 7
FGR 1
NMNAT1 1
TNFAIP2 9
NFKB1 8
ARHGEF1 16
RASSF2 27
LRRFIP1 6
HSBP1 1
PXN 3
TOP2B 6
SRP14 16
BCL2L15 6
NUMB 3
NEDD9 1
BAP18 2
CAMKK1 3
RAB44 1
LMNB1 2
MKI67 2
S100A12 1

M26 darkorange module

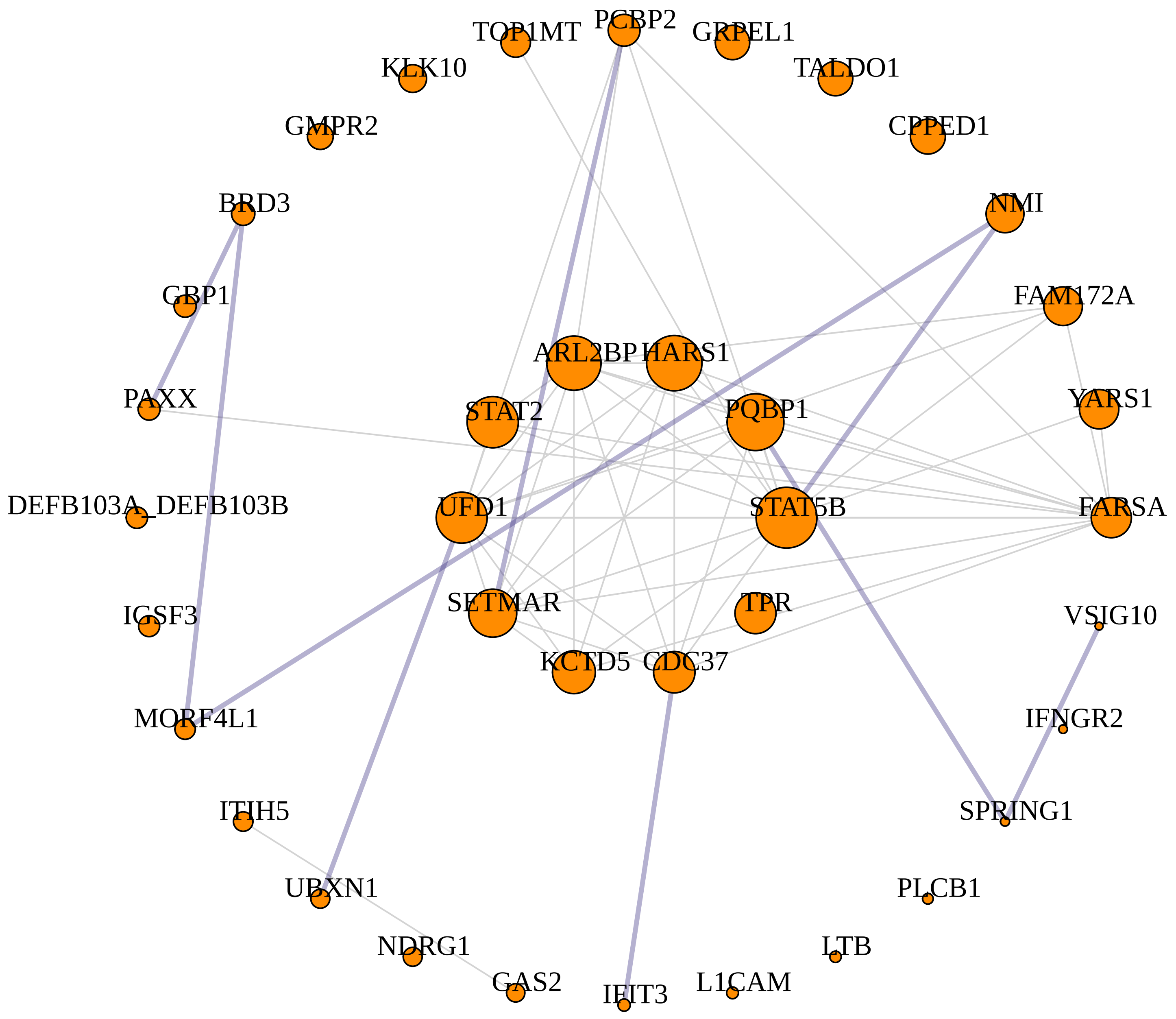

M26 darkorange module hubs connected by top 102 TOM edges: HUB^{degree}

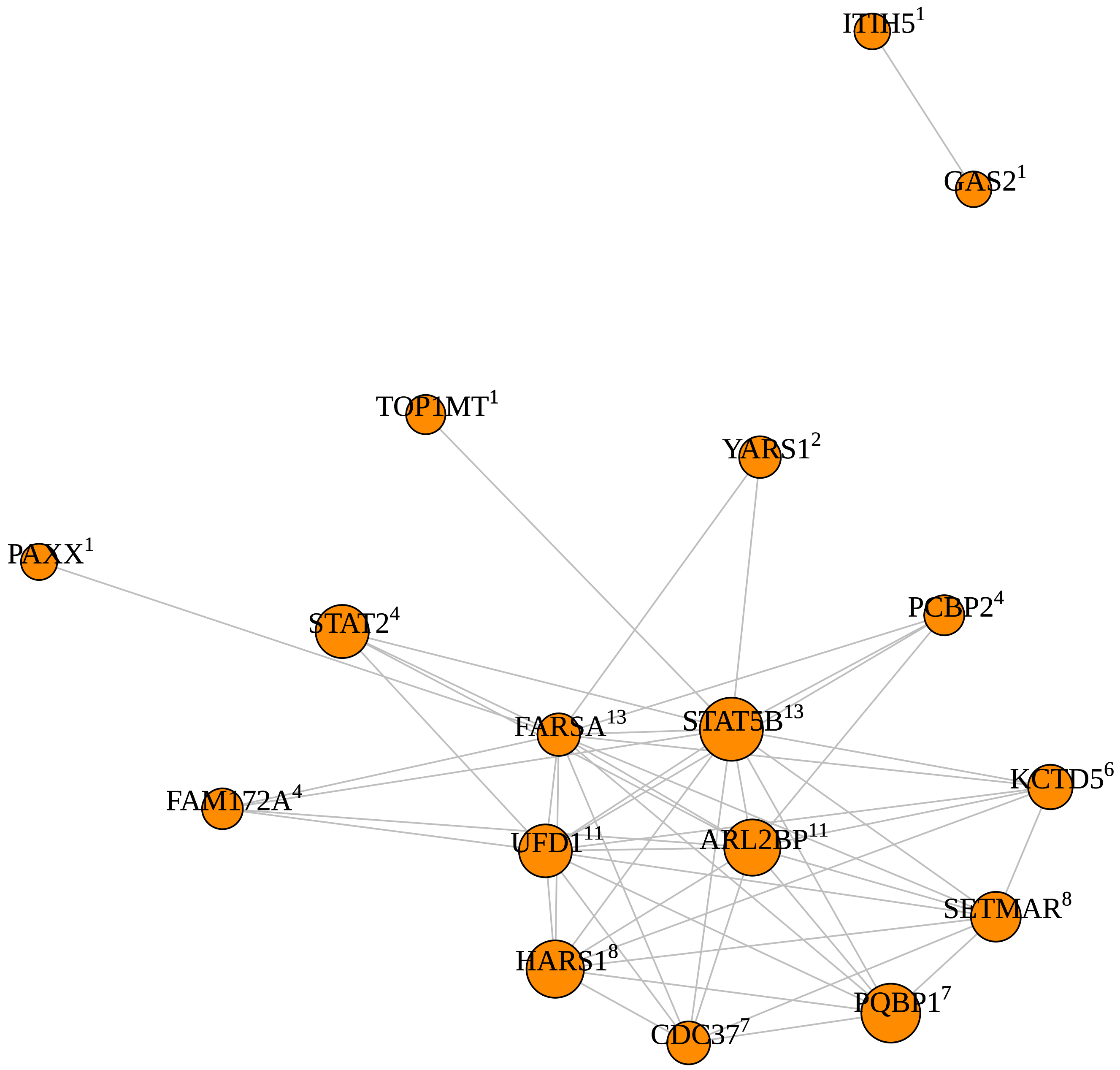

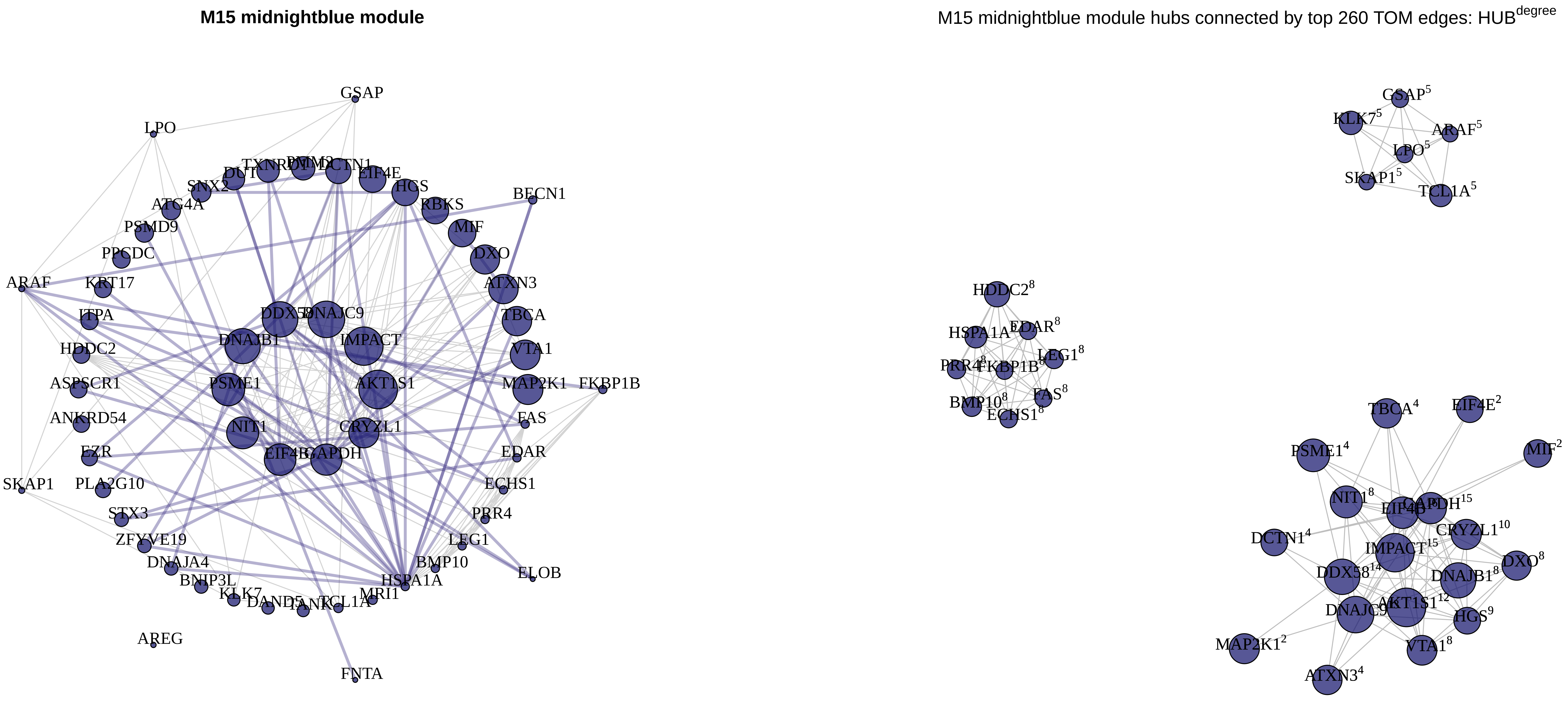
M15 midnightblue module
GSAP
LPO
BECN1
ARAF
FKBP1B
SKAP1
AREG
FNTA
HGS
RBKS
MIF
DXO
ATXN3
TBCA
VTA1
MAP2K1
FAS
EDAR
ECHS1
PRR4
LEG1
BMP10
ELOB
HSPA1A
MRI1
KLK7
BNIP3L
DNAJA4
ZFYVE19
STX3
PLA2G10
EZR
ANKRD54
ASPSCR1
HDDC2
ITPA
KRT17
PPCDC
PSMD9
ATG4A
SNX2
EIF4E
DNAJB1
IMPACT
AKT1S1
PSME1
NIT1
CRYZL1
GAPDH
M15 midnightblue module hubs connected by top 260 TOM edges: HUB^degree
GSAP5
KLK75
ARAF5
LPO5
SKAP15
TCL1A5
HDDC28
HSPA1A
EDAR8
LEG18
FAS8
ECHS18
BMP108
TBCA4
EIF4E2
MIF2
PSME14
NIT18
GAPDH15
CRYZL110
DCTN14
IMPACT15
DXO8
DDX5814
DNAJB18
AKT1S112
HGS9
VTA18
MAP2K12
ATXN34

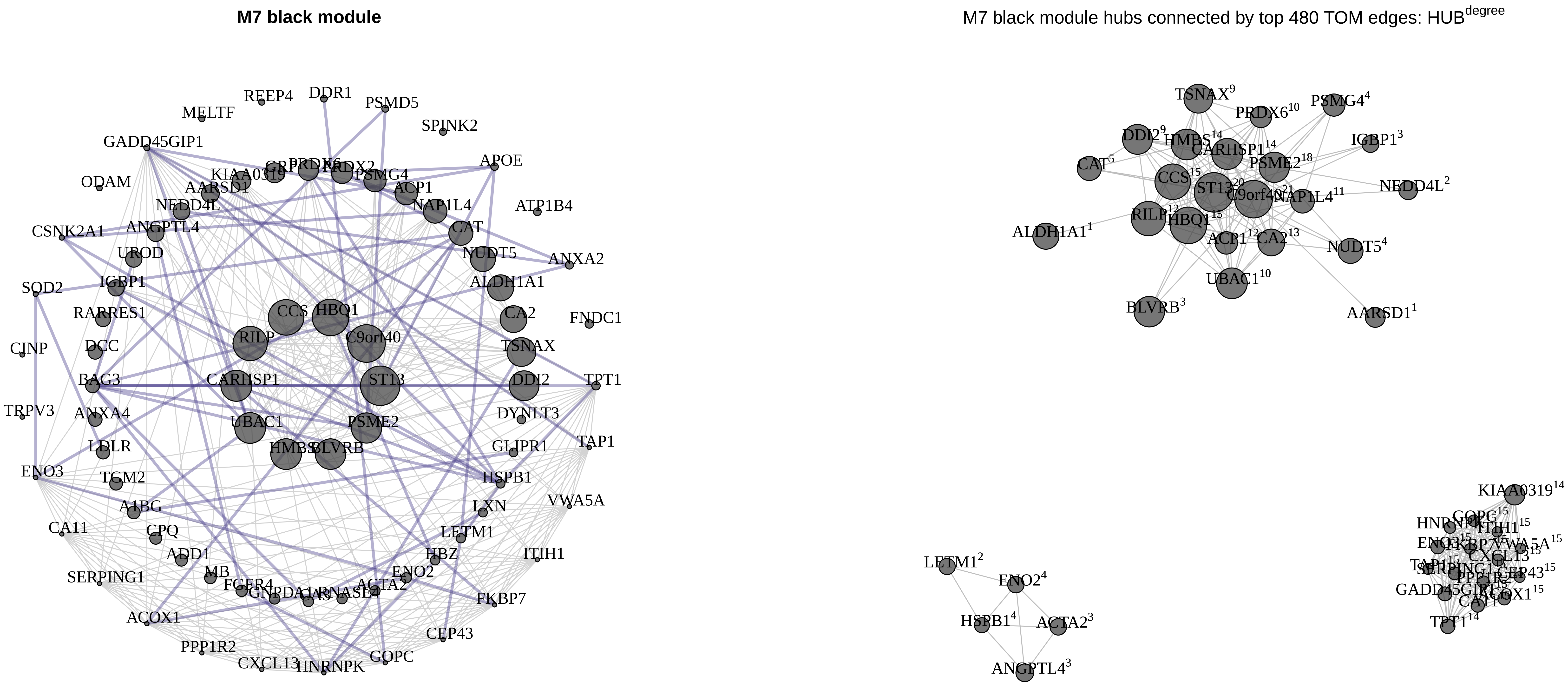
M7 black module
M7 black module hubs connected by top 480 TOM edges: HUB^degree
REEP4
DDR1
PSMD5
MELTF
SPINK2
GADD45GIP1
APOE
ODAM
AARSD1
ACP1
NAP1L4
ATP1B4
NEDD4L
CSNK2A1
ANGPTL4
CAT
UROD
NUDT5
ANXA2
SOD2
IGBP1
ALDH1A1
RARRES1
CCS
HBQ1
CA2
FNDC1
CINP
DCC
RILP
C9orf40
TSNAX
BAG3
CARHSP1
ST13
DDI2
TPT1
TRPV3
ANXA4
DYNLT3
UBAC1
PSME2
TAP1
LDLR
HMBS
BLVRB
GLIPR1
ENO3
TGM2
HSPB1
VWA5A
A1BG
LXN
CA11
CPQ
LETM1
ADD1
HBZ
ITIH1
SERPING1
MB
ENO2
FGFR4
ACTA2
FKBP7
ACOX1
CEP43
PPP1R2
CXCL13
HNRNPK
GOPC
TSNAX9
PRDX610
PSMG44
DDI29
HMBS14
IGBP13
CARHSP114
CAT5
PSME218
NEDD4L2
CCS15
ST1320
ALDH1A11
RILP12
NAP1L411
HBQ115
ACP112
CA213
NUDT54
UBAC110
BLVRB3
AARSD11
KIAA031914
LETM12
ENO24
HSPB14
ACTA23
ANGPTL43
TPT114

M24 darkgrey module

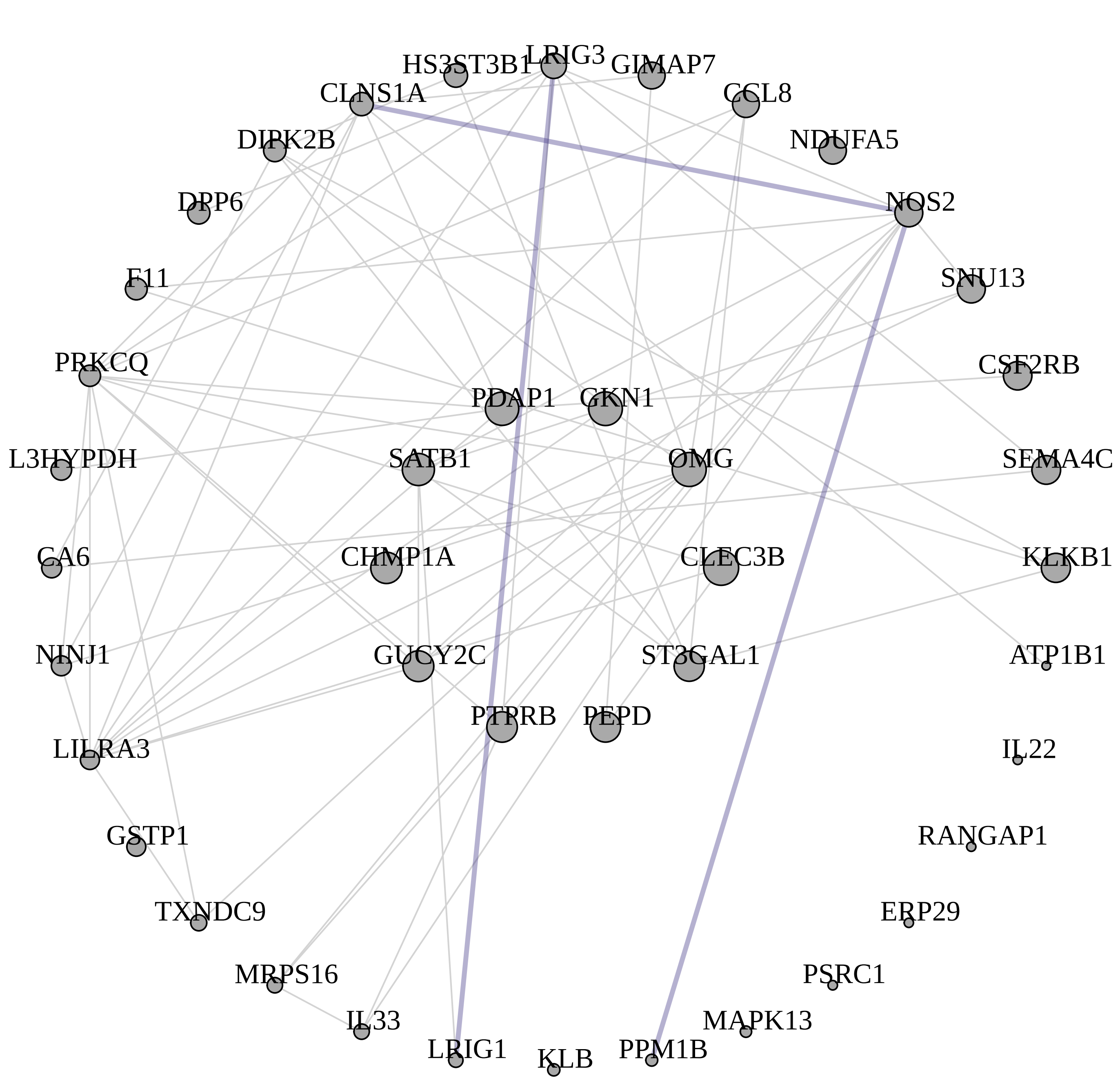

M24 darkgrey module hubs connected by top 126 TOM edges: HUB^{degree}

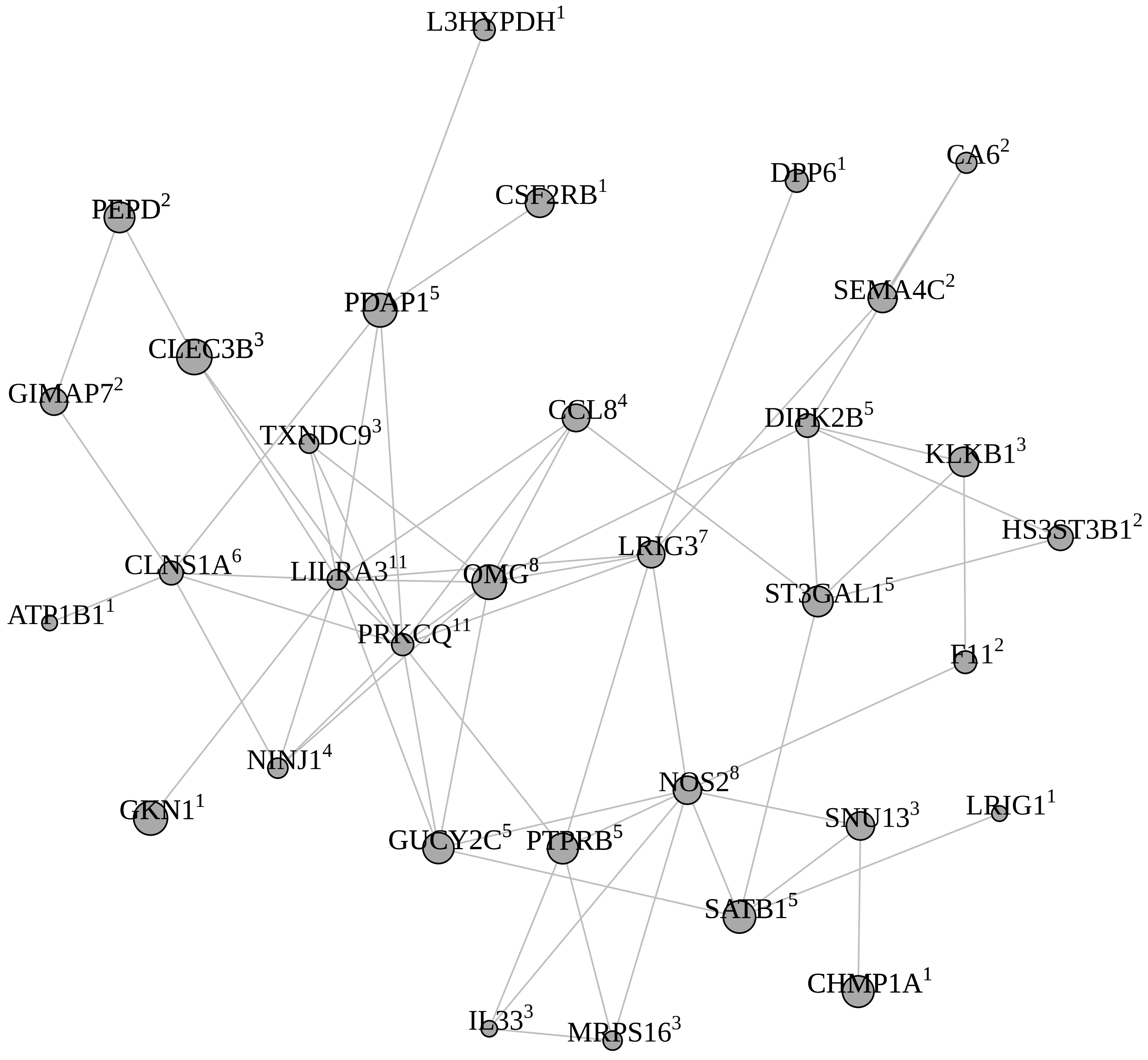

M20 royalblue module

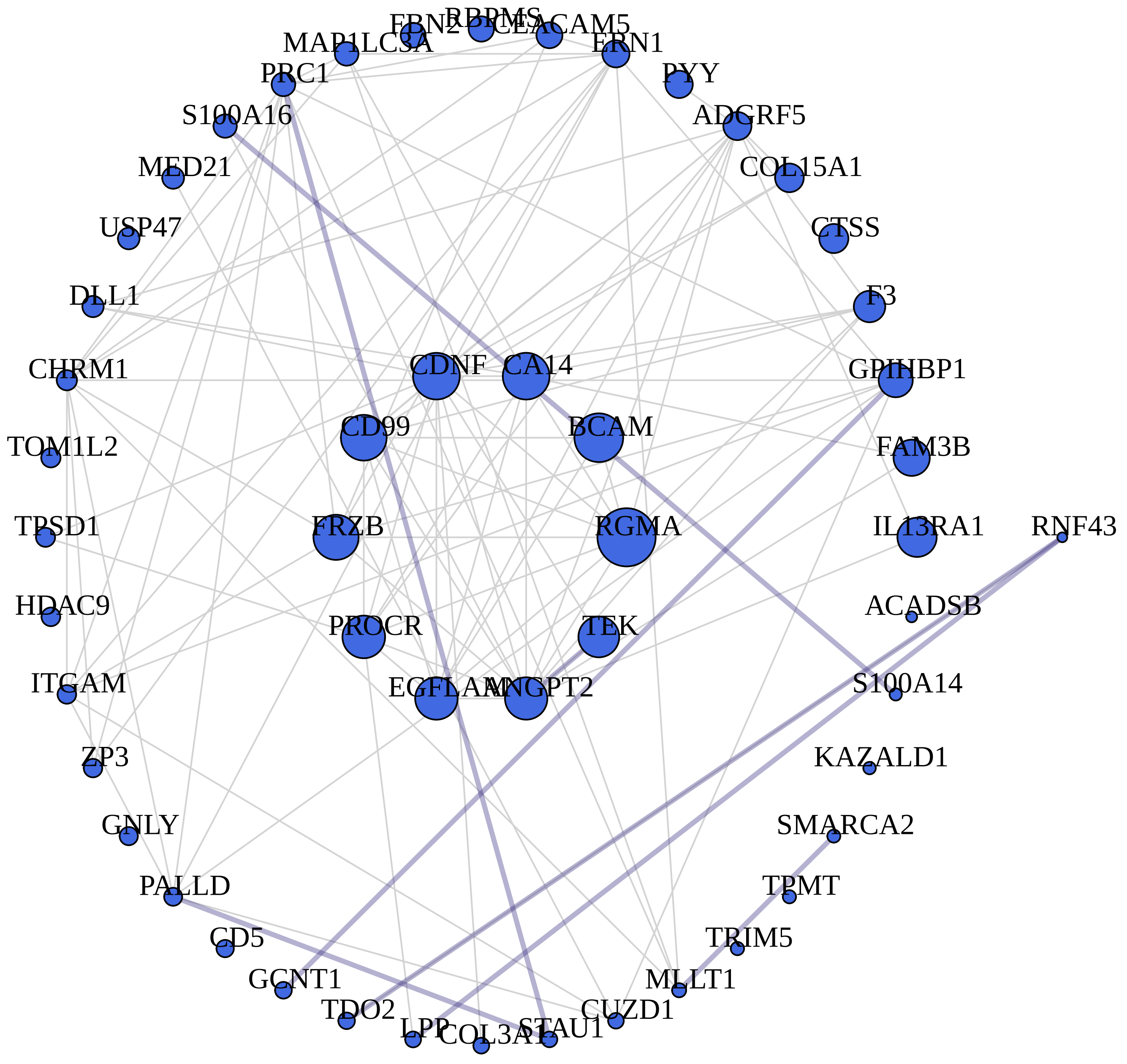

M20 royalblue module hubs connected by top 192 TOM edges: HUB^{degree}

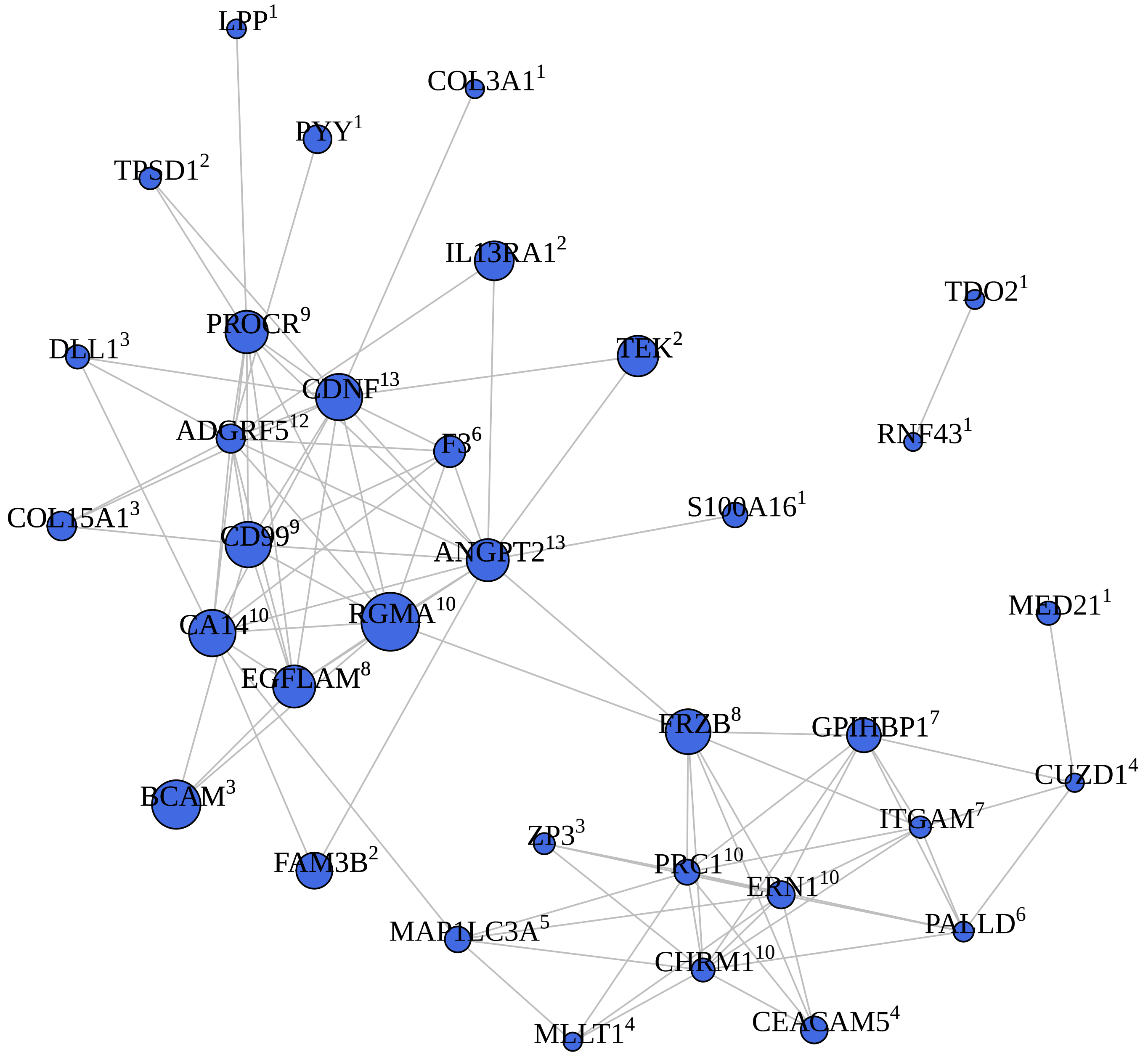

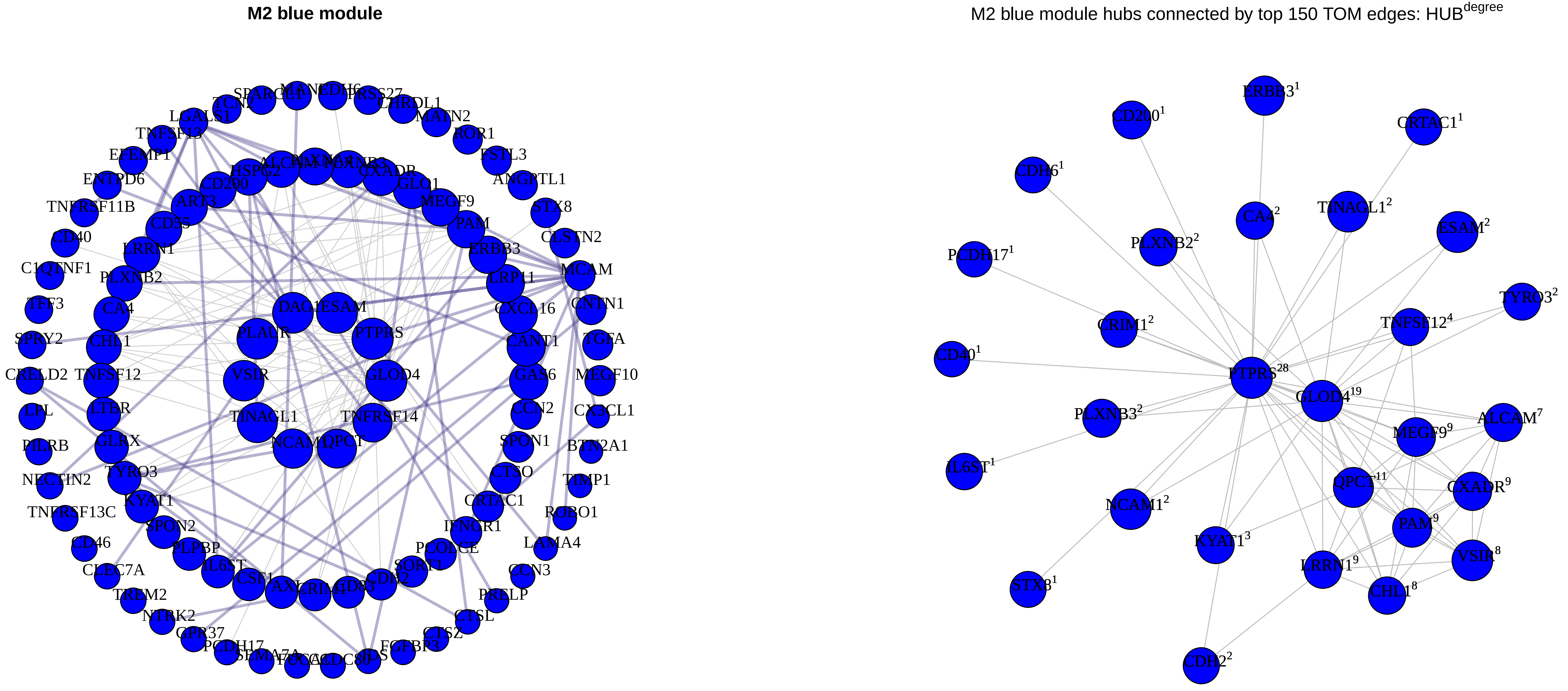

M2 blue module
M2 blue module hubs connected by top 150 TOM edges: HUB^degree
ERBB3 1
CD200 1
CRTAC1 1
CDH6 1
CA4 2
TINAGL1 2
ESAM 2
PCDH17 1
PLXNB2 2
TYRO3 2
CRIM1 2
TNFSF12 4
CD40 1
PTPRS 28
GLOD4 19
ALCAM 7
PLXNB3 2
MEGF9 9
IL6ST 1
QPCT 11
CXADR 9
NCAM1 2
PAM 9
KYAT1 3
VSIR 8
LRRN1 9
STX8 1
CHL1 8
CDH2 2

M25 orange module

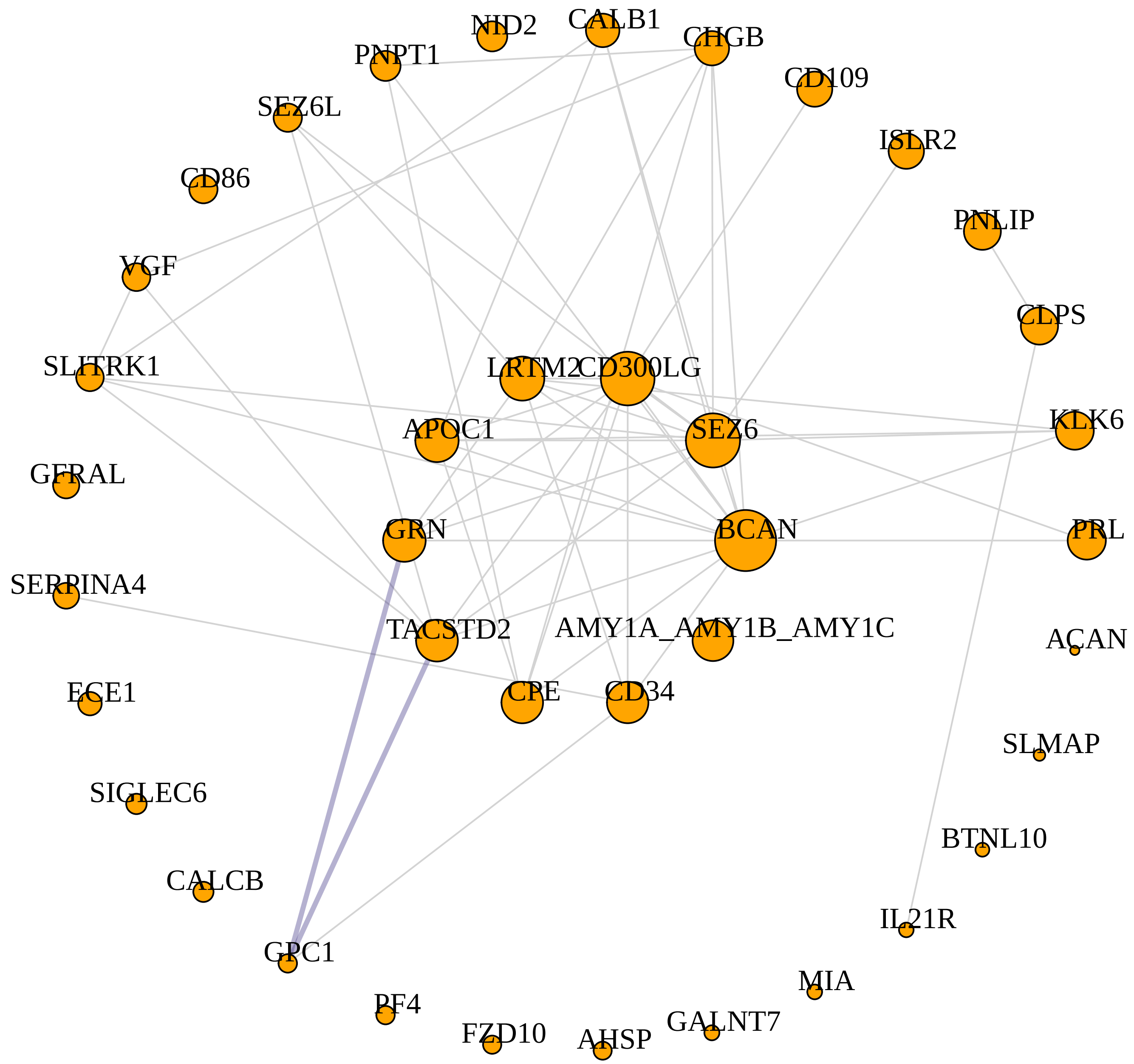

M25 orange module hubs connected by top 108 TOM edges: HUB[degree]

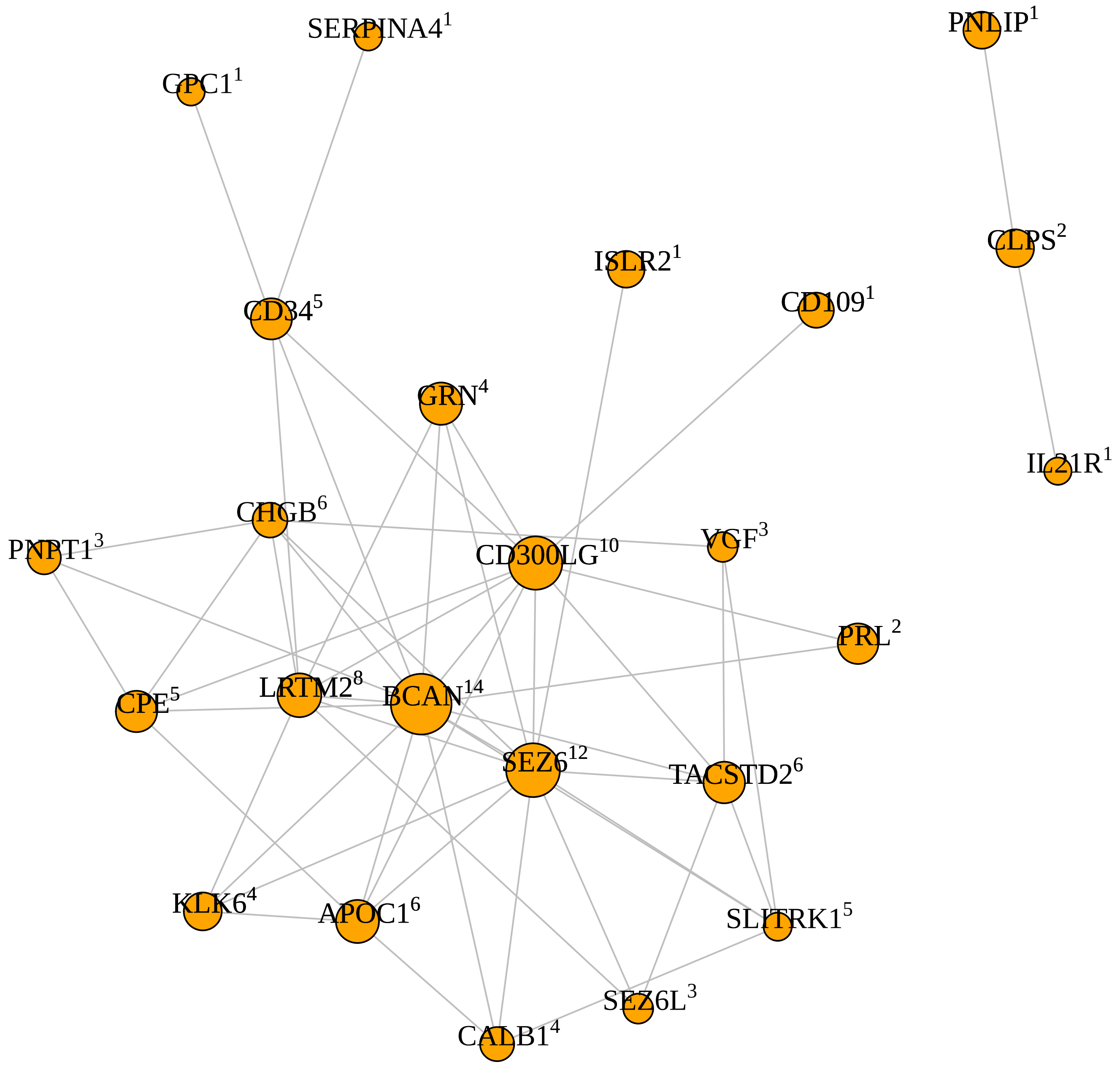

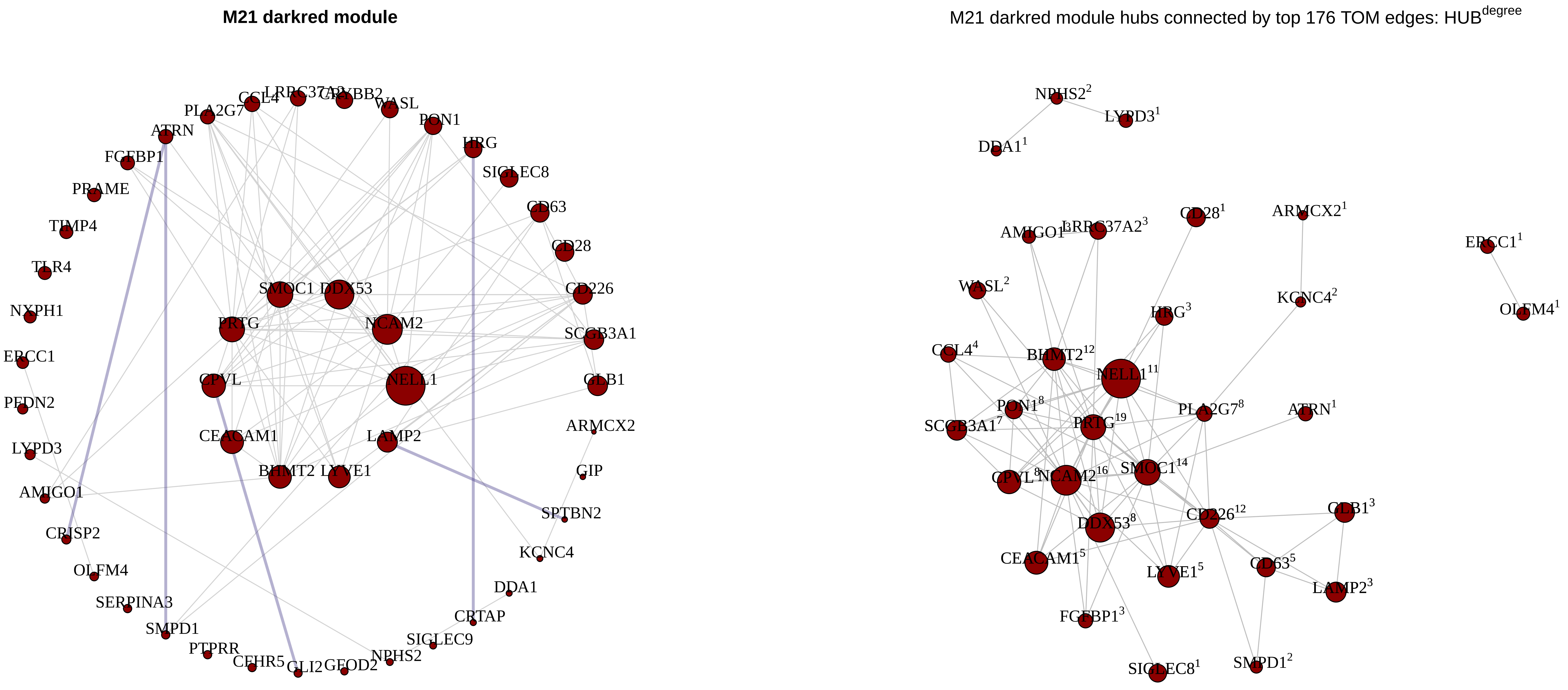

M21 darkred module
CCL4
LRRC37A2
CRYBB2
WASL
PLA2G7
PON1
ATRN
HRG
FCGBP1
SIGLEC8
PRAME
CD63
TIMP4
CD28
TLR4
SMOC1
DDX53
CD226
NXPH1
PRTG
NCAM2
SCGB3A1
ERCC1
CPVL
NELL1
CLB1
PFDN2
ARMCX2
CEACAM1
LAMP2
LYPD3
GIP
BHMT2
LYVE1
AMIGO1
SPTBN2
CRISP2
KCNC4
OLFM4
DDA1
SERPINA3
CRTAP
SMPD1
SIGLEC9
PTPRR
NPHS2
CFHR5
GLI2
GFOD2
M21 darkred module hubs connected by top 176 TOM edges: HUB^{degree}
NPHS2^{2}
LYPD3^{1}
DDA1^{1}
CD28^{1}
ARMCX2^{1}
AMIGO1^{1}
LRRC37A2^{3}
ERCC1^{1}
WASL^{2}
KCNC4^{2}
HRG^{3}
OLFM4^{1}
CCL4^{4}
BHMT2^{12}
NELL1^{11}
PON1^{8}
PLA2G7^{8}
ATRN^{1}
SCGB3A1^{7}
PRTG^{19}
SMOC1^{14}
CPVL^{8}
NCAM2^{16}
CD226^{12}
CLB1^{3}
DDX53^{8}
CEACAM1^{5}
CD63^{5}
LYVE1^{5}
LAMP2^{3}
FCGBP1^{3}
SIGLEC8^{1}
SMPD1^{2}

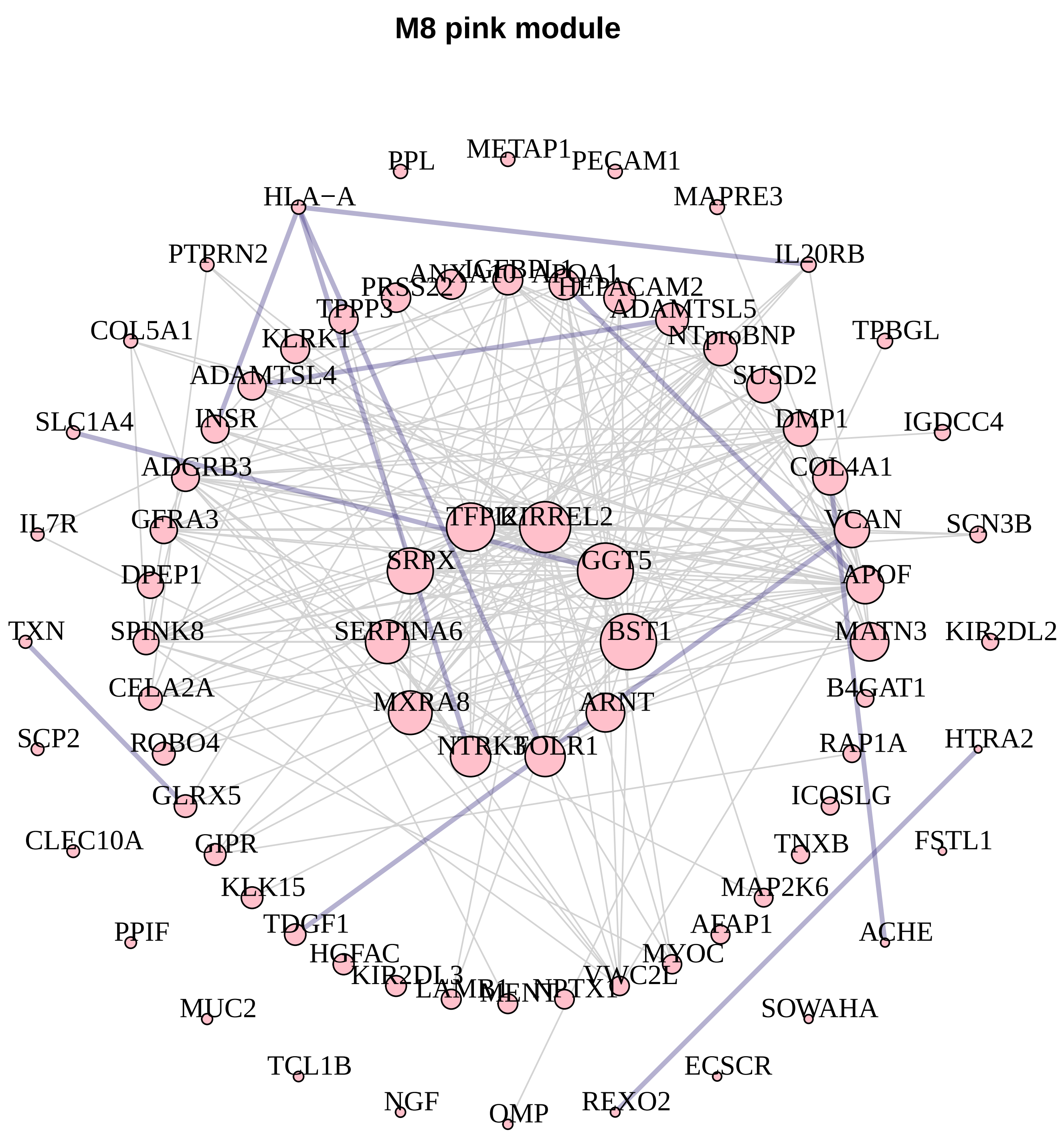
M8 pink module
PPL
METAP1
PECAM1
HLA−A
MAPRE3
PTPRN2
IL20RB
TPPP3
KLRK1
ADAMTSL5
NTproBNP
TPBGL
COL5A1
ADAMTSL4
SUSD2
SLC1A4
INSR
DMP1
IGDCC4
ADGRB3
COL4A1
IL7R
GFRA3
VCAN
SCN3B
DPEP1
SRPX
GGT5
APOF
TXN
SPINK8
SERPINA6
BST1
MATN3
KIR2DL2
CELA2A
B4GAT1
SCP2
ROBO4
MXRA8
ARNT
RAP1A
HTRA2
GLRX5
ICOSLG
CLEC10A
GIPR
TNXB
FSTL1
KLK15
MAP2K6
PPIF
TDGF1
AFAP1
ACHE
HGFAC
MYOC
KIR2DL3
MUC2
SOWAHA
TCL1B
ECSCR
NGF
OMP
REXO2
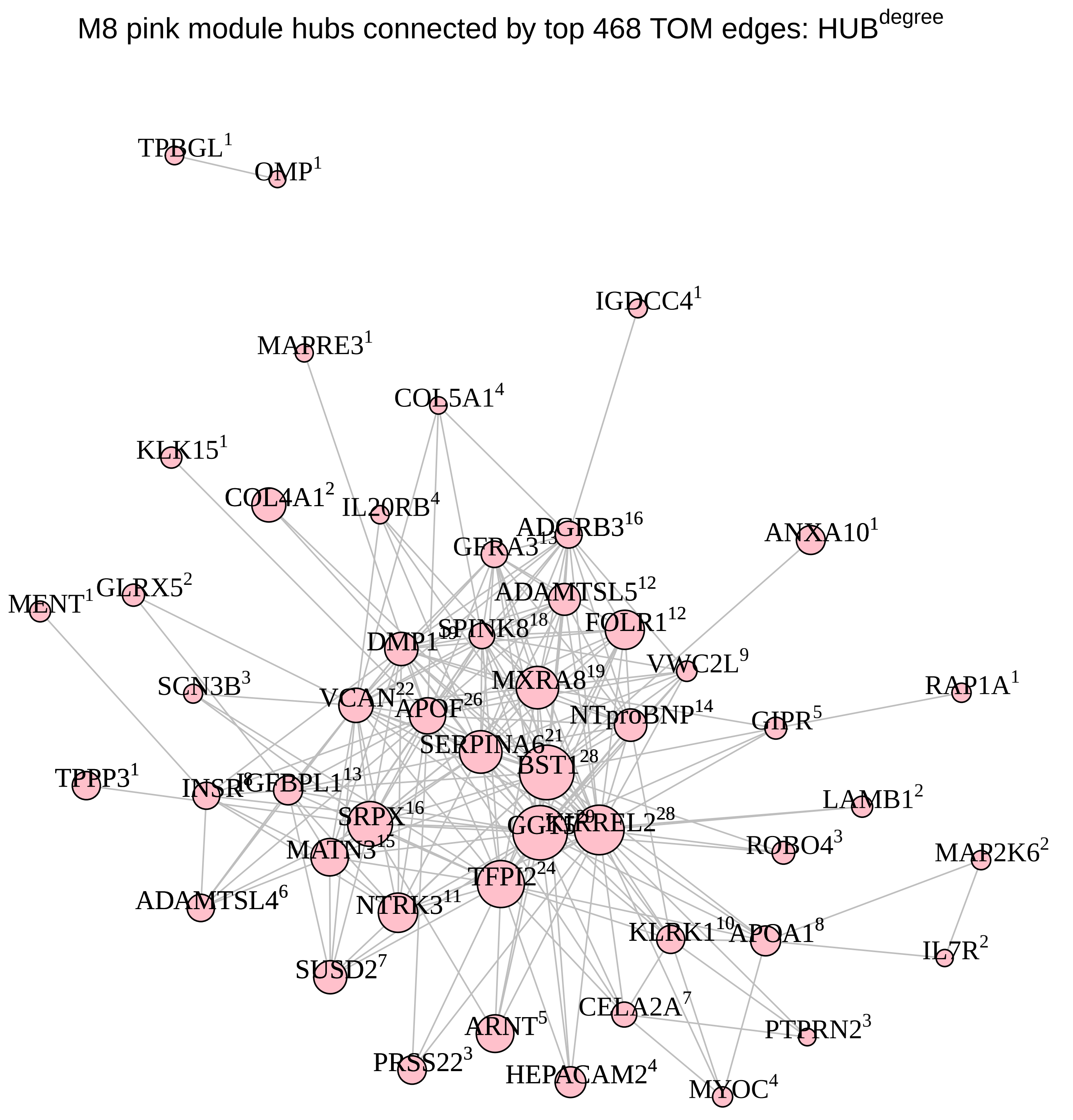
M8 pink module hubs connected by top 468 TOM edges: HUBdegree
TPBGL1
OMP1
IGDCC41
MAPRE31
COL5A14
KLK151
COL4A12
IL20RB4
ADGRB316
GFRA313
ANXA101
ADAMTSL512
MENT1
GLRX52
FOLR112
SPINK818
VWC2L9
SCN3B3
MXRA819
RAP1A1
VCAN22
APOF26
NTproBNP14
GIPR5
SERPINA621
BST128
TPPP31
IGFBPL113
LAMB12
SRPX16
KIRREL228
ROBO43
MATN315
TFPI224
MAP2K62
ADAMTSL46
NTRK311
KLRK110
APOA18
SUSD27
IL7R2
CELA2A7
ARNT5
PTPRN23
PRSS223
HEPACAM24
MYOC4

M19 lightyellow module

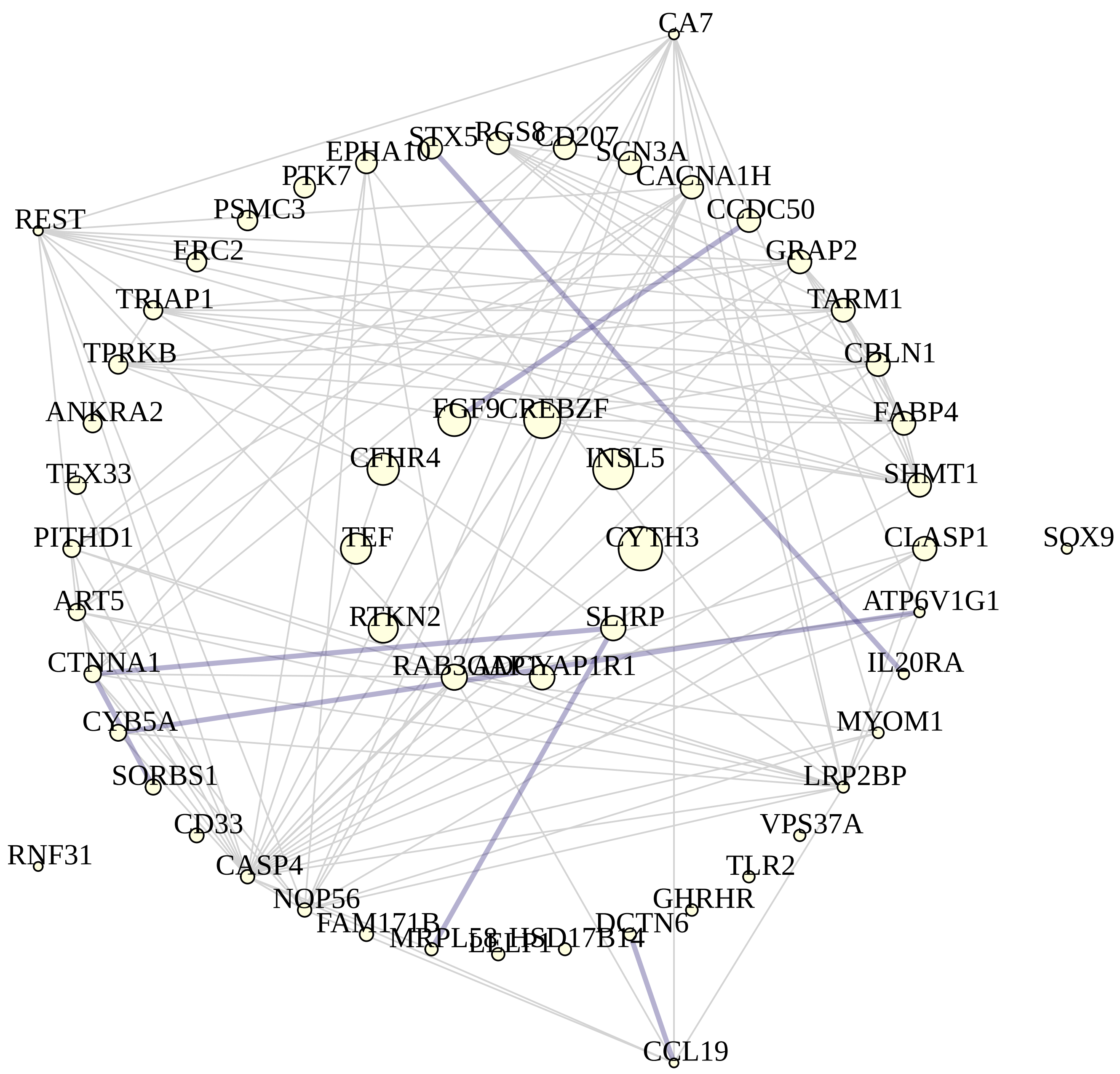

M19 lightyellow module hubs connected by top 224 TOM edges: HUB^{degree}

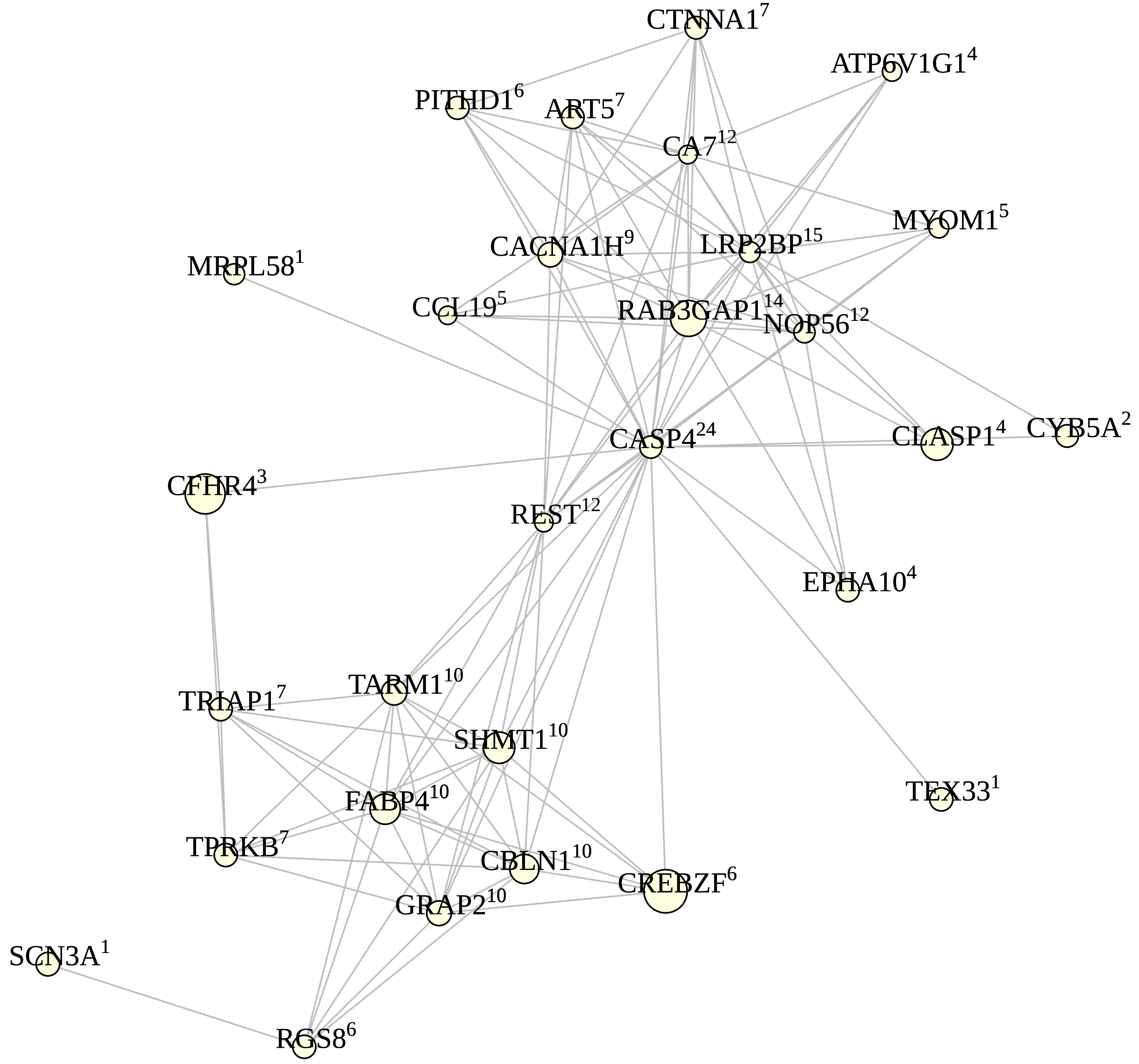

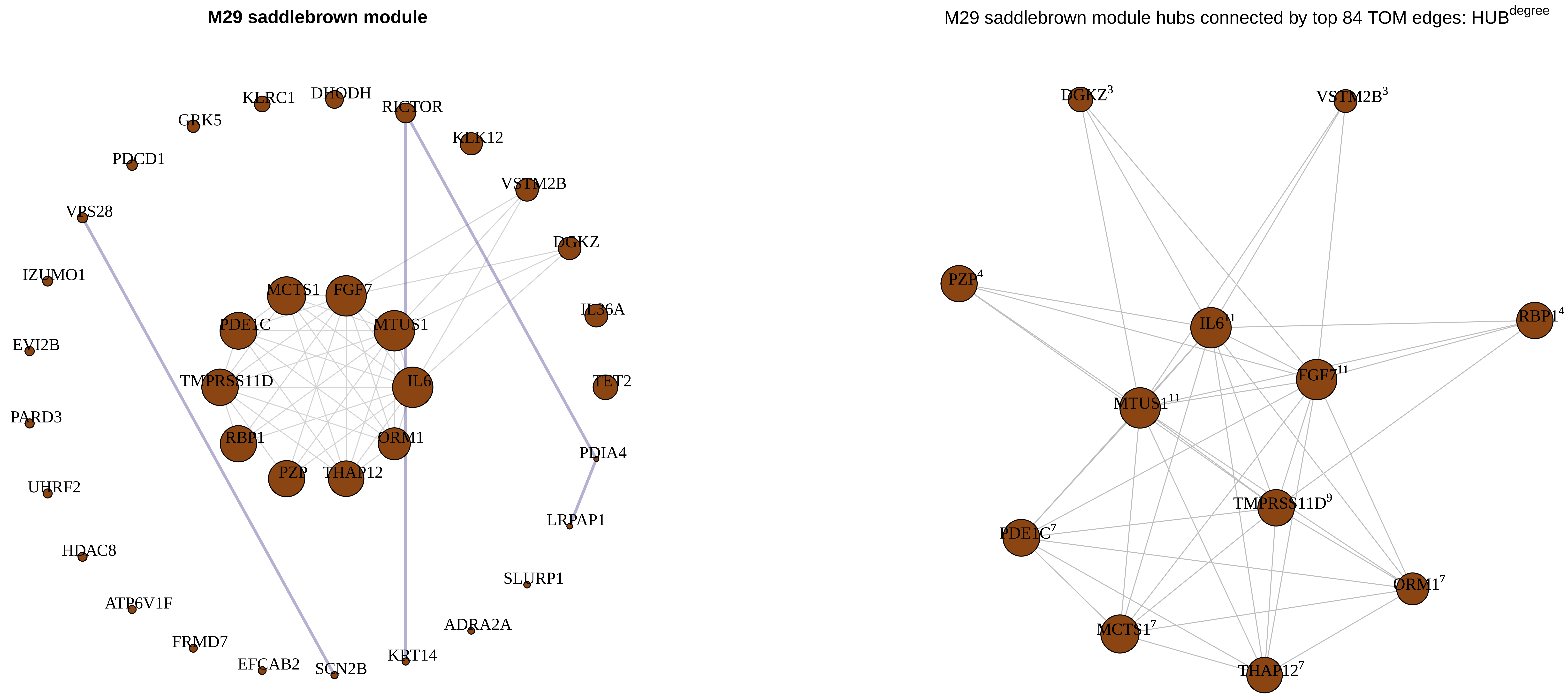
M29 saddlebrown module
KLRC1
DHODH
RICTOR
GRK5
KLK12
PDCD1
VSTM2B
VPS28
DGKZ
IZUMO1
MCTS1
FGF7
IL36A
PDE1C
MTUS1
EVI2B
TMPRSS11D
IL6
TET2
PARD3
RBP1
ORM1
PDIA4
PZP
THAP12
UHRF2
LRPAP1
HDAC8
SLURP1
ATP6V1F
ADRA2A
FRMD7
KRT14
EFCAB2
SCN2B
M29 saddlebrown module hubs connected by top 84 TOM edges: HUB^{degree}
DGKZ^{3}
VSTM2B^{3}
PZP^{4}
IL6^{11}
RBP1^{4}
FGF7^{11}
MTUS1^{11}
TMPRSS11D^{9}
PDE1C^{7}
ORM1^{7}
MCTS1^{7}
THAP12^{7}

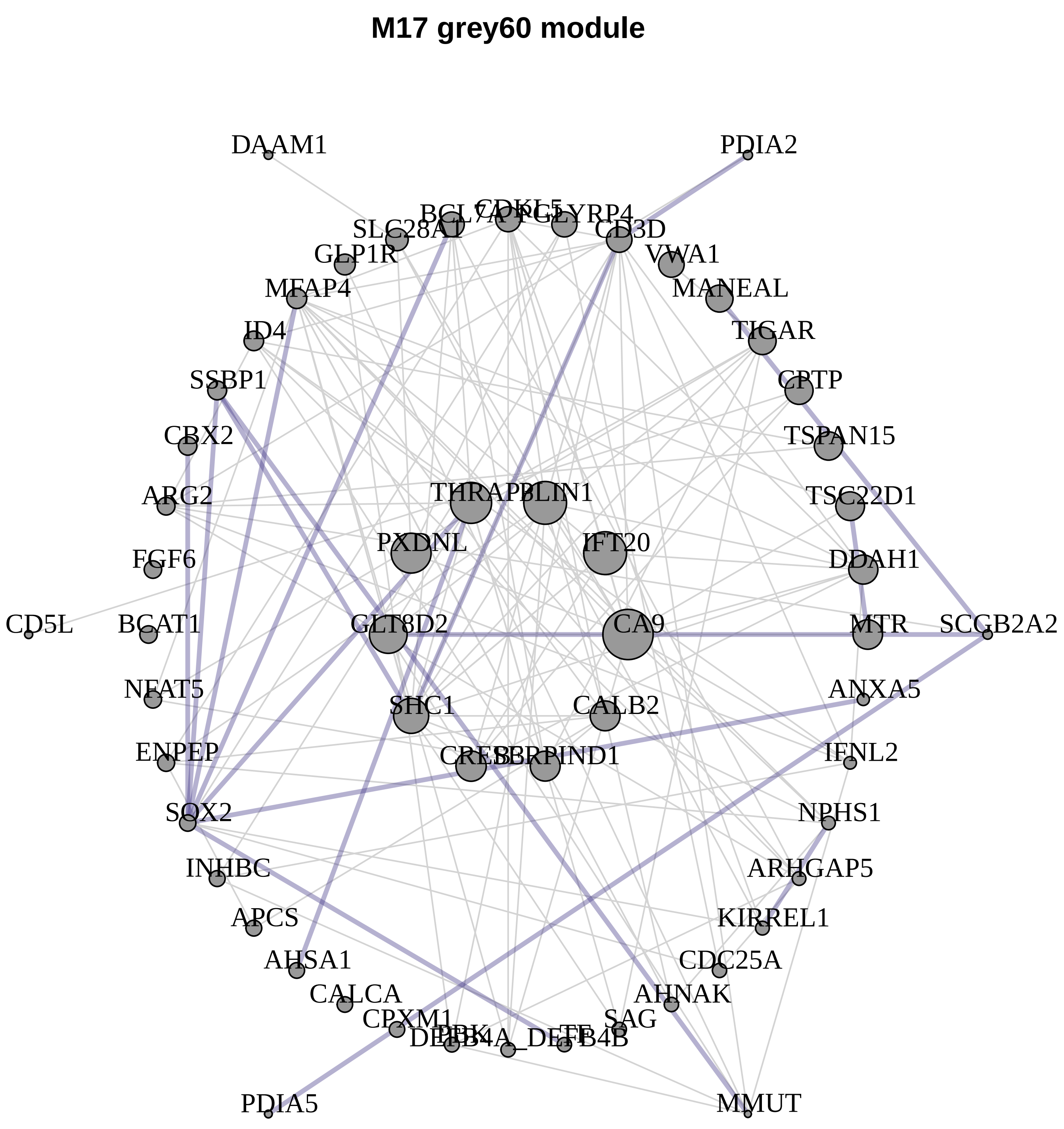
M17 grey60 module
DAAM1
PDIA2
SLC28A1
GLP1R
MFAP4
ID4
SSBP1
CBX2
ARG2
FGF6
CD5L
BCAT1
NFAT5
ENPEP
SOX2
INHBC
APCS
AHSA1
CALCA
CPXM1
PDIA5
CD3D
VWA1
MANEAL
TIGAR
CPTP
TSPAN15
TSC22D1
DDAH1
MTR
SCGB2A2
ANXA5
IFNL2
NPHS1
ARHGAP5
KIRREL1
CDC25A
AHNAK
SAG
MMUT
PXDNL
IFT20
CA9
GLT8D2
SHC1
CALB2
SERPIND1

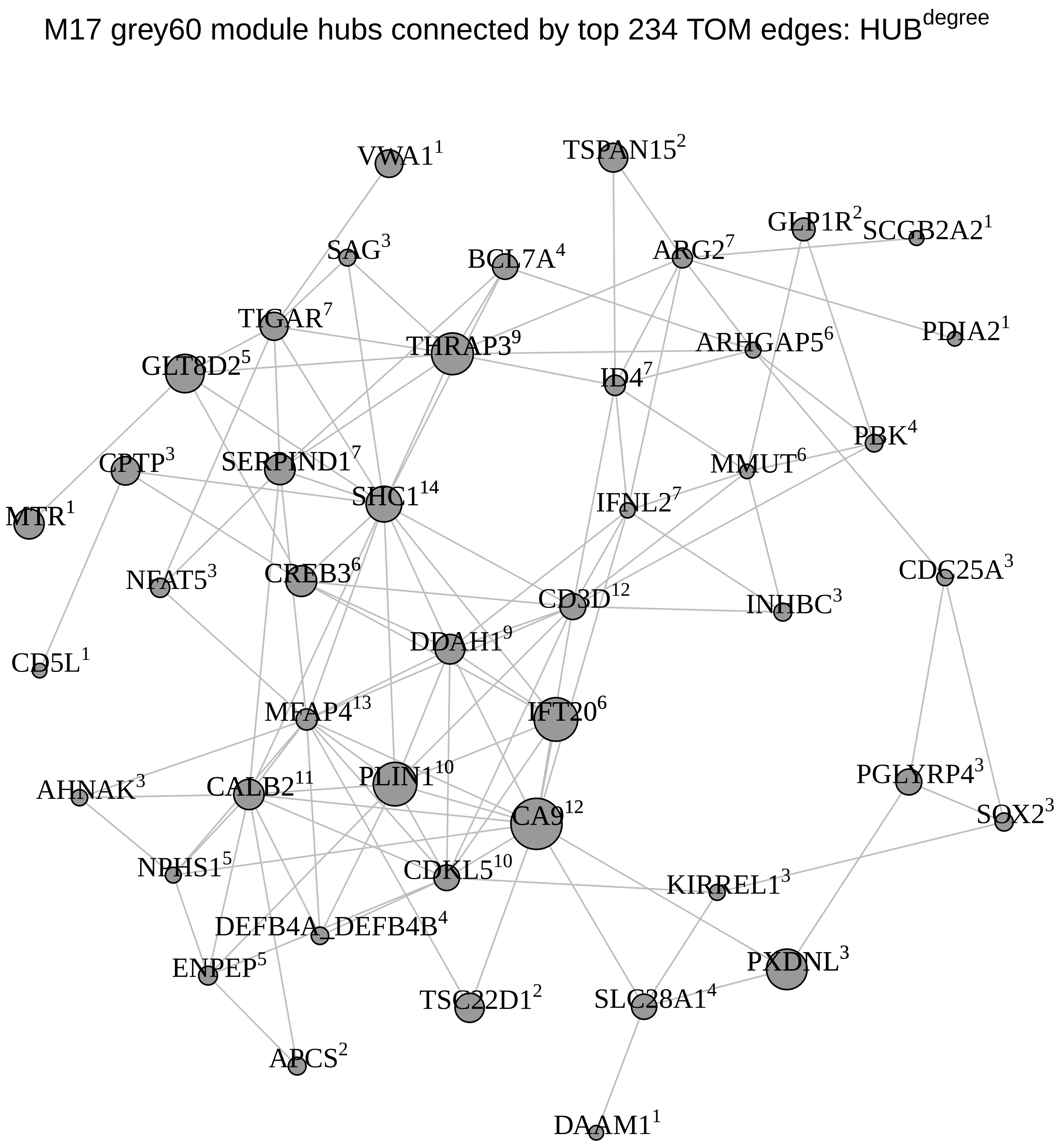
M17 grey60 module hubs connected by top 234 TOM edges: HUB^{degree}
VWA1^{1}
TSPAN15^{2}
SAG^{3}
BCL7A^{4}
ARG2^{7}
GLP1R^{2}
SCGB2A2^{1}
PDIA2^{1}
TIGAR^{7}
THRAP3^{9}
GLT8D2^{5}
ARHGAP5^{6}
ID4^{7}
PBK^{4}
CPTP^{3}
SERPIND1^{7}
SHC1^{14}
MMUT^{6}
MTR^{1}
IFNL2^{7}
NFAT5^{3}
CREB3^{6}
CD3D^{12}
INHBC^{3}
CDC25A^{3}
CD5L^{1}
DDAH1^{9}
MFAP4^{13}
IFT20^{6}
AHNAK^{3}
CALB2^{11}
PLIN1^{10}
CA9^{12}
PGLYRP4^{3}
SOX2^{3}
NPHS1^{5}
CDKL5^{10}
KIRREL1^{3}
DEFB4A
DEFB4B^{4}
ENPEP^{5}
PXDNL^{3}
TSC22D1^{2}
SLC28A1^{4}
APCS^{2}
DAAM1^{1}

M9 magenta module

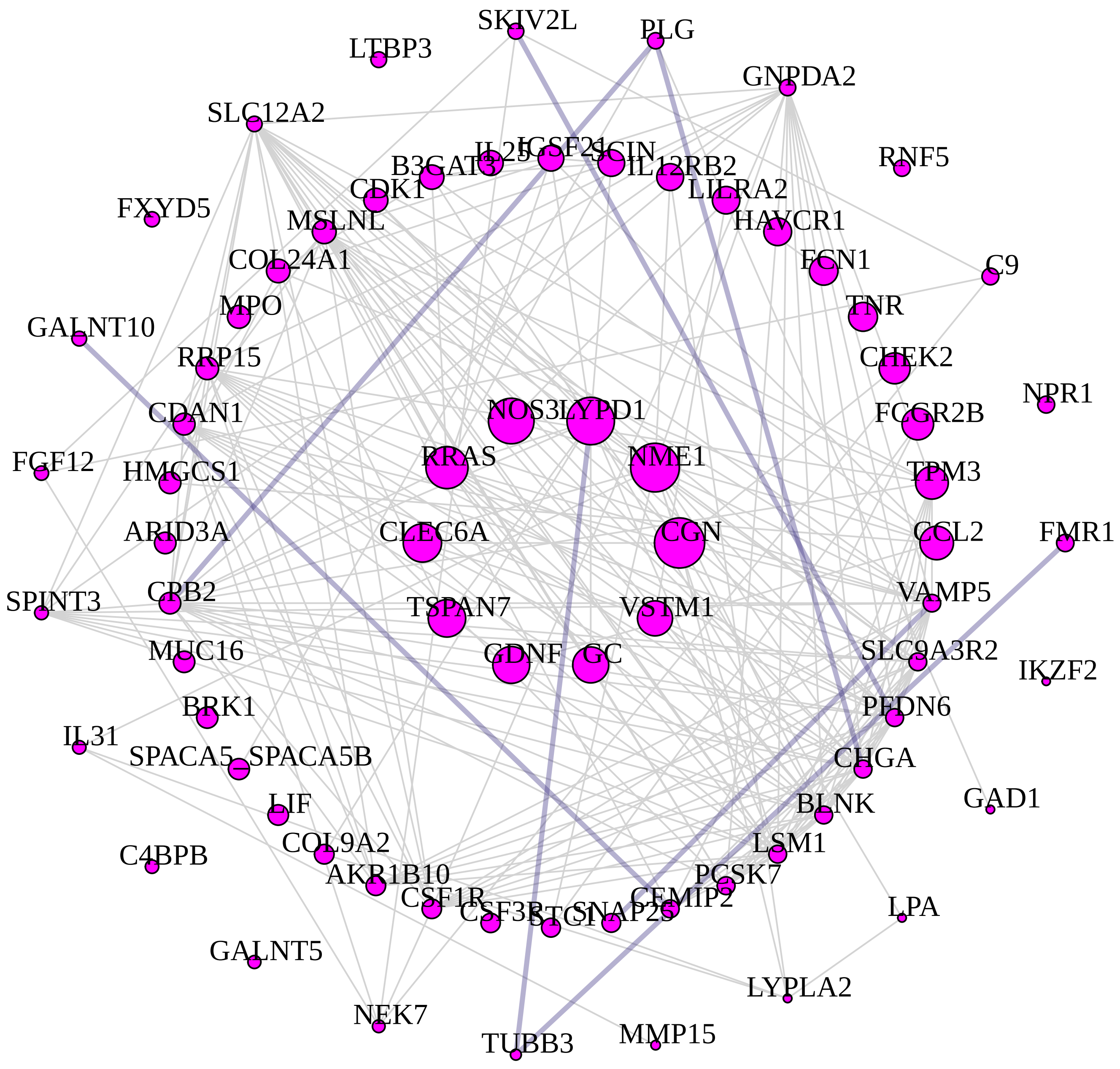

M9 magenta module hubs connected by top 408 TOM edges: HUB^{degree}

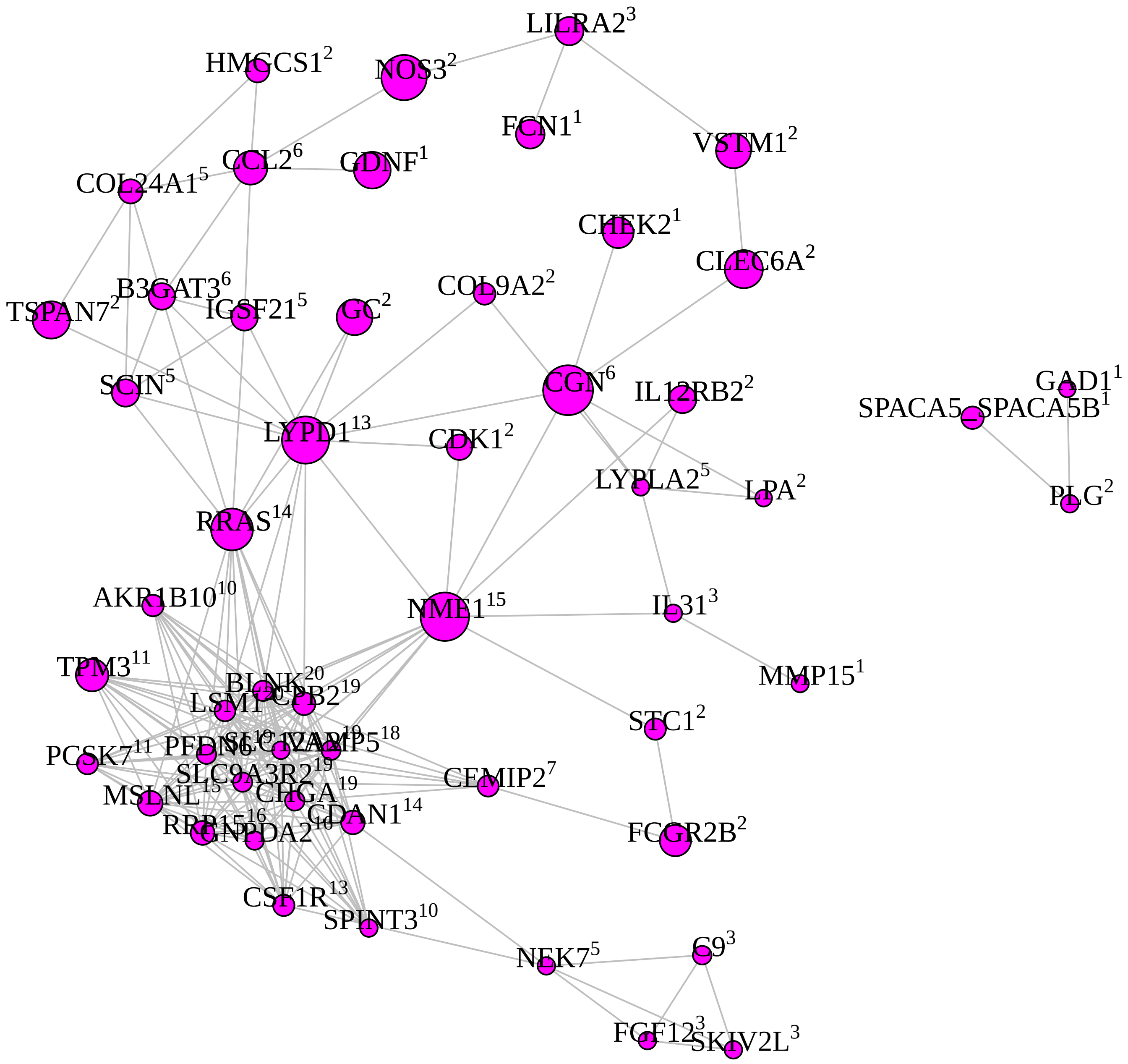

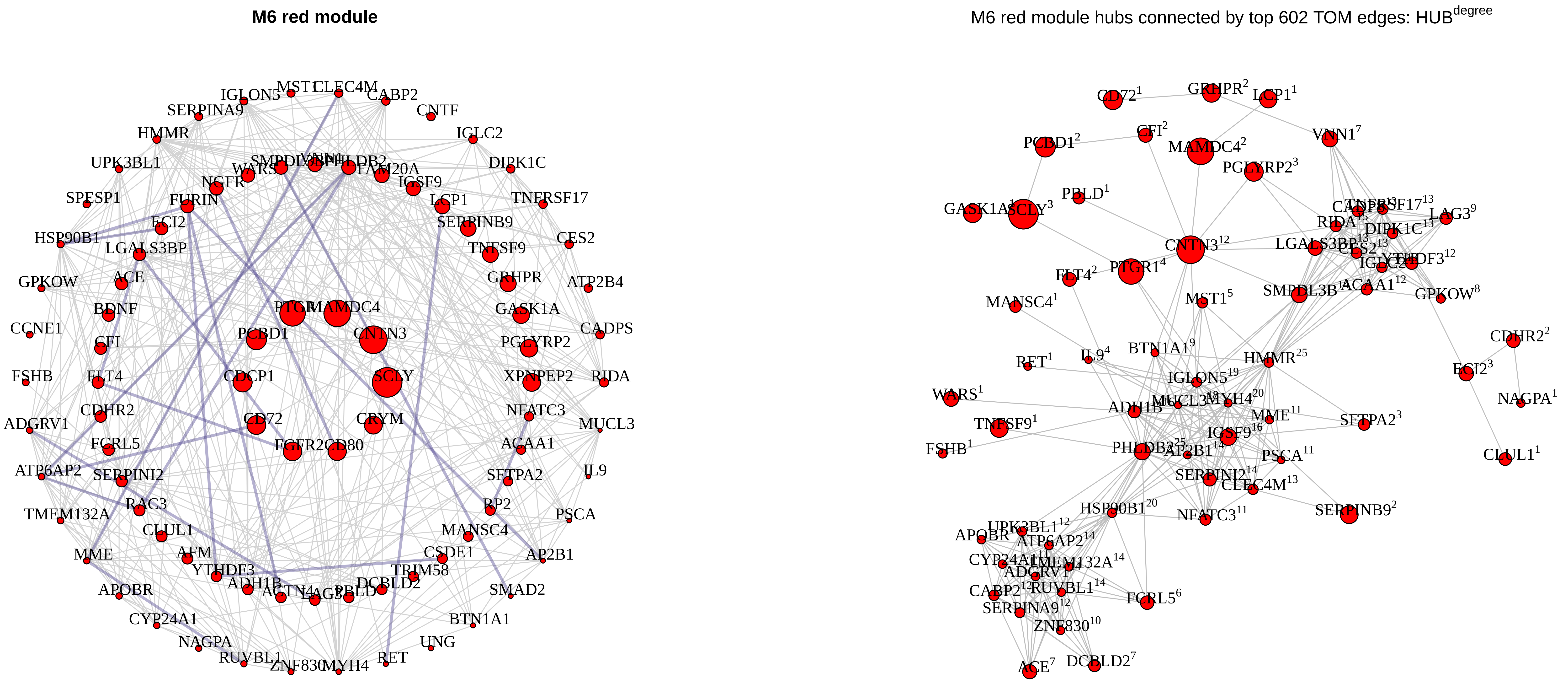

M6 red module
M6 red module hubs connected by top 602 TOM edges: HUB^{degree}
IGLON5
MST1
CLEC4M
CABP2
SERPINA9
CNTF
HMMR
IGLC2
UPK3BL1
DIPK1C
WARS
NGFR
FAM20A
IGSF9
SPESP1
FURIN
LCP1
TNFRSF17
HSP90B1
ECI2
SERPINB9
CES2
LGALS3BP
TNFSF9
GPKOW
ACE
GRHPR
ATP2B4
BDNF
GASK1A
CCNE1
PCBD1
CNTN3
CADPS
CFI
PGLYRP2
FSHB
FLT4
CDCP1
SCLY
XPNPEP2
RIDA
CDHR2
NFATC3
ADGRV1
CD72
CRYM
MUCL3
FCRL5
ACAA1
ATP6AP2
SERPINI2
IL9
SFTPA2
TMEM132A
RAC3
RP2
PSCA
CLUL1
MANSC4
MME
AFM
CSDE1
AP2B1
YTHDF3
TRIM58
ADH1B
DCBLD2
APOBR
SMAD2
CYP24A1
BTN1A1
NAGPA
UNG
RUVBL1
ZNF830
MYH4
RET
CD72^{1}
GRHPR^{2}
LCP1^{1}
VNN1^{7}
PCBD1^{2}
CFI^{2}
MAMDC4^{2}
PGLYRP2^{3}
PBLD^{1}
LAG3^{9}
CNTN3^{12}
FLT4^{2}
PTGR1^{4}
ACAA1^{12}
GPKOW^{8}
MANSC4^{1}
MST1^{5}
CDHR2^{2}
RET^{1}
IL9^{4}
BTN1A1^{9}
HMMR^{25}
ECI2^{3}
IGLON5^{19}
WARS^{1}
MYH4^{20}
NAGPA^{1}
MME^{11}
TNFSF9^{1}
IGSF9^{16}
SFTPA2^{3}
FSHB^{1}
AP2B1^{14}
PSCA^{11}
CLUL1^{1}
SERPINI2^{14}
CLEC4M^{13}
HSP90B1^{20}
NFATC3^{11}
SERPINB9^{2}
UPK3BL1^{12}
ATP6AP2^{14}
TMEM132A^{14}
RUVBL1^{14}
FCRL5^{6}
SERPINA9^{12}
ZNF830^{10}
ACE^{7}
DCBLD2^{7}

M13 salmon module

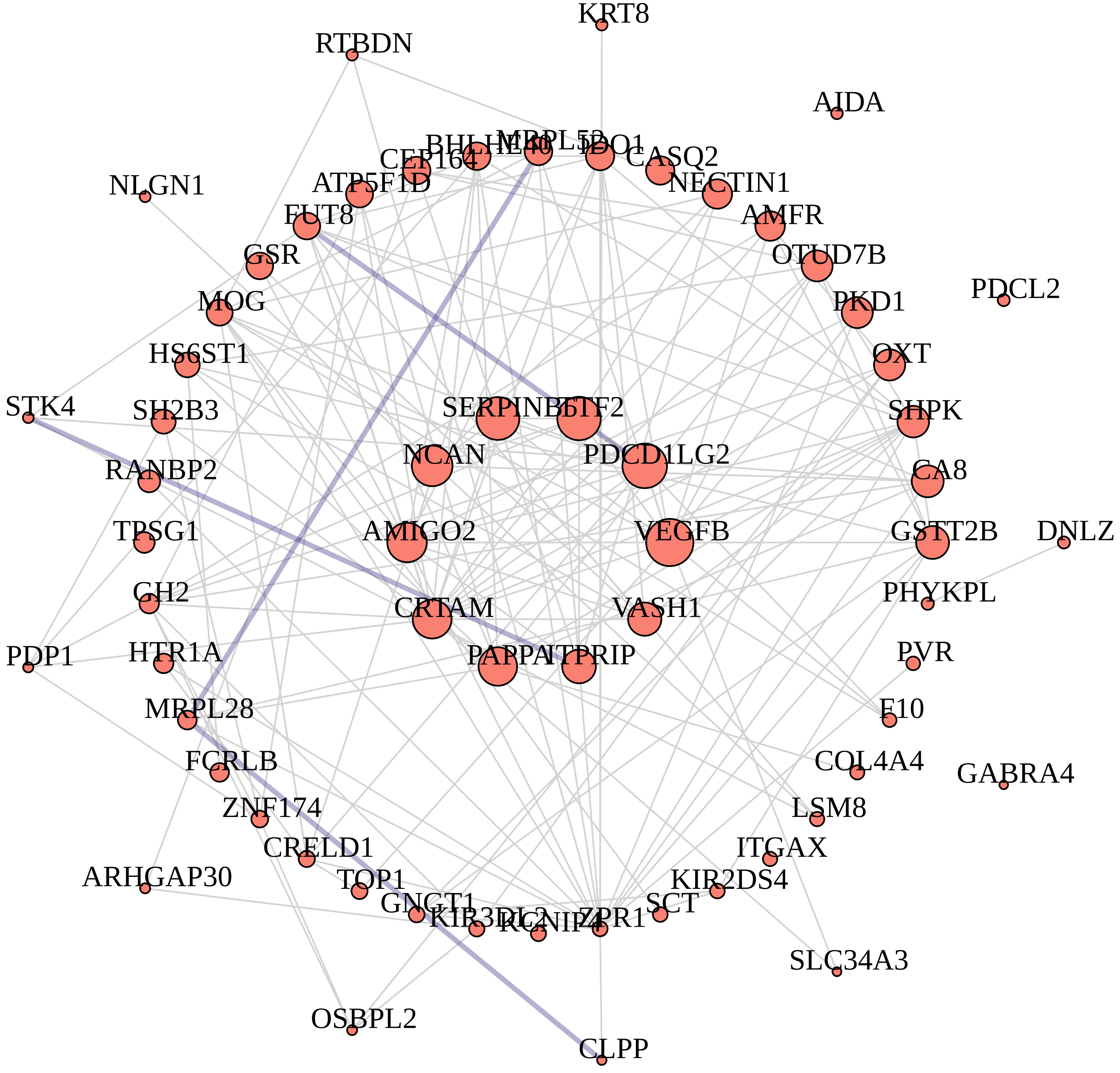

M13 salmon module hubs connected by top 300 TOM edges: HUB^{degree}

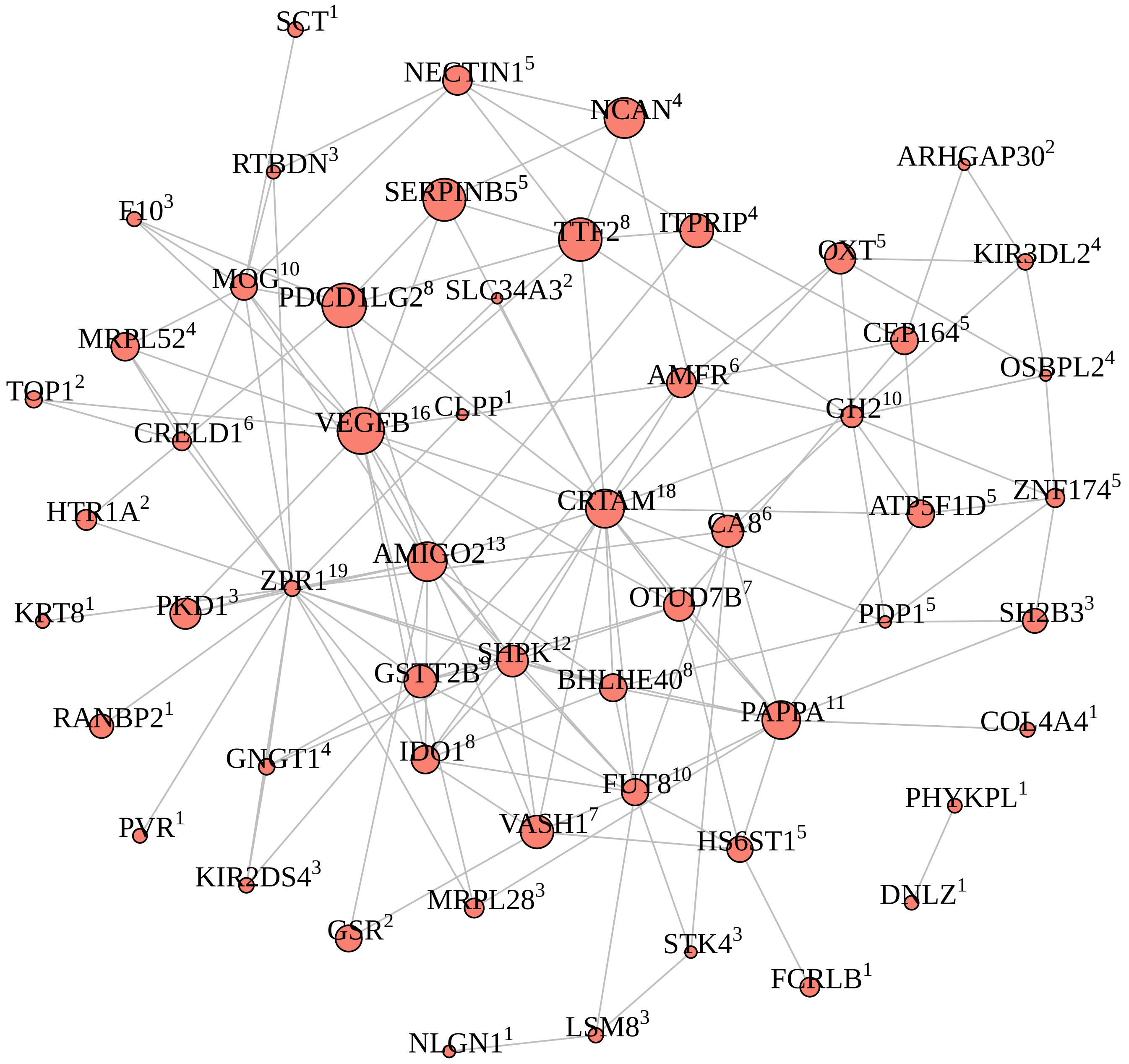

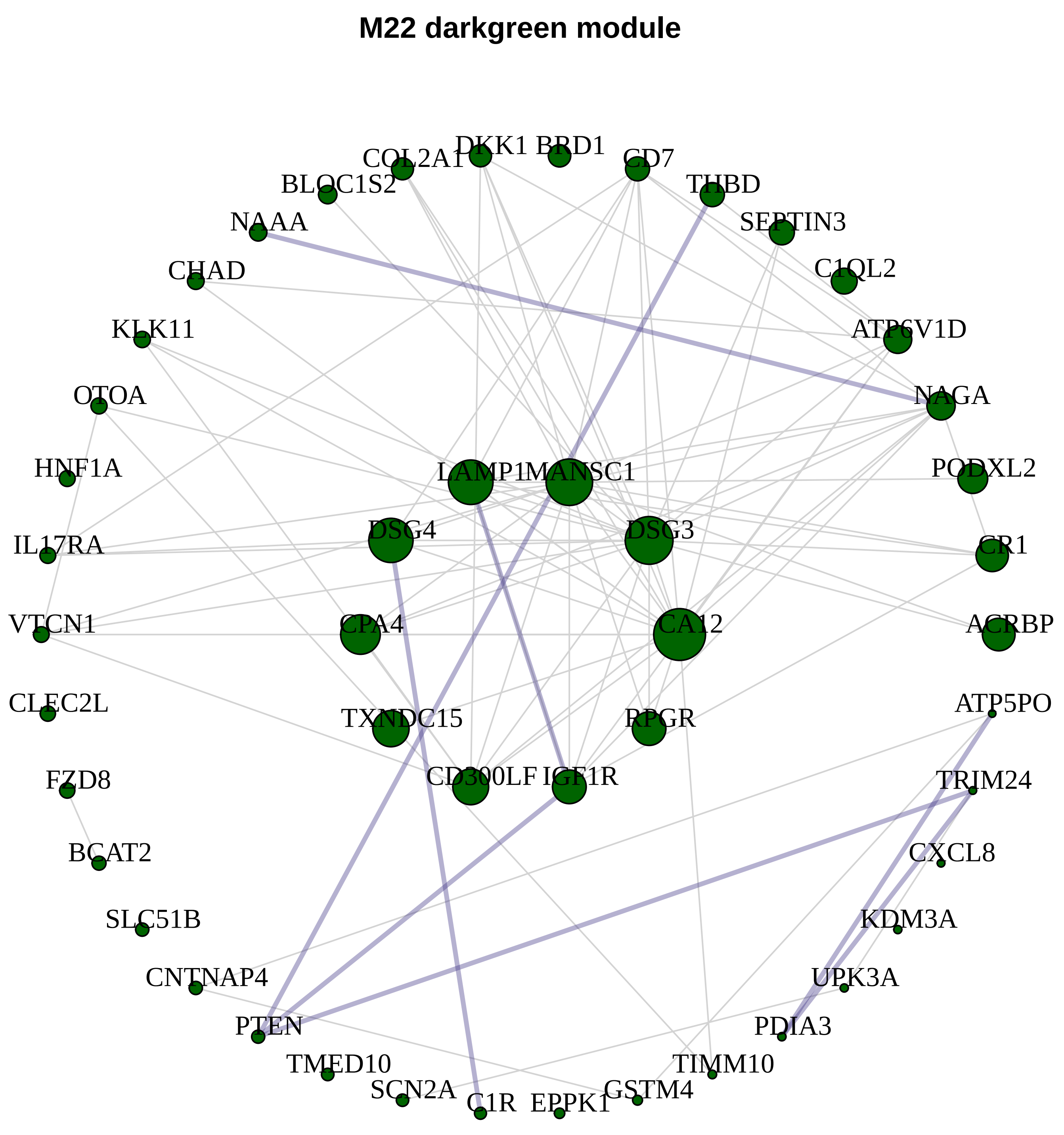
M22 darkgreen module
NAAA
BLOC1S2
COL2A1
DKK1
BRD1
CD7
THBD
SEPTIN3
C1QL2
ATP6V1D
NAGA
PODXL2
CR1
ACRBP
ATP5PO
TRIM24
CXCL8
KDM3A
UPK3A
PDIA3
TIMM10
GSTM4
EPPK1
C1R
SCN2A
TMED10
PTEN
CNTNAP4
SLC51B
BCAT2
FZD8
CLEC2L
VTCN1
IL17RA
HNF1A
OTOA
KLK11
CHAD
LAMP1
MANSC1
DSG4
DSG3
CPA4
CA12
TXNDC15
RPGR
CD300LF
IGF1R

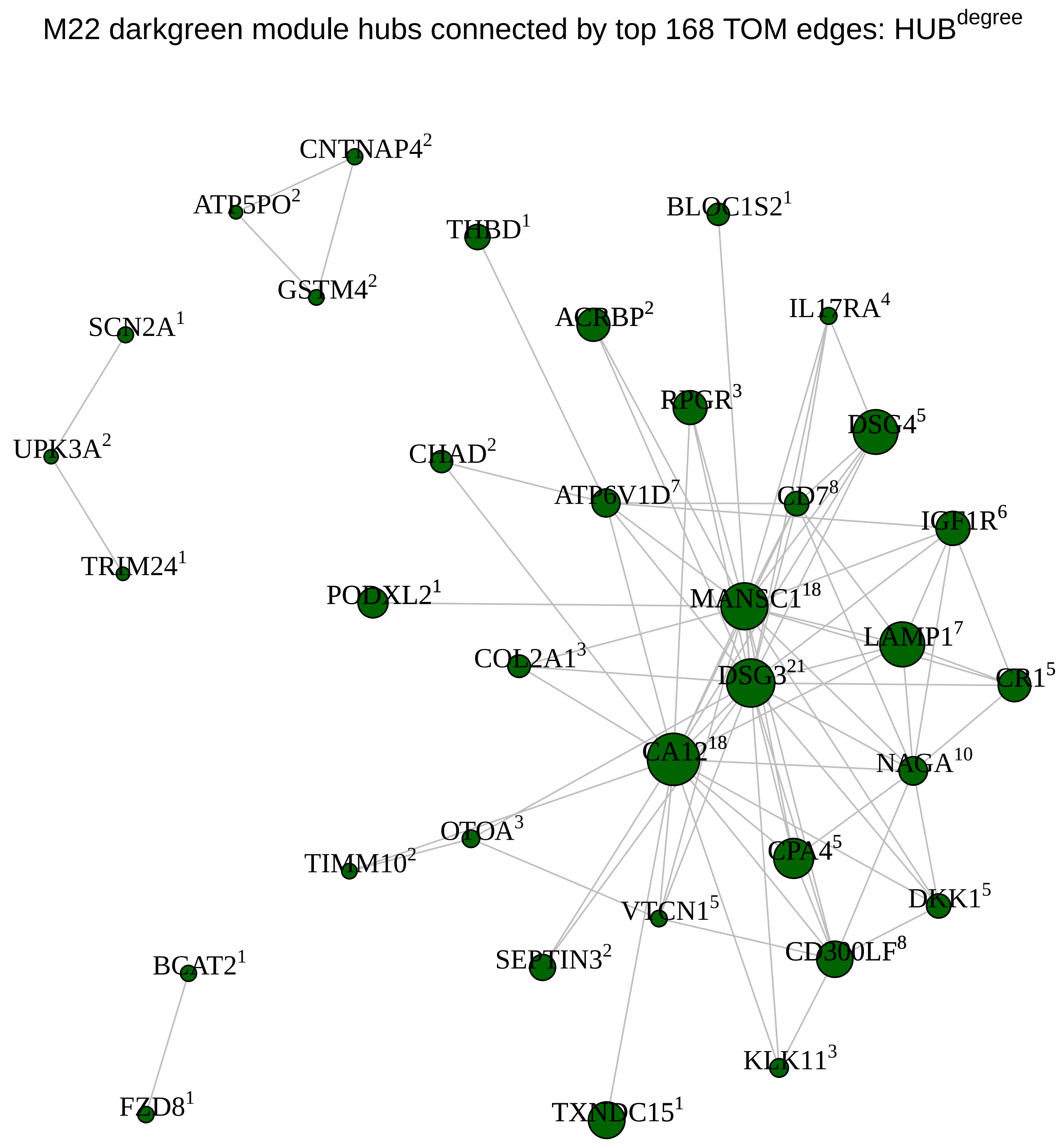
M22 darkgreen module hubs connected by top 168 TOM edges: HUB^{degree}
CNTNAP4^{2}
ATP5PO^{2}
GSTM4^{2}
THBD^{1}
BLOC1S2^{1}
SCN2A^{1}
ACRBP^{2}
IL17RA^{4}
UPK3A^{2}
RPGR^{3}
DSG4^{5}
CHAD^{2}
ATP6V1D^{7}
CD7^{8}
IGF1R^{6}
TRIM24^{1}
PODXL2^{1}
MANSC1^{18}
LAMP1^{7}
COL2A1^{3}
DSG3^{21}
CR1^{5}
CA12^{18}
NAGA^{10}
OTOA^{3}
TIMM10^{2}
CPA4^{5}
DKK1^{5}
VTCN1^{5}
BCAT2^{1}
SEPTIN3^{2}
CD300LF^{8}
KLK11^{3}
FZD8^{1}
TXNDC15^{1}

M14 cyan module

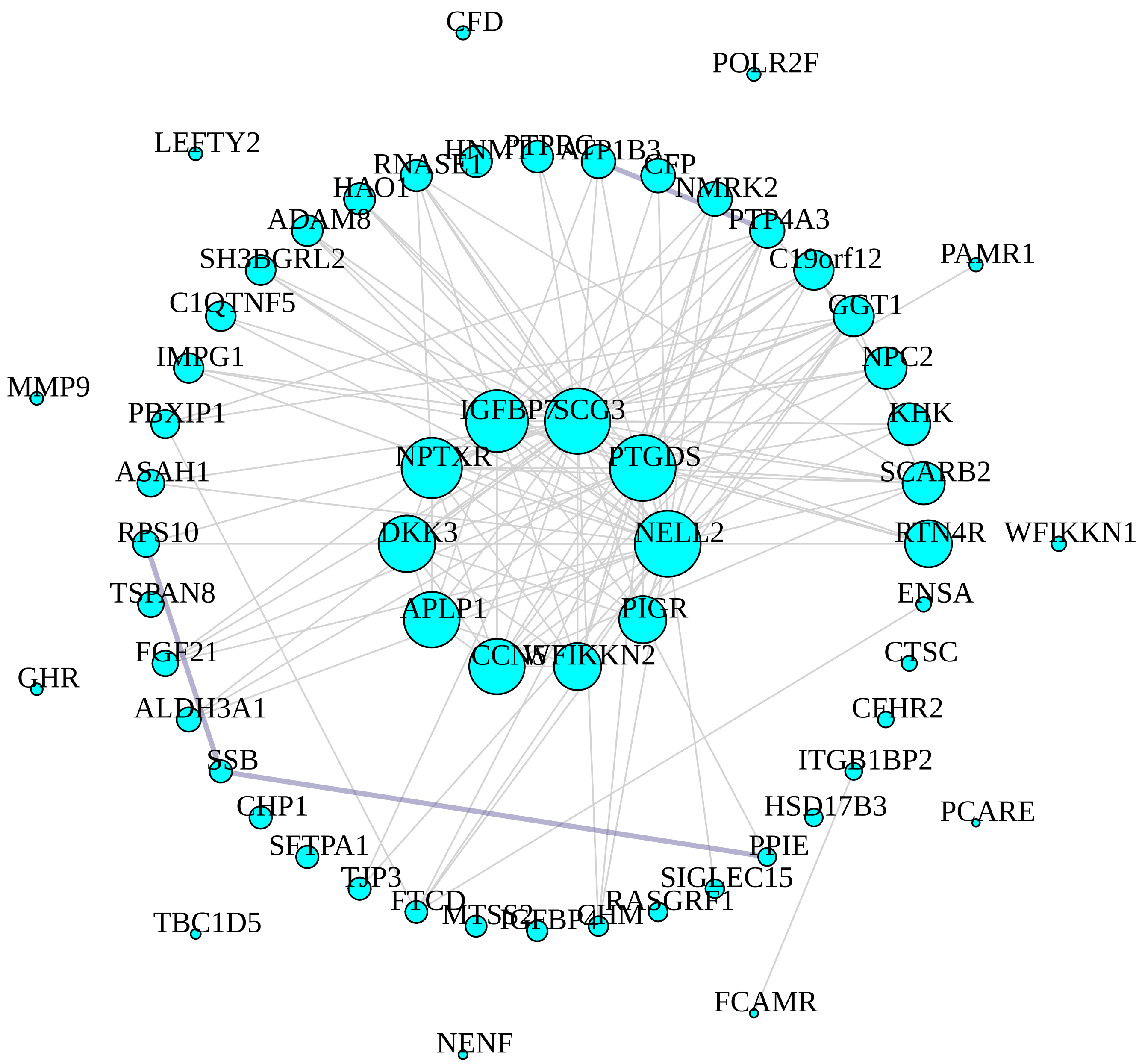

M14 cyan module hubs connected by top 280 TOM edges: HUB^{degree}

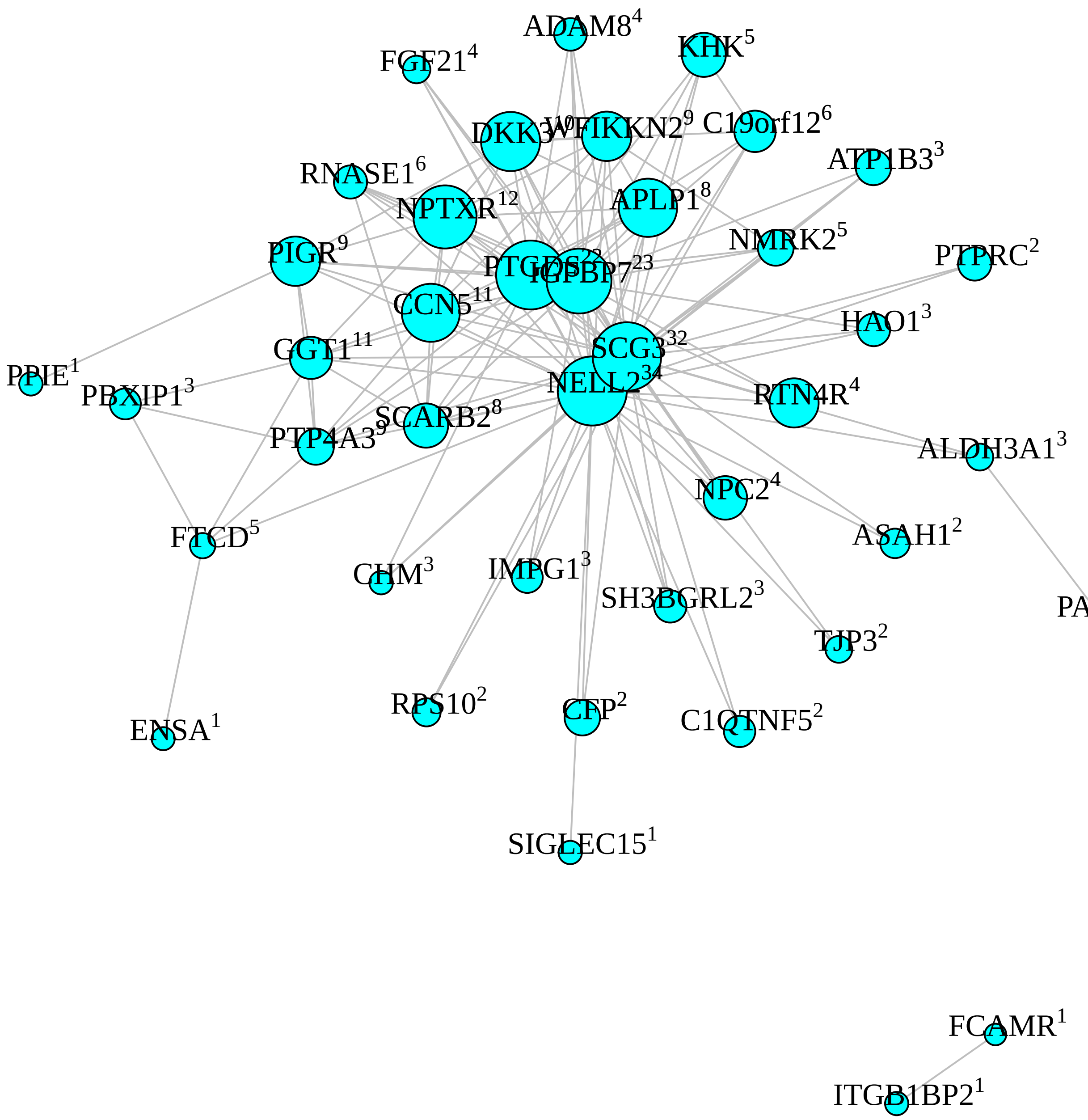

M10 purple module

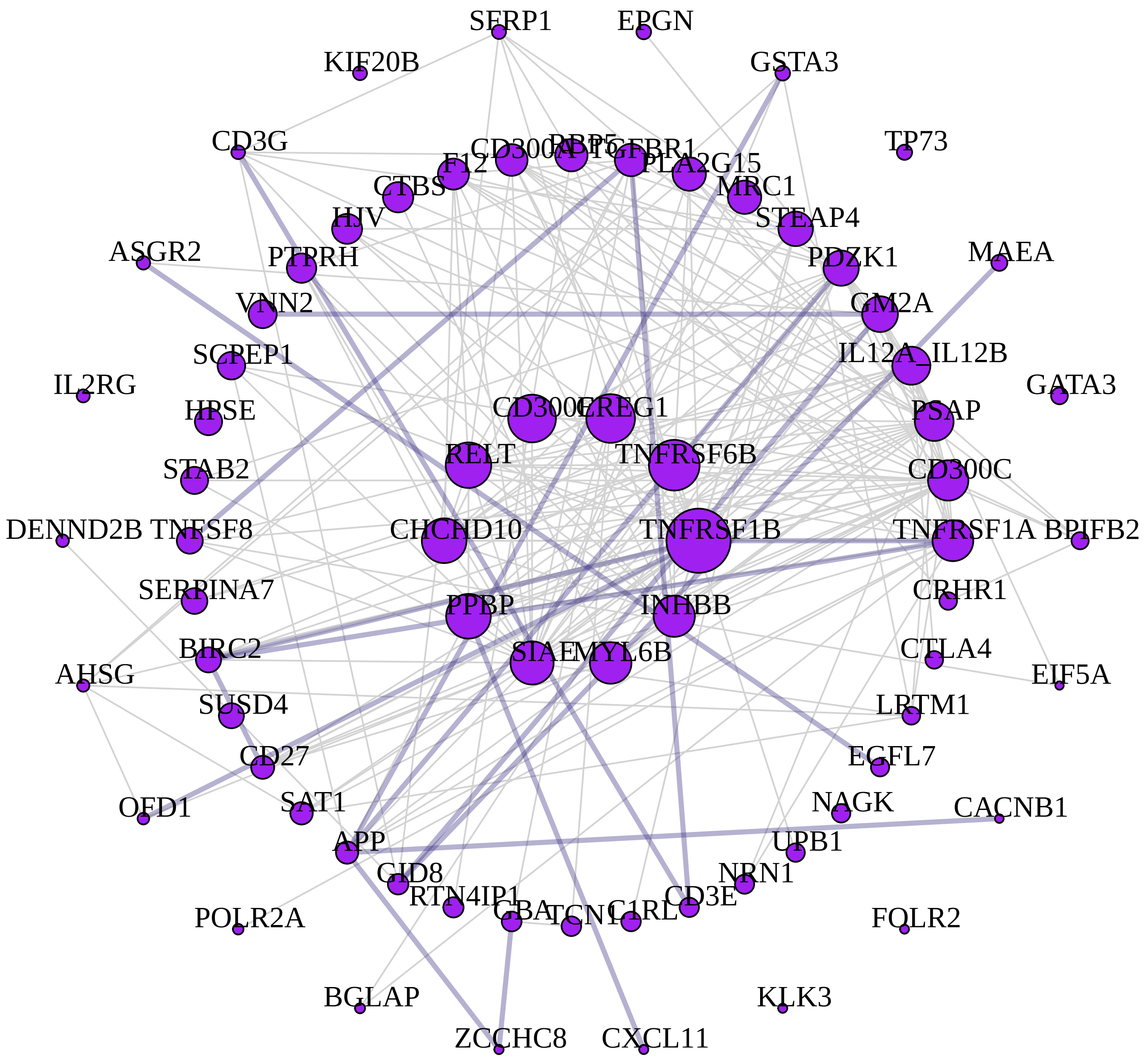

M10 purple module hubs connected by top 396 TOM edges: HUB^{degree}

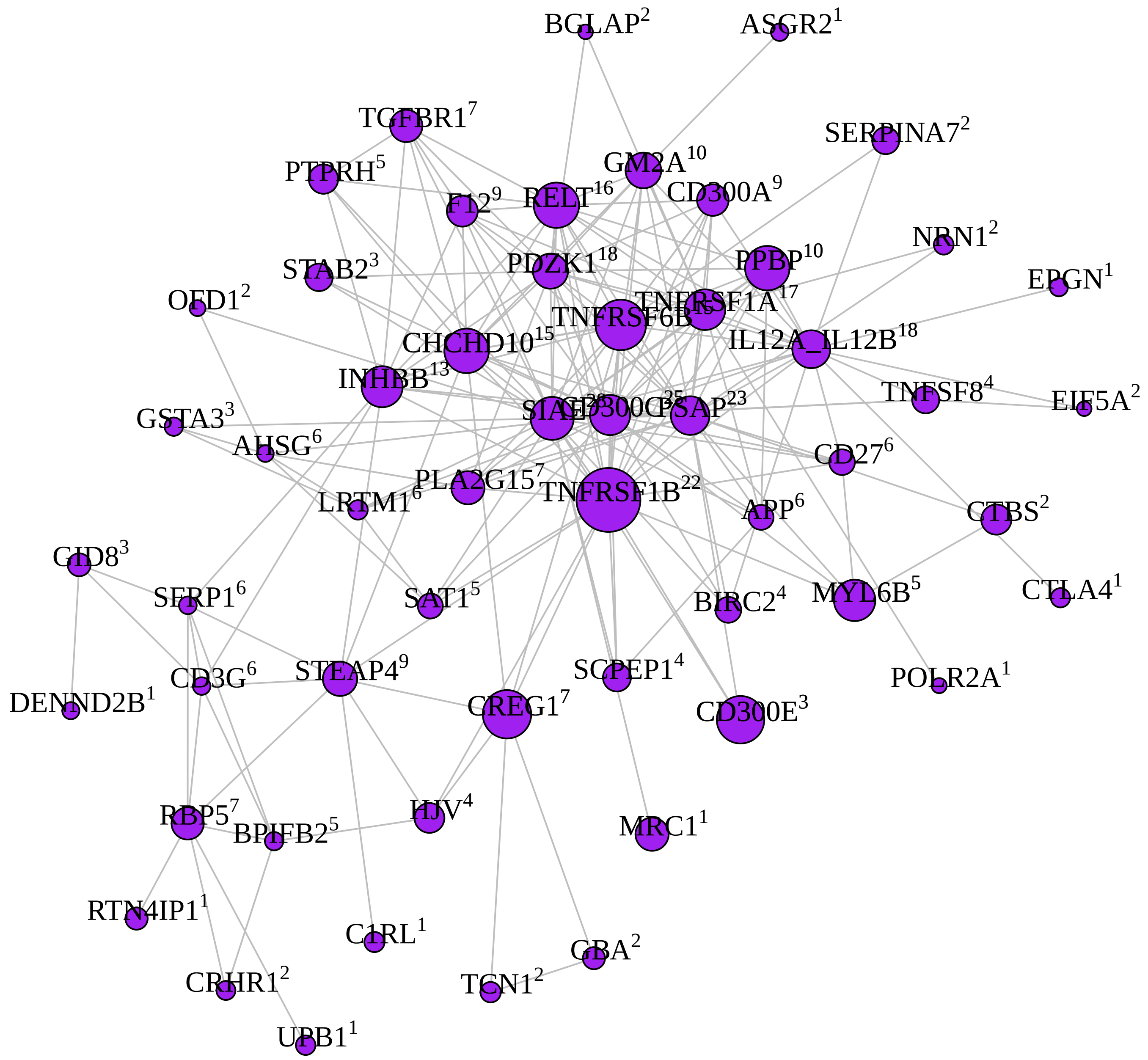

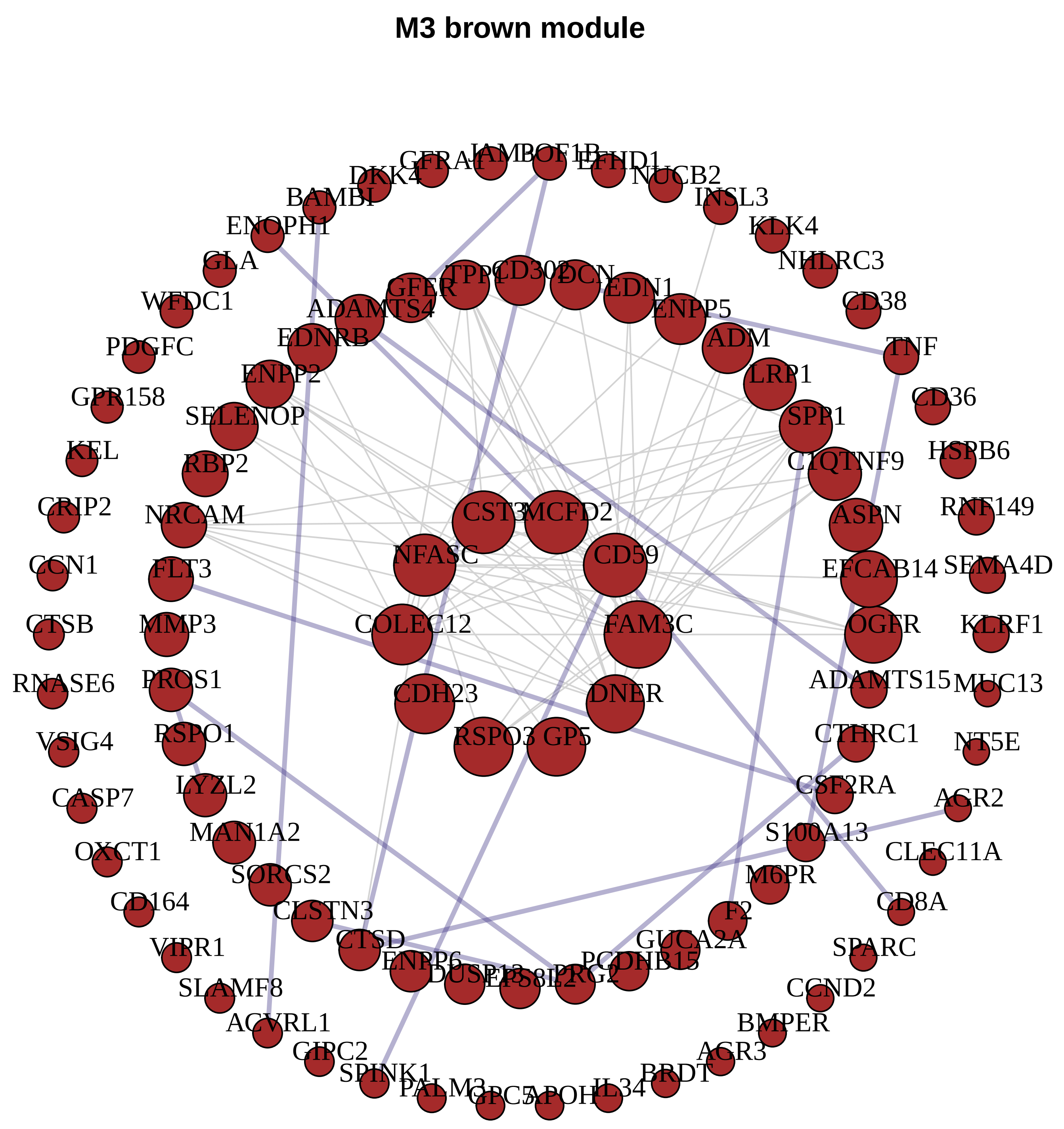
M3 brown module
DKK4
BAMBI
ENOPH1
GLA
WFDC1
PDGFC
GPR158
KEL
CRIP2
CCN1
CTSB
RNASE6
VSIG4
CASP7
OXCT1
CD164
VIPR1
SLAMF8
ACVRL1
CIPC2
SPINK1
IL34
BRDT
AGR3
BMPER
CCND2
SPARC
CD8A
CLEC11A
AGR2
NT5E
MUC13
KLRF1
SEMA4D
RNF149
HSPB6
CD36
TNF
CD38
NHLRC3
KLK4
INSL3
NUCB2
ADAMTS4
EDNRB
ENPP2
SELENOP
RBP2
NRCAM
FLT3
MMP3
PROS1
RSPO1
LYZL2
MAN1A2
SORCS2
CLSTN3
CTSD
GFER
DCN
EDN1
ENPP5
ADM
LRP1
SPP1
C1QTNF9
ASPN
EFCAB14
OGFR
ADAMTS15
CTHRC1
CSF2RA
S100A13
M6PR
F2
GUCA2A
PCDHB15
NFASC
CD59
COLEC12
FAM3C
CDH23
DNER
RSPO3
GP5
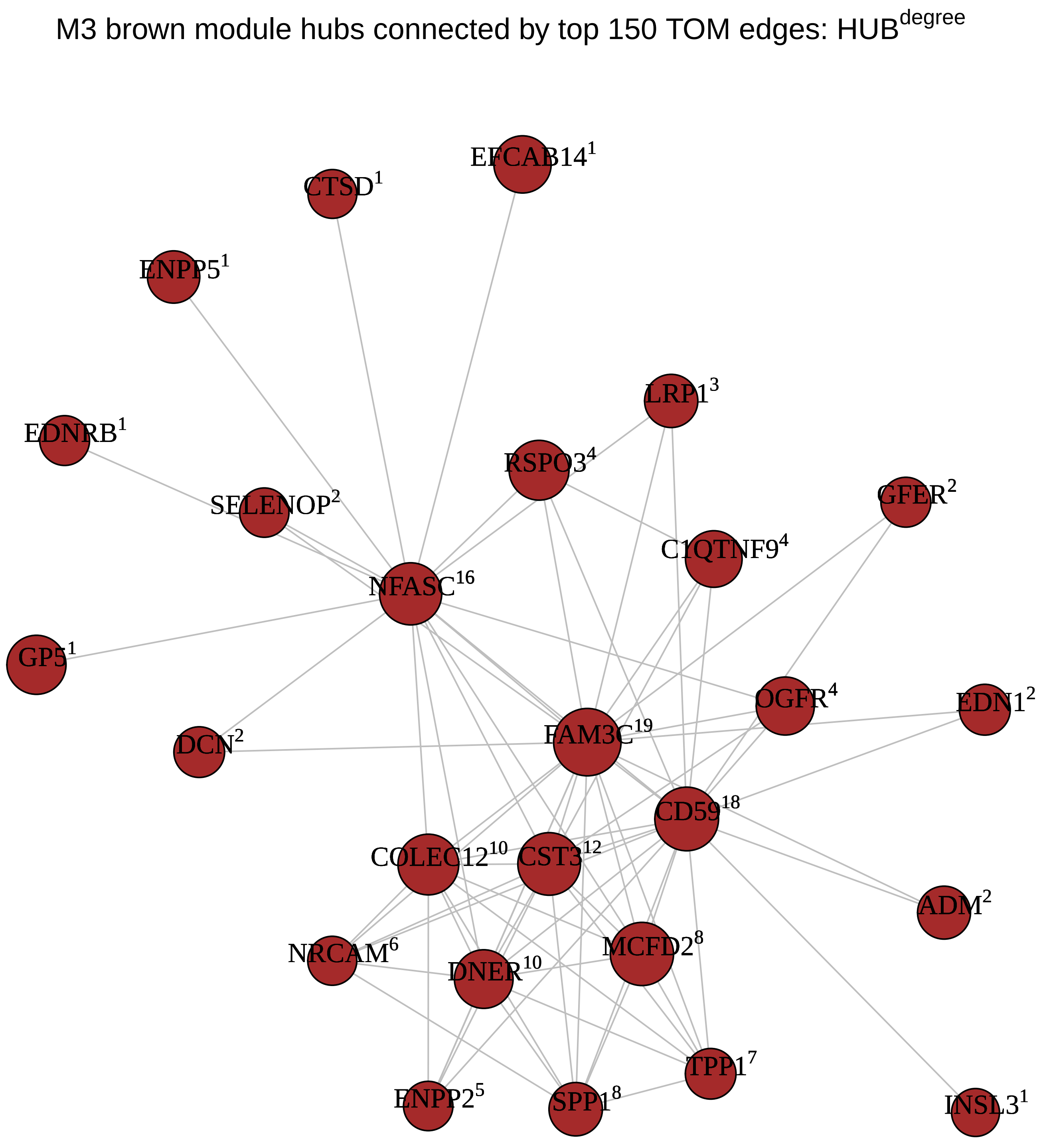
M3 brown module hubs connected by top 150 TOM edges: HUB^degree
EFCAB14^1
CTSD^1
ENPP5^1
EDNRB^1
LRP1^3
RSPO3^4
GFER^2
SELENOP^2
C1QTNF9^4
NFASC^16
GP5^1
OGFR^4
EDN1^2
DCN^2
FAM3C^19
CD59^18
ADM^2
COLEC12^10
CST3^12
NRCAM^6
DNER^10
MCFD2^8
TPP1^7
ENPP2^5
SPP1^8
INSL3^1

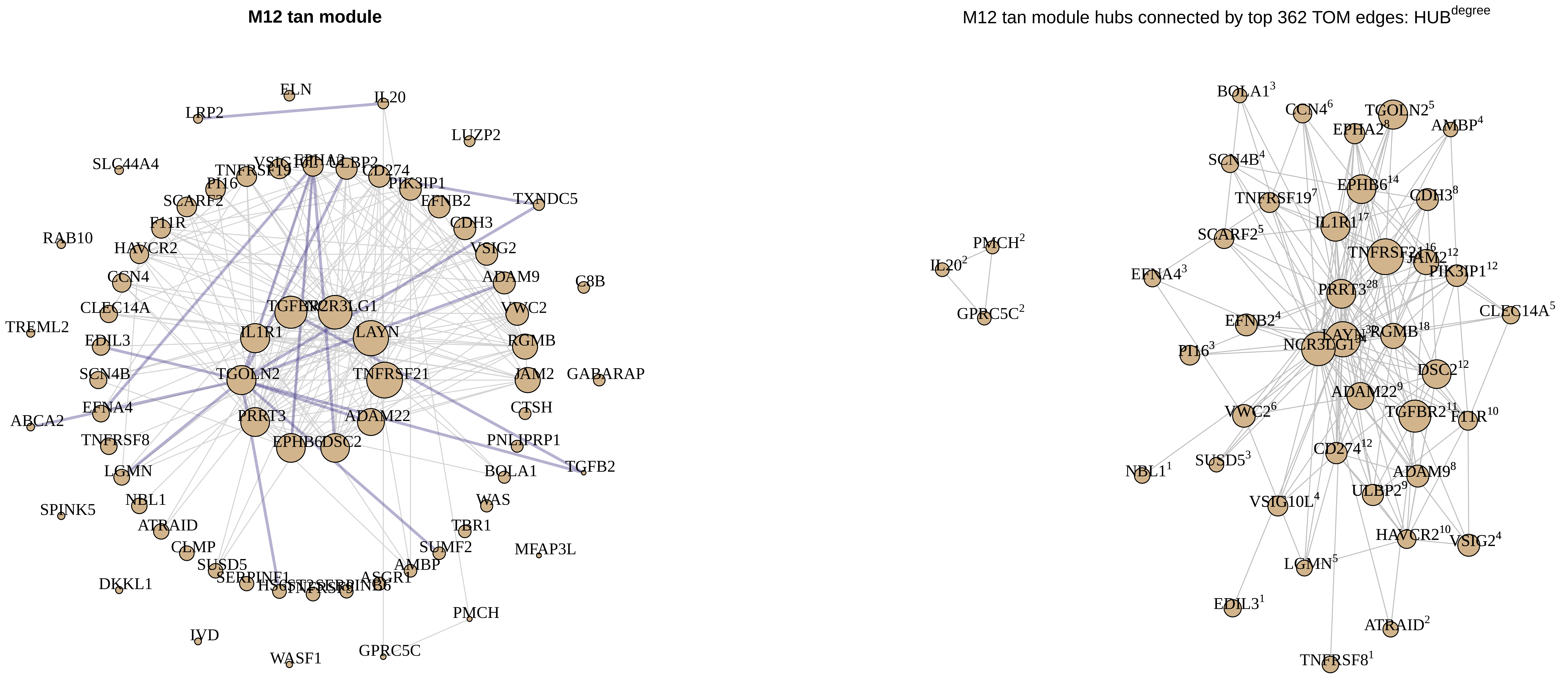
M12 tan module
ELN
IL20
LRP2
LUZP2
SLC44A4
TNFRSF19
EPHA2
ULBP2
CD274
PI16
PIK3IP1
SCARF2
EFNB2
TXNDC5
F11R
CDH3
RAB10
HAVCR2
VSIG2
CCN4
ADAM9
C8B
CLEC14A
VWC2
TREML2
IL1R1
LAYN
EDIL3
RGMB
SCN4B
TGOLN2
TNFRSF21
JAM2
GABARAP
EFNA4
CTSH
ABCA2
PRRT3
ADAM22
TNFRSF8
PNLIPRP1
EPHB6
DSC2
TGFB2
LGMN
BOLA1
SPINK5
NBL1
WAS
ATRAID
TBR1
CLMP
SUMF2
MFAP3L
SUSD5
AMBP
SERPINE1
ASGR1
DKKL1
PMCH
IVD
GPRC5C
WASF1
M12 tan module hubs connected by top 362 TOM edges: HUB^degree
BOLA1^3
CCN4^6
TGOLN2^5
EPHA2^8
AMBP^4
SCN4B^4
EPHB6^14
CDH3^8
TNFRSF19^7
IL1R1^17
SCARF2^5
PMCH^2
IL20^2
GPRC5C^2
TNFRSF21^16
JAM2^12
PIK3IP1^12
EFNA4^3
PRRT3^28
CLEC14A^5
EFNB2^4
LAYN
RGMB^18
PI16^3
NCR3LG1
DSC2^12
ADAM22^9
VWC2^6
TGFBR2^11
F11R^10
CD274^12
SUSD5^3
NBL1^1
ADAM9^8
ULBP2^9
VSIG10L^4
HAVCR2^10
VSIG2^4
LGMN^5
EDIL3^1
ATRAID^2
TNFRSF8^1

M27 white module

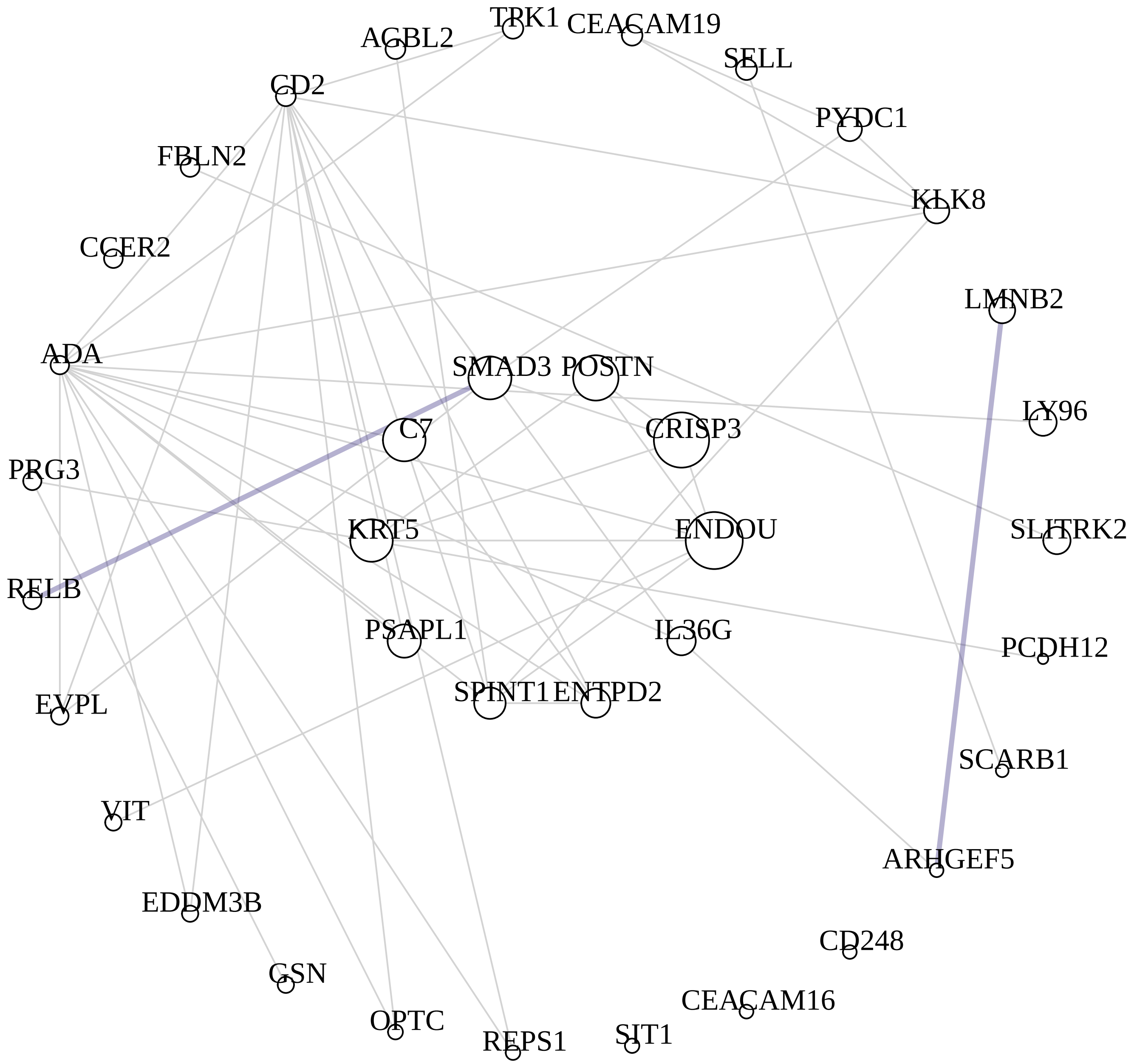

M27 white module hubs connected by top 96 TOM edges: HUB^{degree}

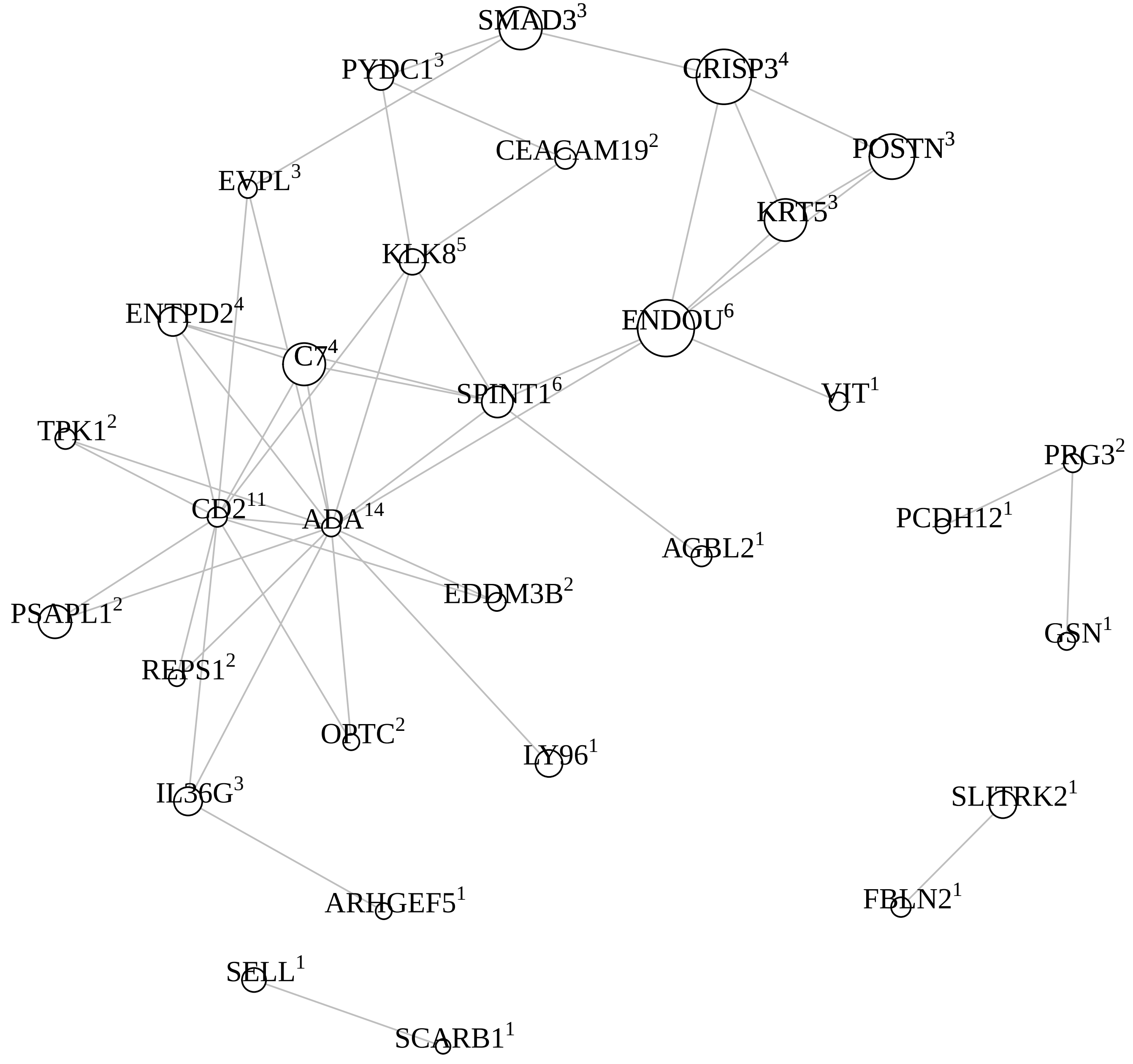

M23 darkturquoise module

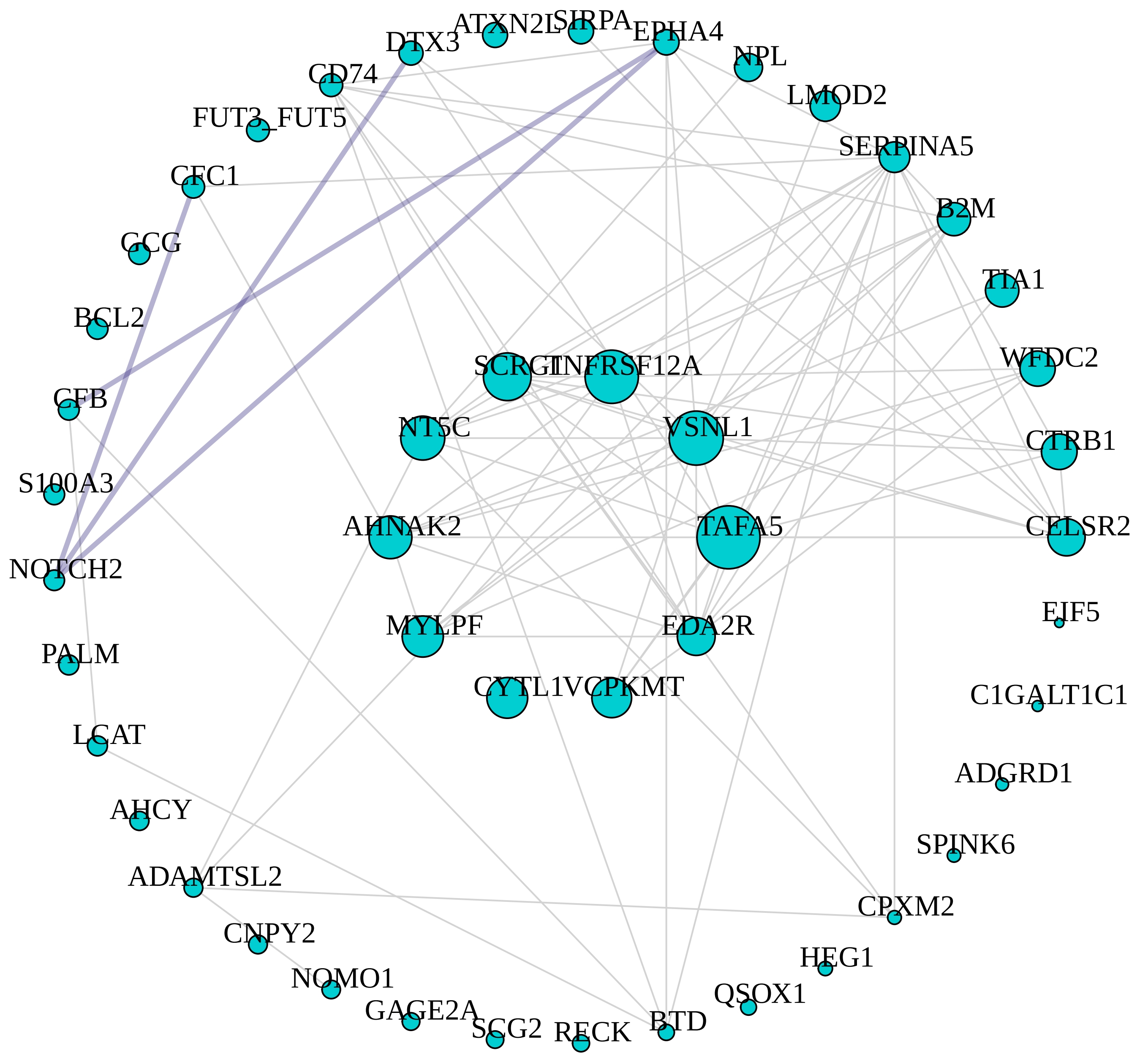

M23 darkturquoise module hubs connected by top 160 TOM edges: HUB^{degree}

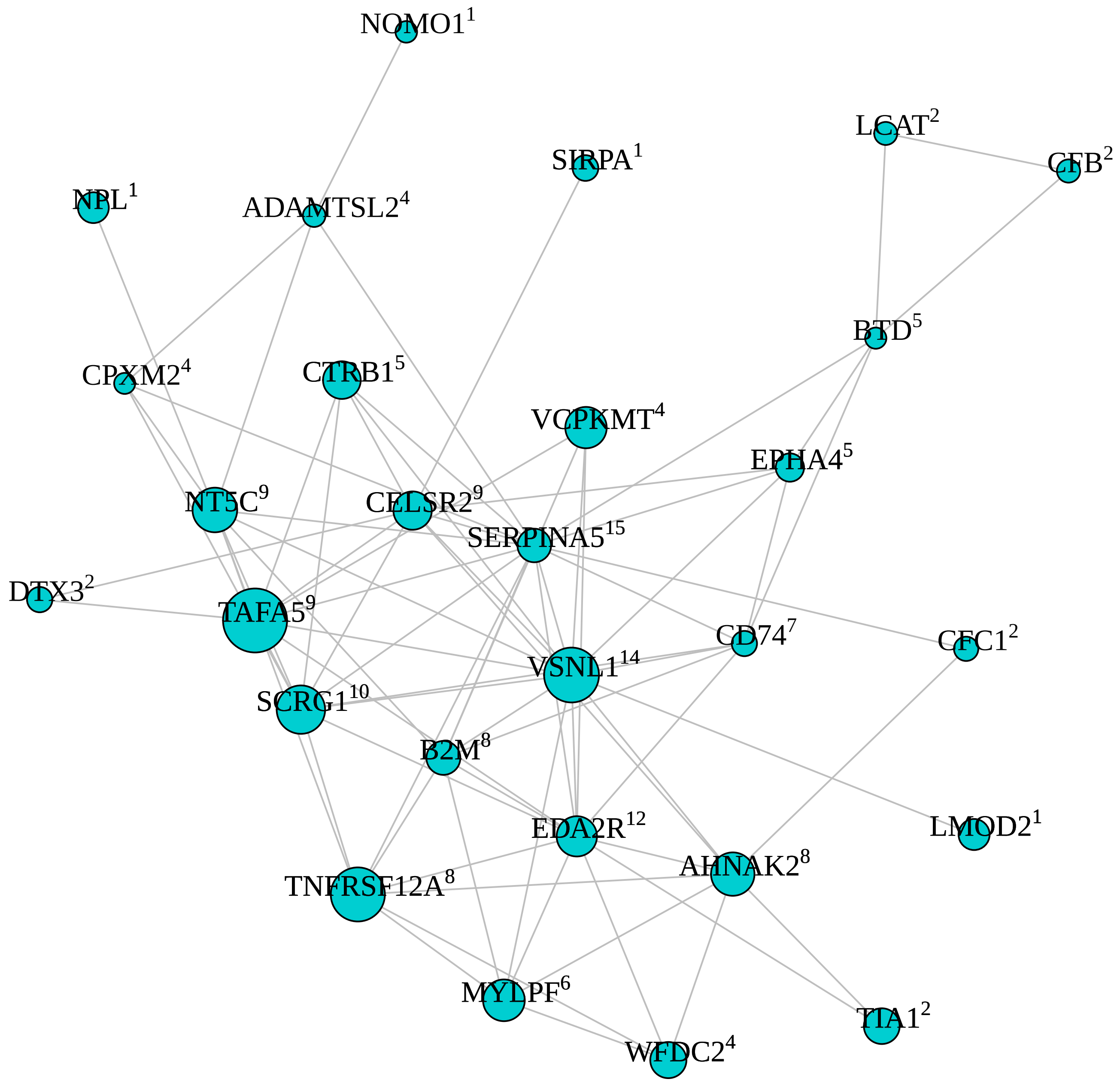

M16 lightcyan module

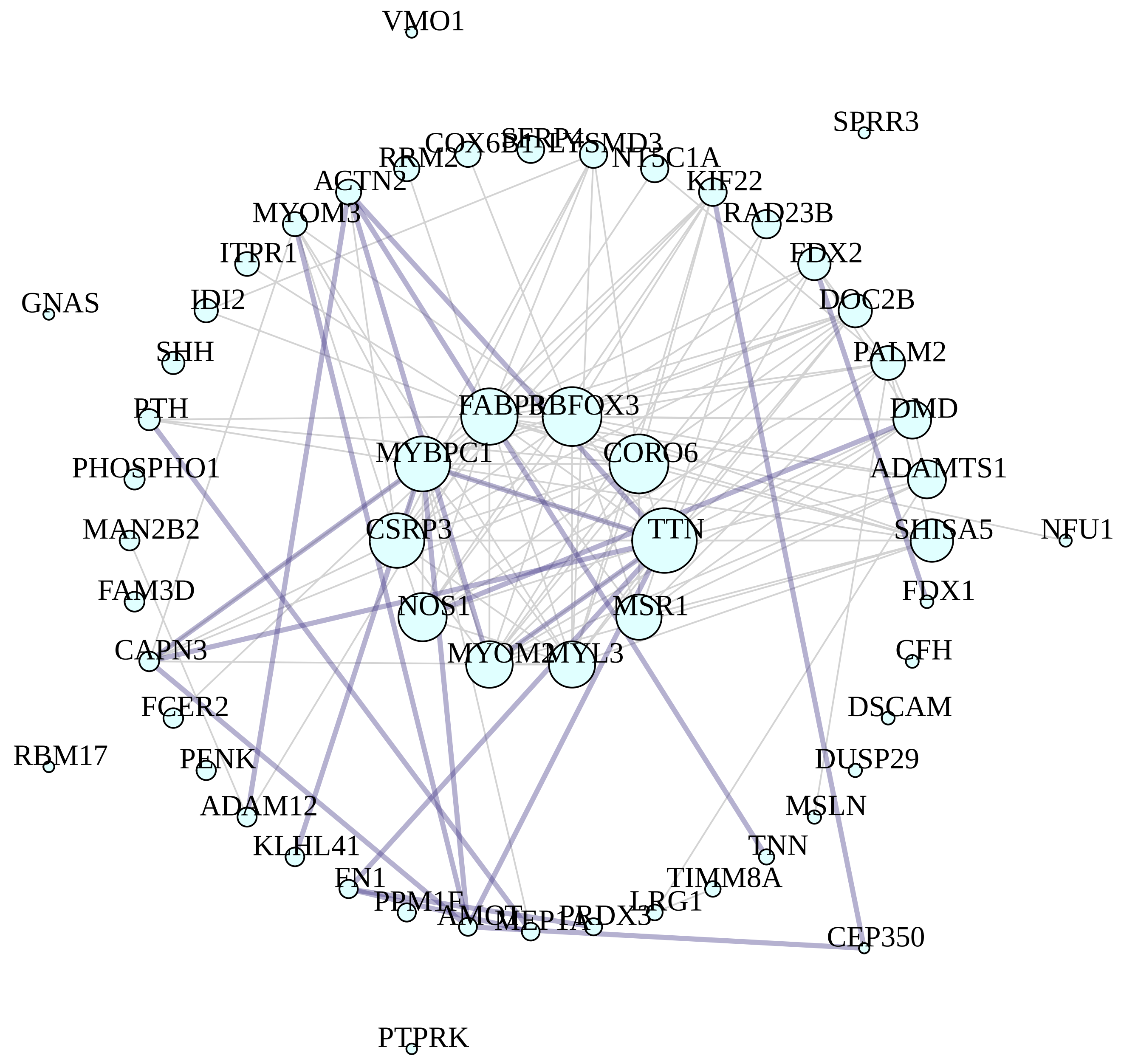

M16 lightcyan module hubs connected by top 242 TOM edges: HUB^{degree}

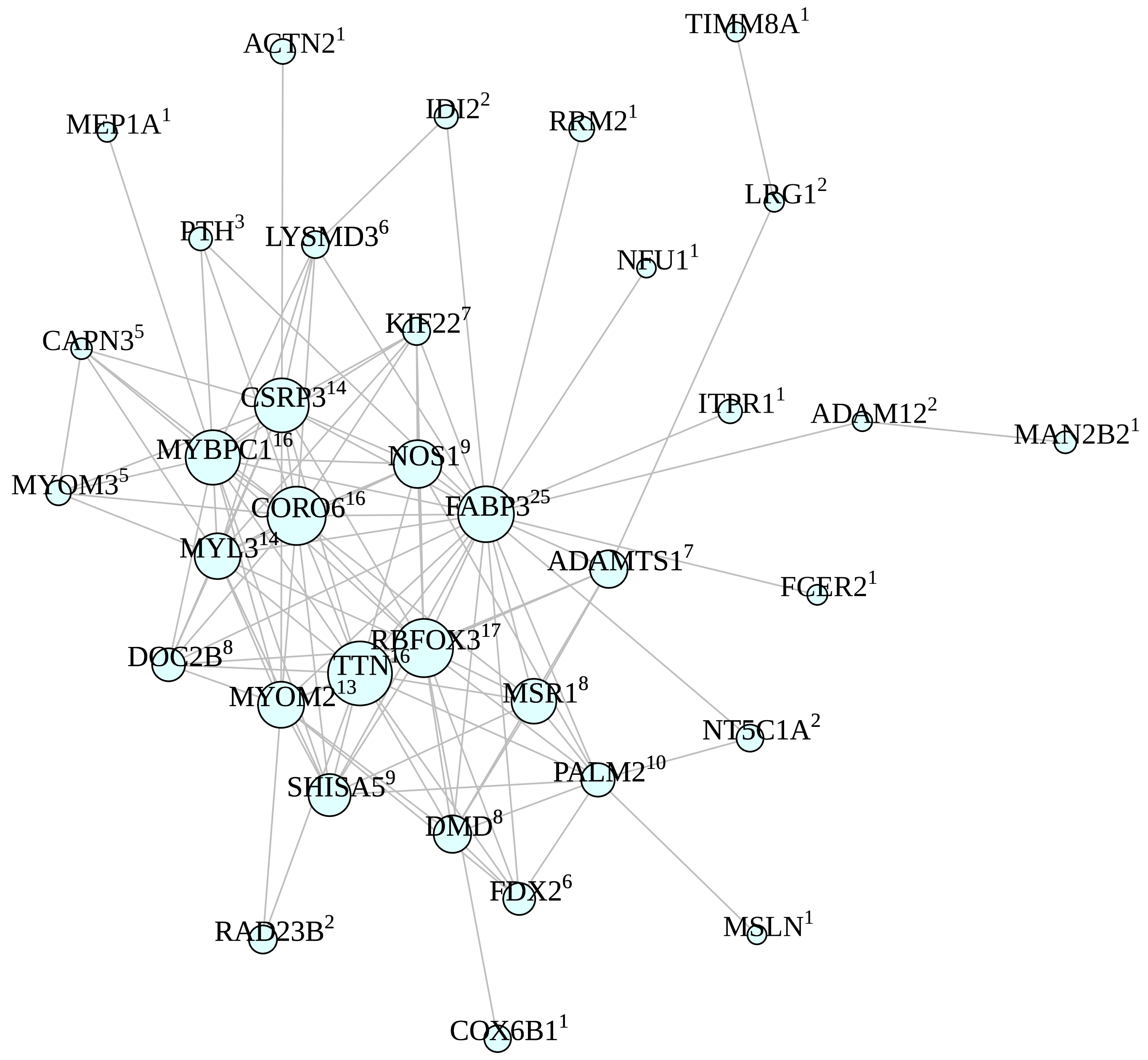

M18 lightgreen module

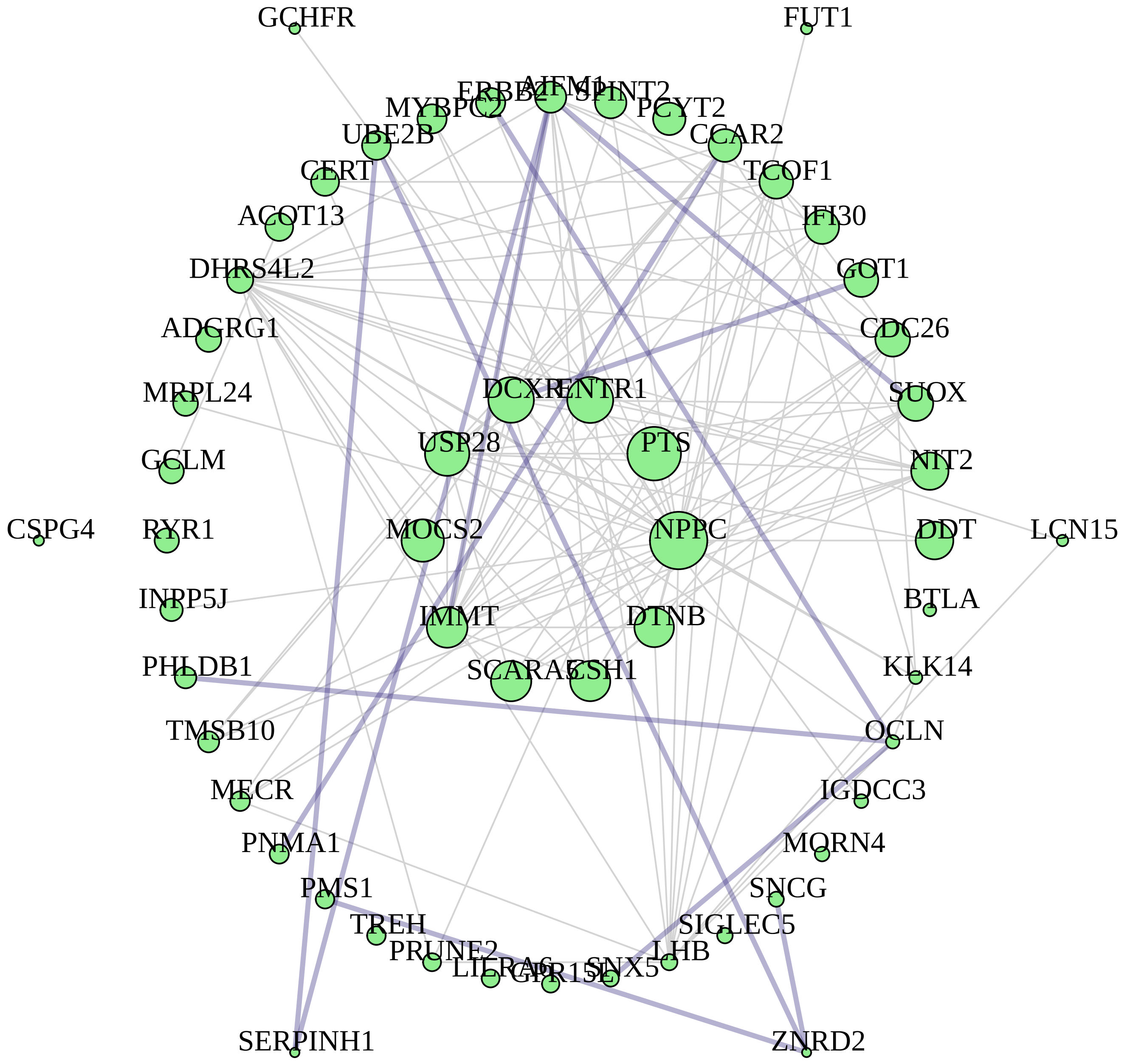

M18 lightgreen module hubs connected by top 234 TOM edges: HUB^{degree}

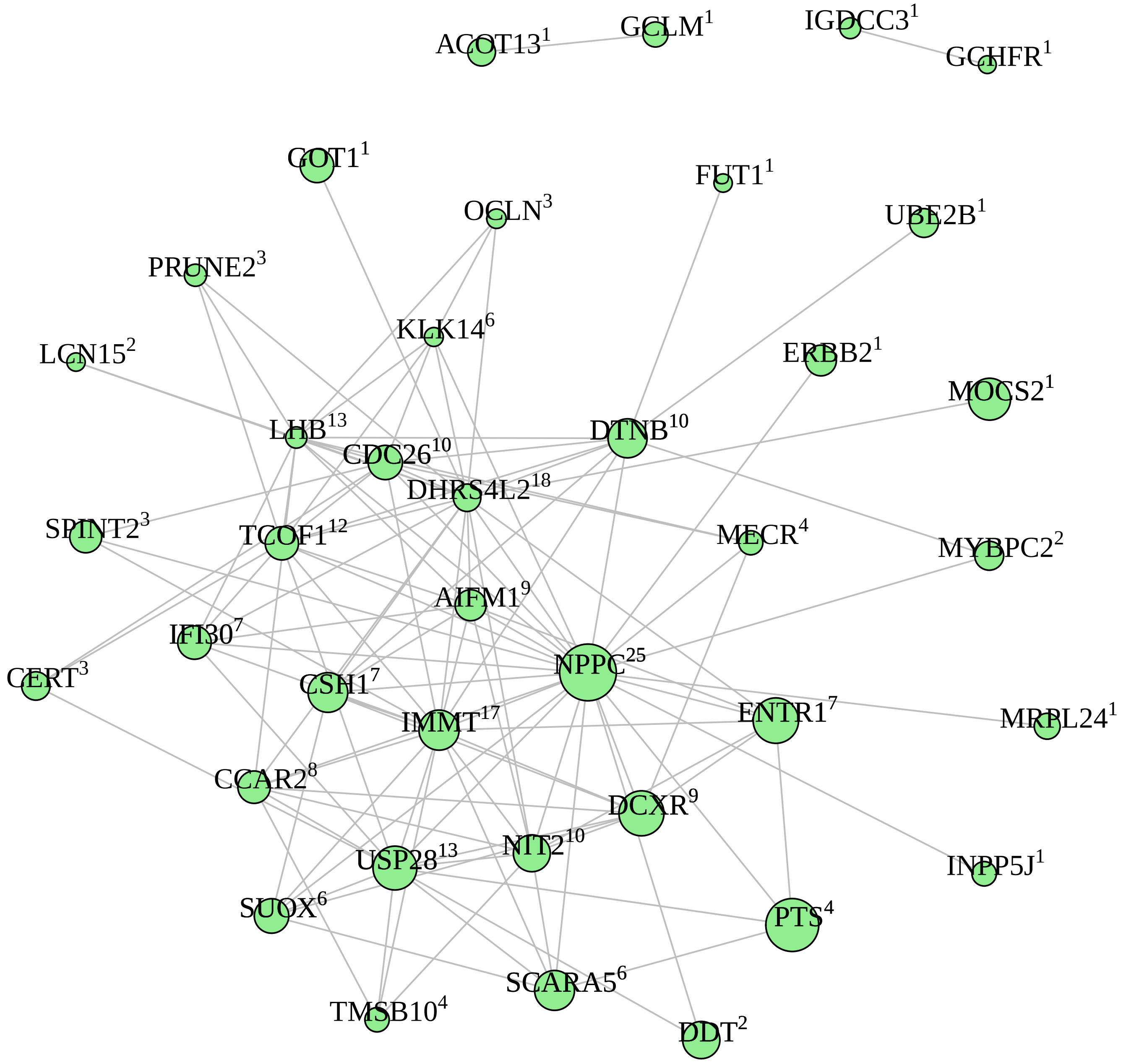

Supplement: Supplement 6 [file media-6.pdf]
