# Supplementary material for: Maintenance of chronic neuroinflammation in multiple sclerosis via interferon signaling and CD8 T cell-mediated cytotoxicity
Source: bioRxiv. 2025 Jun 11:2025.06.09.658729. Preprint. [Version 1] doi: 10.1101/2025.06.09.658729 (PMC12190403; doi:10.1101/2025.06.09.658729)

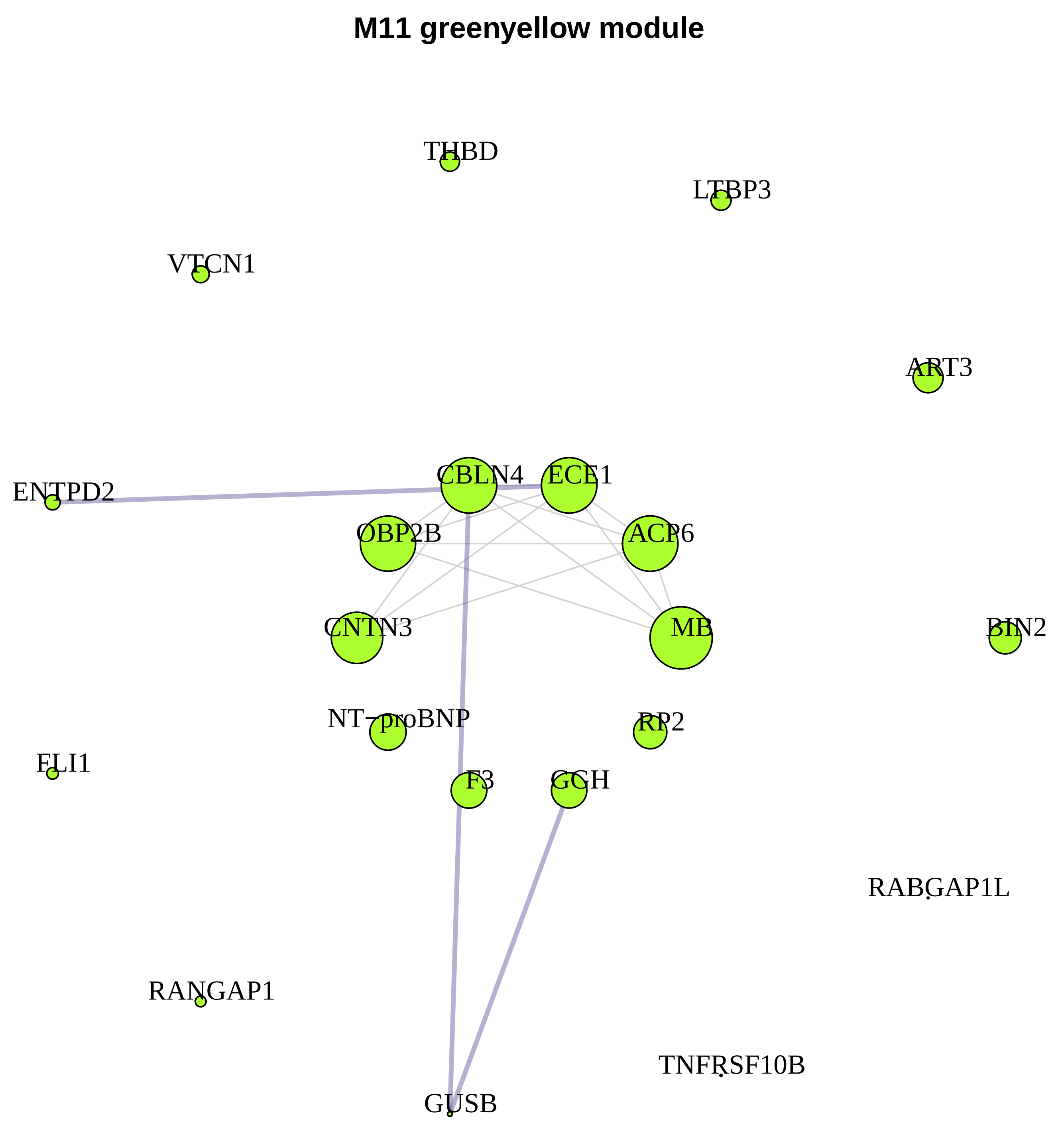

M11 greenyellow module
THBD
LTBP3
VTCN1
ART3
ENTPD2
CBLN4
ECE1
OBP2B
ACP6
CNTN3
MB
BIN2
NT-proBNP
RP2
FLI1
F3
GCH
RABGAP1L
RANGAP1
TNFRSF10B
GUSB


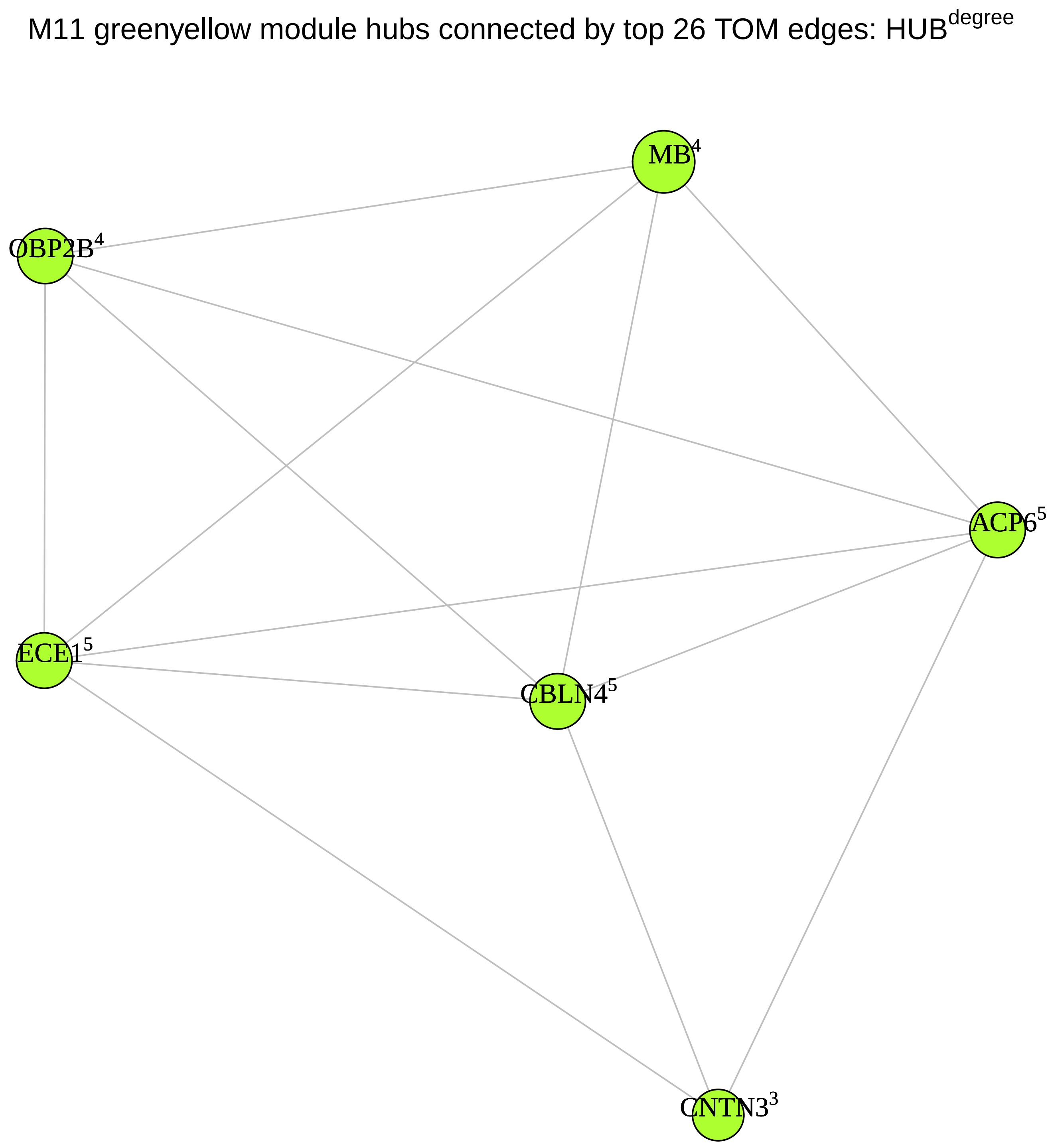

M11 greenyellow module hubs connected by top 26 TOM edges: HUB^degree
MB^4
OBP2B^4
ACP6^5
ECE1^5
CBLN4^5
CNTN3^3

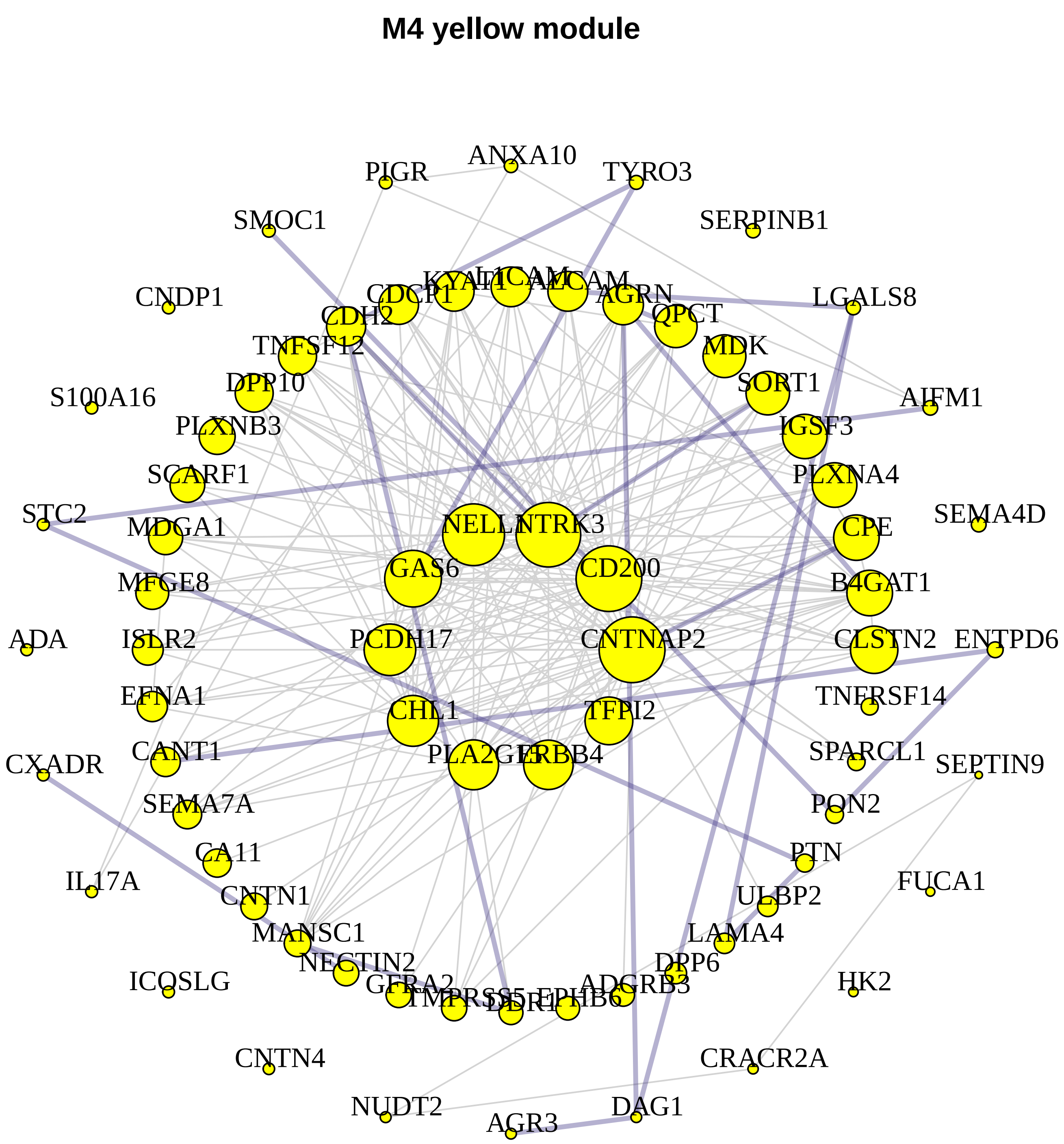
M4 yellow module
PIGR
ANXA10
TYRO3
SMOC1
SERPINB1
CNDP1
CDCP1
AGRN
LGALS8
CDH2
QPCT
TNFSF12
MDK
DPP10
SORT1
S100A16
AIFM1
PLXNB3
IGSF3
SCARF1
PLXNA4
STC2
SEMA4D
MDGA1
CPE
GAS6
CD200
MFGE8
B4GAT1
ADA
ISLR2
PCDH17
CNTNAP2
CLSTN2
ENTPD6
EFNA1
CHL1
TFPI2
TNFRSF14
CANT1
SPARCL1
CXADR
SEPTIN9
SEMA7A
PON2
CA11
PTN
IL17A
CNTN1
FUCA1
ULBP2
MANSC1
LAMA4
NECTIN2
DPP6
ICOSLG
GFRA2
ADGRB3
HK2
CNTN4
CRACR2A
NUDT2
DAG1
AGR3

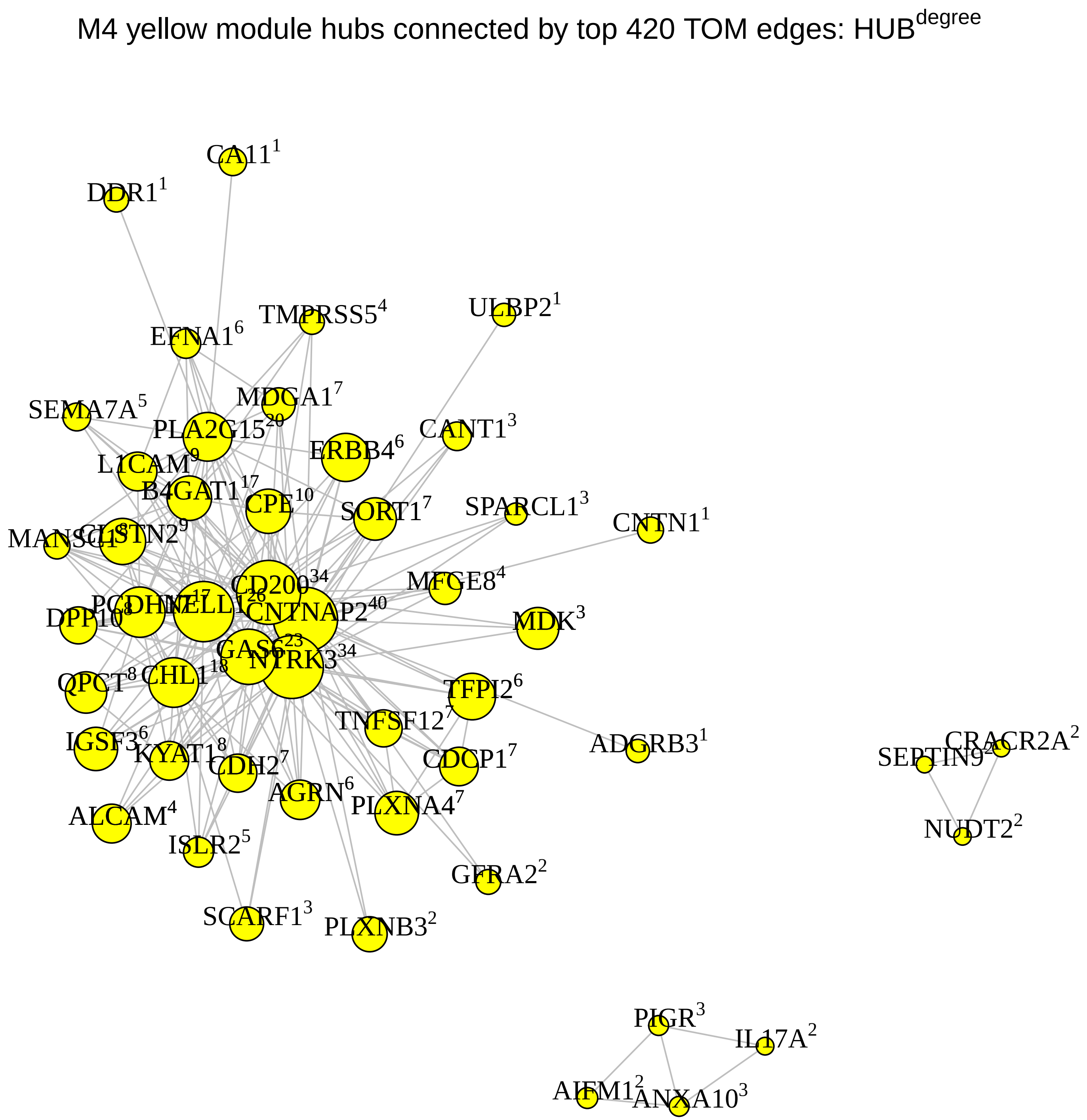
M4 yellow module hubs connected by top 420 TOM edges: HUB^degree
CA11
DDR1
EFNA1
TMPRSS5
ULBP2
SEMA7A
MDGA1
PLA2G15
CANT1
ERBB4
L1CAM
B4GAT1
CPE
SORT1
SPARCL1
CNTN1
MFGE8
CD200
CNTNAP2
MDK
DPP10
GAS6
NTRK3
QPCT
CHL1
TFPI2
TNFSF12
IGSF3
KYAT1
CDH2
CDCP1
ADGRB3
SEPTIN9
CRACR2A
NUDT2
AGRN
PLXNA4
ALCAM
ISLR2
GFRA2
SCARF1
PLXNB3
PIGR
IL17A
AIFM1
ANXA10

**M7 black module**

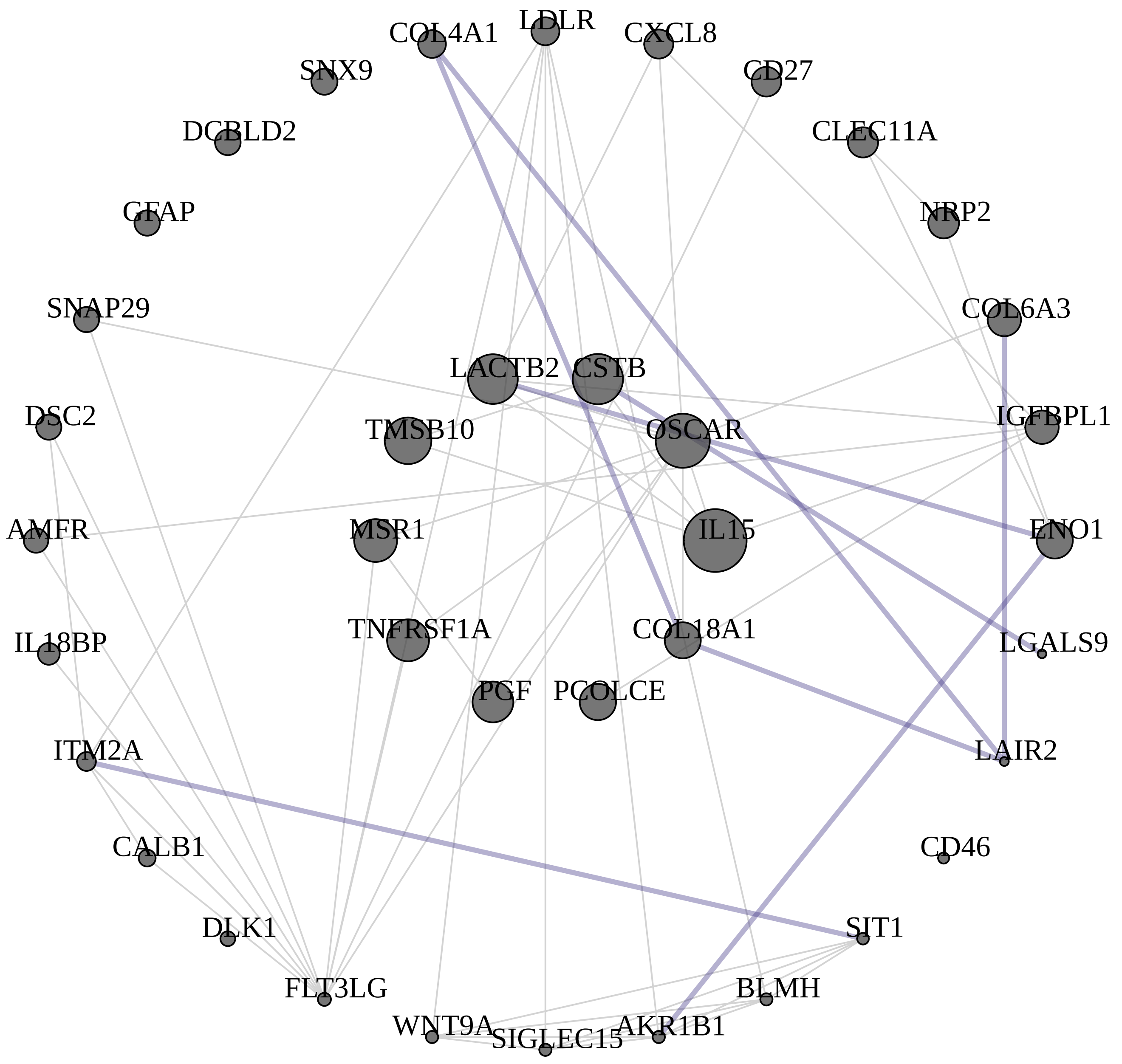


M7 black module hubs connected by top 102 TOM edges: HUB$^{degree}$

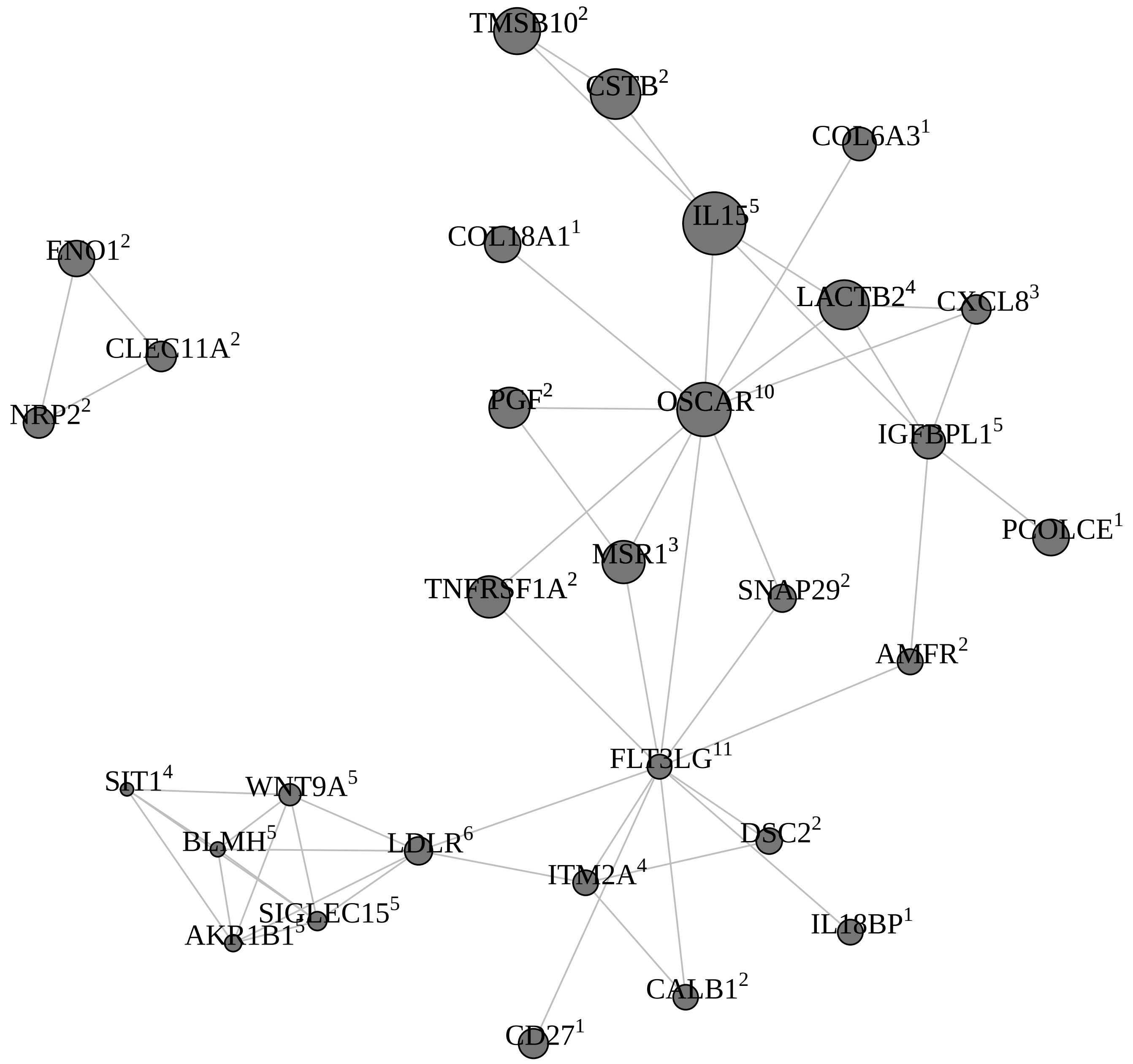

**M2 blue module**

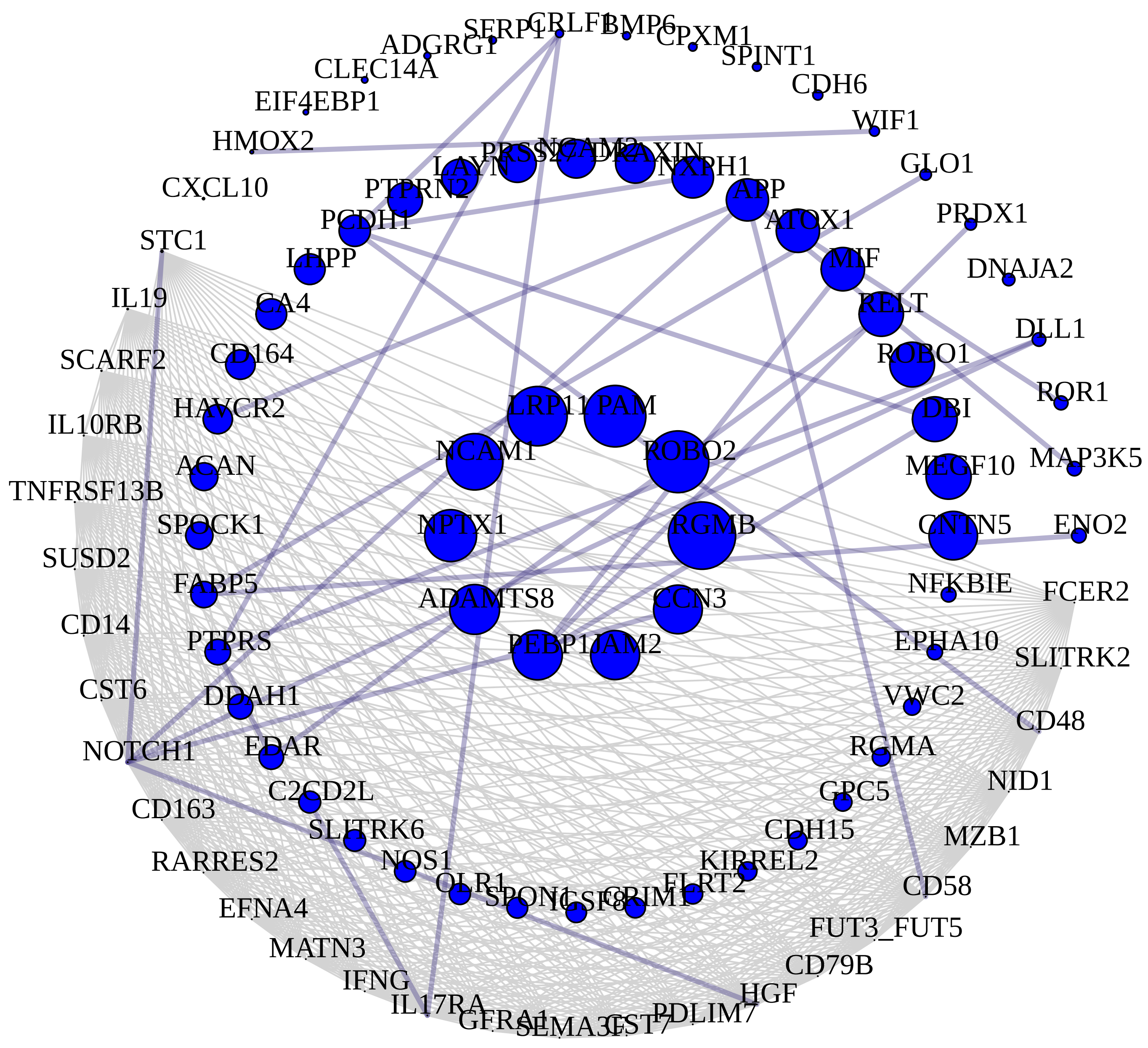


M2 blue module hubs connected by top 736 TOM edges: HUB$^{degree}$

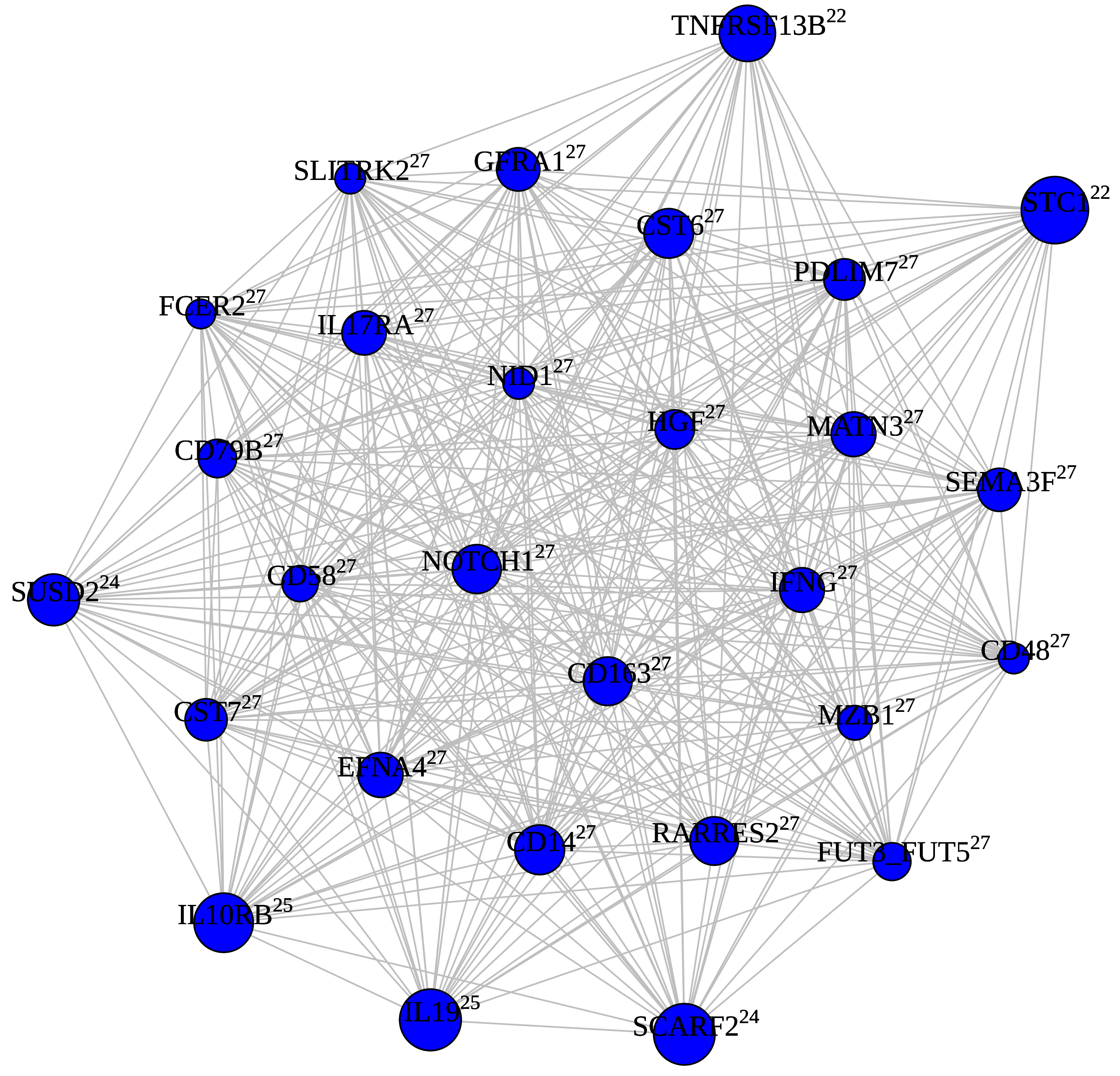

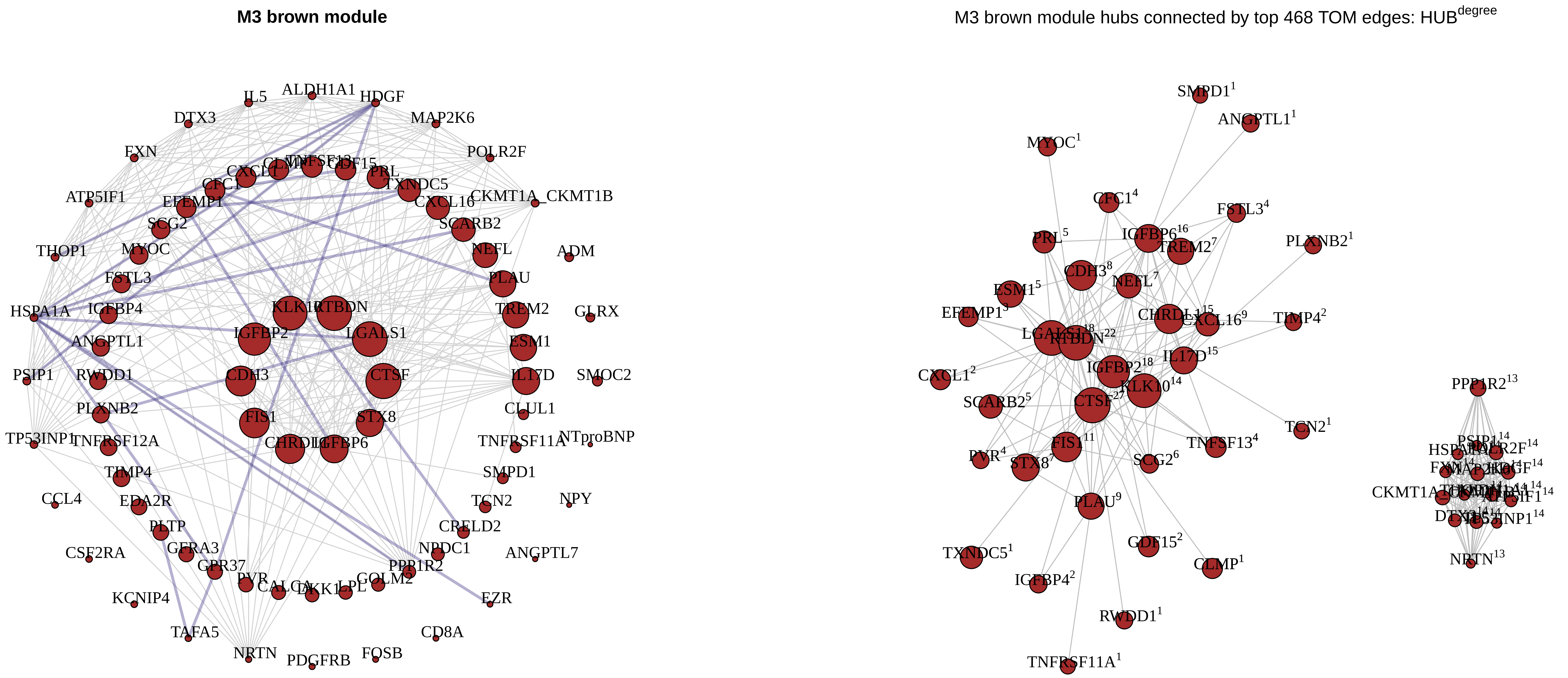
M3 brown module
M3 brown module hubs connected by top 468 TOM edges: HUB^degree
IL5
ALDH1A1
HDGF
DTX3
MAP2K6
FXN
POLR2F
ATP5IF1
CKMT1A_CKMT1B
THOP1
ADM
HSPA1A
GLRX
PSIP1
SMOC2
TP53INP1
NTproBNP
CCL4
NPY
CSF2RA
ANGPTL7
KCNIP4
EZR
TAFA5
CD8A
NRTN
PDGFRB
FOSB
CXCL1
CFC1
EFEMP1
SCG2
MYOC
FSTL3
IGFBP4
ANGPTL1
RWDD1
PLXNB2
TNFRSF12A
TIMP4
EDA2R
PLTP
GFRA3
GPR37
PVR
CALCA
LPL
GOLM2
PPP1R2
NPDC1
CRELD2
TCN2
SMPD1
TNFRSF11A
CLUL1
IL17D
ESM1
TREM2
PLAU
NEFL
SCARB2
CXCL16
TXNDC5
PRL
GDF15
KLK10
RTBDN
IGFBP2
LGALS1
CDH3
CTSF
FIS1
STX8
CHRDL1
IGFBP6
SMPD1 1
ANGPTL1 1
MYOC 1
CFC1 4
FSTL3 4
PLXNB2 1
PRL 5
IGFBP6 16
TREM2 7
CDH3 8
NEFL 7
ESM1 5
EFEMP1 3
CHRDL1 15
CXCL16 9
TIMP4 2
IL17D 15
IGFBP2 18
CXCL1 2
KLK10 14
SCARB2 5
CTSF 27
TCN2 1
PPP1R2 13
FIS1 11
TNFSF13 4
PVR 4
STX8 7
SCG2 6
CKMT1A
PLAU 9
TXNDC5 1
GDF15 2
CLMP 1
IGFBP4 2
NRTN 13
RWDD1 1
TNFRSF11A 1

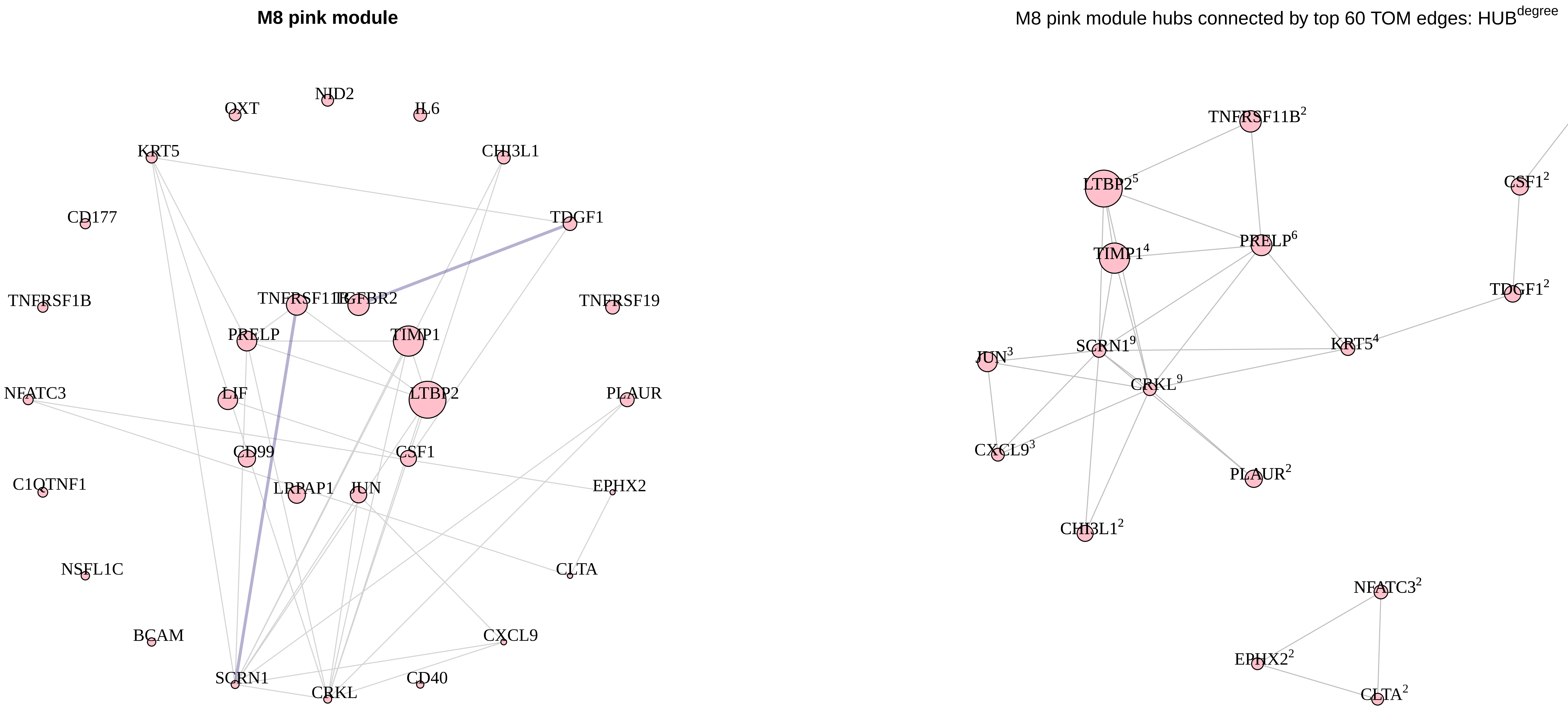

M8 pink module
OXT
NID2
IL6
KRT5
CHI3L1
CD177
TDGF1
TNFRSF1B
TNFRSF11B
TGFBR2
TNFRSF19
PRELP
TIMP1
NFATC3
LIF
LTBP2
PLAUR
CD99
CSF1
C1QTNF1
LRPAP1
JUN
EPHX2
NSFL1C
CLTA
BCAM
CXCL9
SCRN1
CRKL
CD40
M8 pink module hubs connected by top 60 TOM edges: HUB$^{degree}$
LIF$^{1}$
TNFRSF11B$^{2}$
LTBP2$^{5}$
CSF1$^{2}$
PRELP$^{6}$
TIMP1$^{4}$
TDGF1$^{2}$
SCRN1$^{9}$
KRT5$^{4}$
JUN$^{3}$
CRKL$^{9}$
CXCL9$^{3}$
PLAUR$^{2}$
CHI3L1$^{2}$
NFATC3$^{2}$
EPHX2$^{2}$
CLTA$^{2}$

**M5 green module**

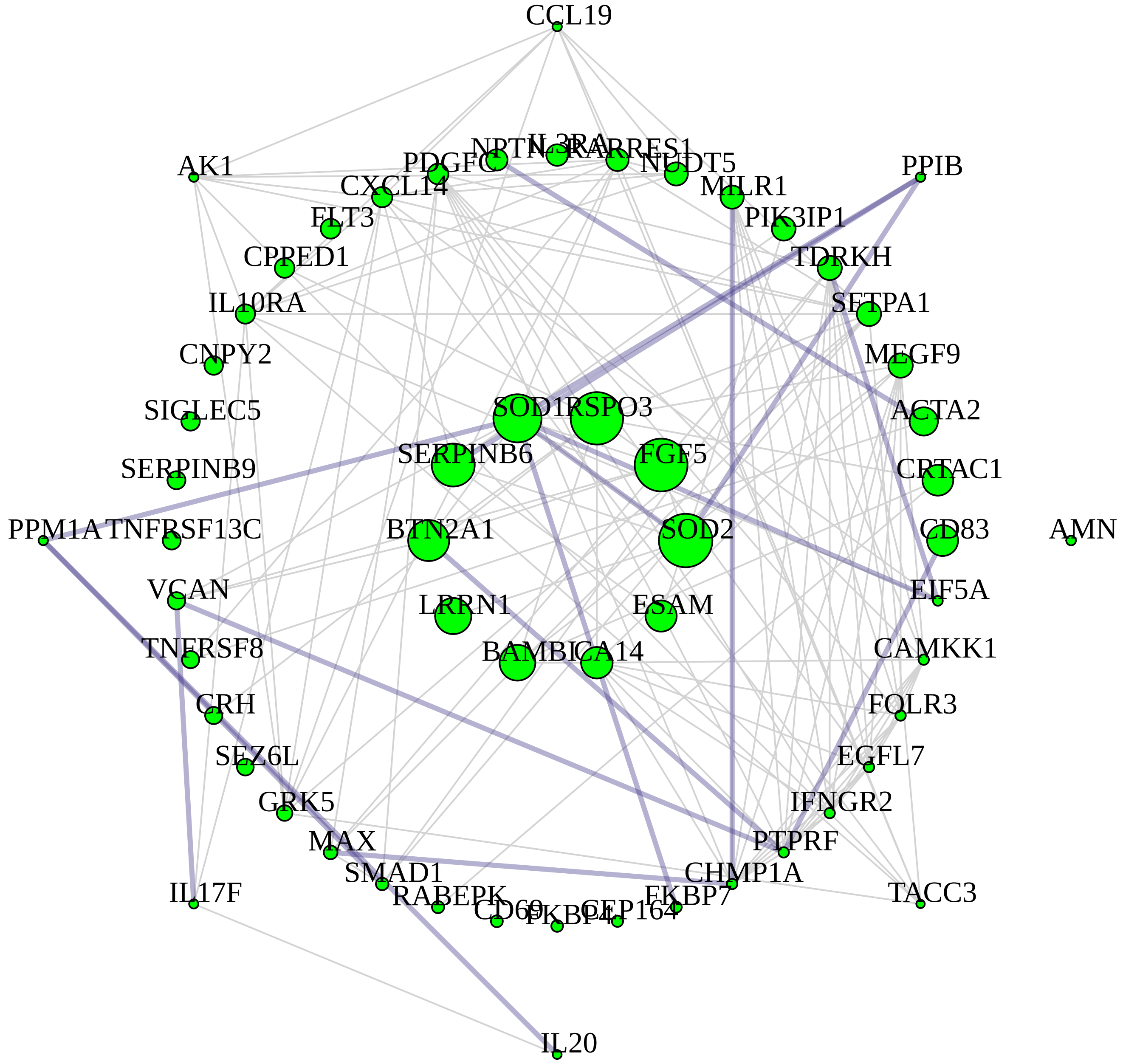


M5 green module hubs connected by top 252 TOM edges: HUB$^{degree}$

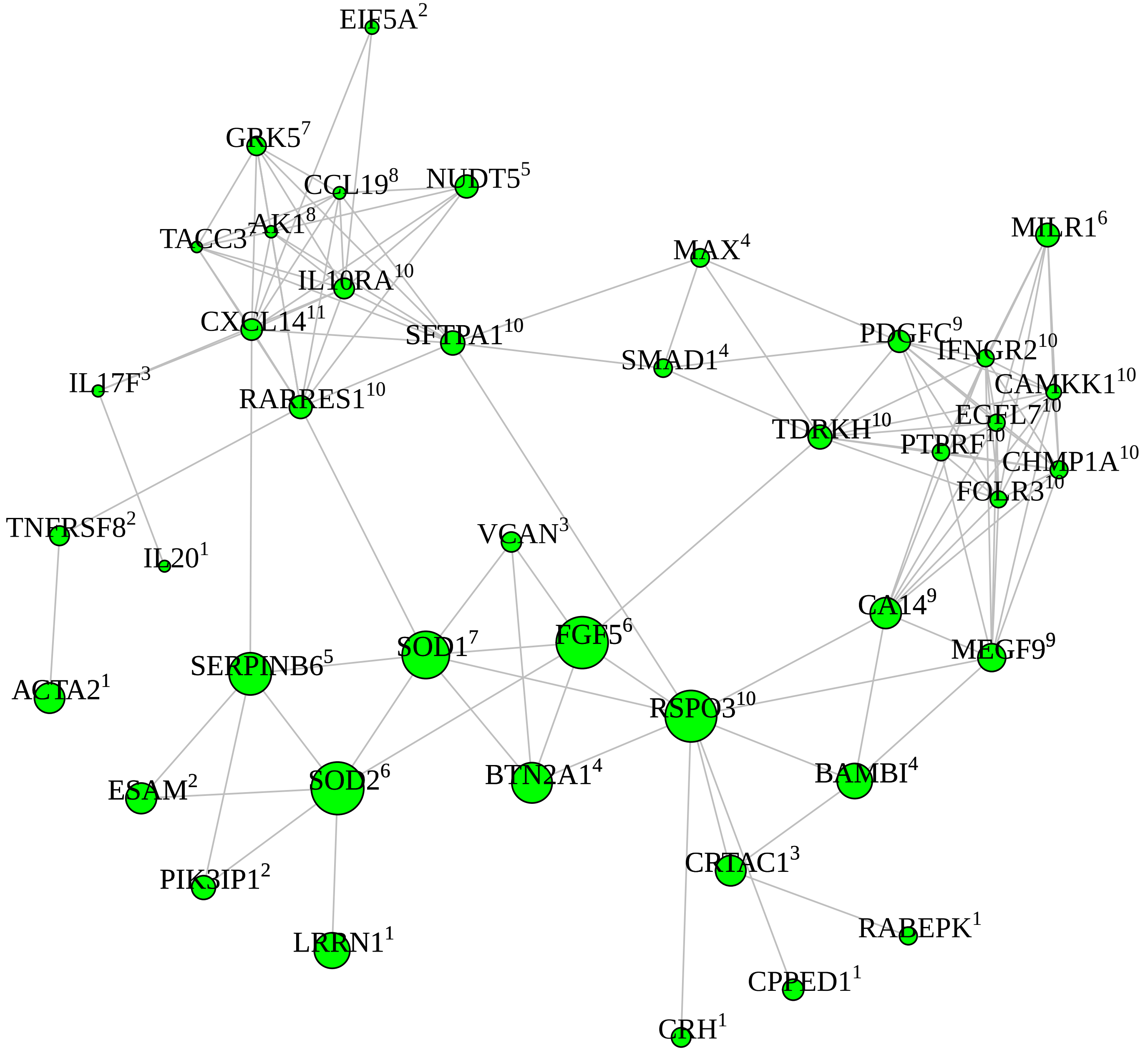

**M1 turquoise module**

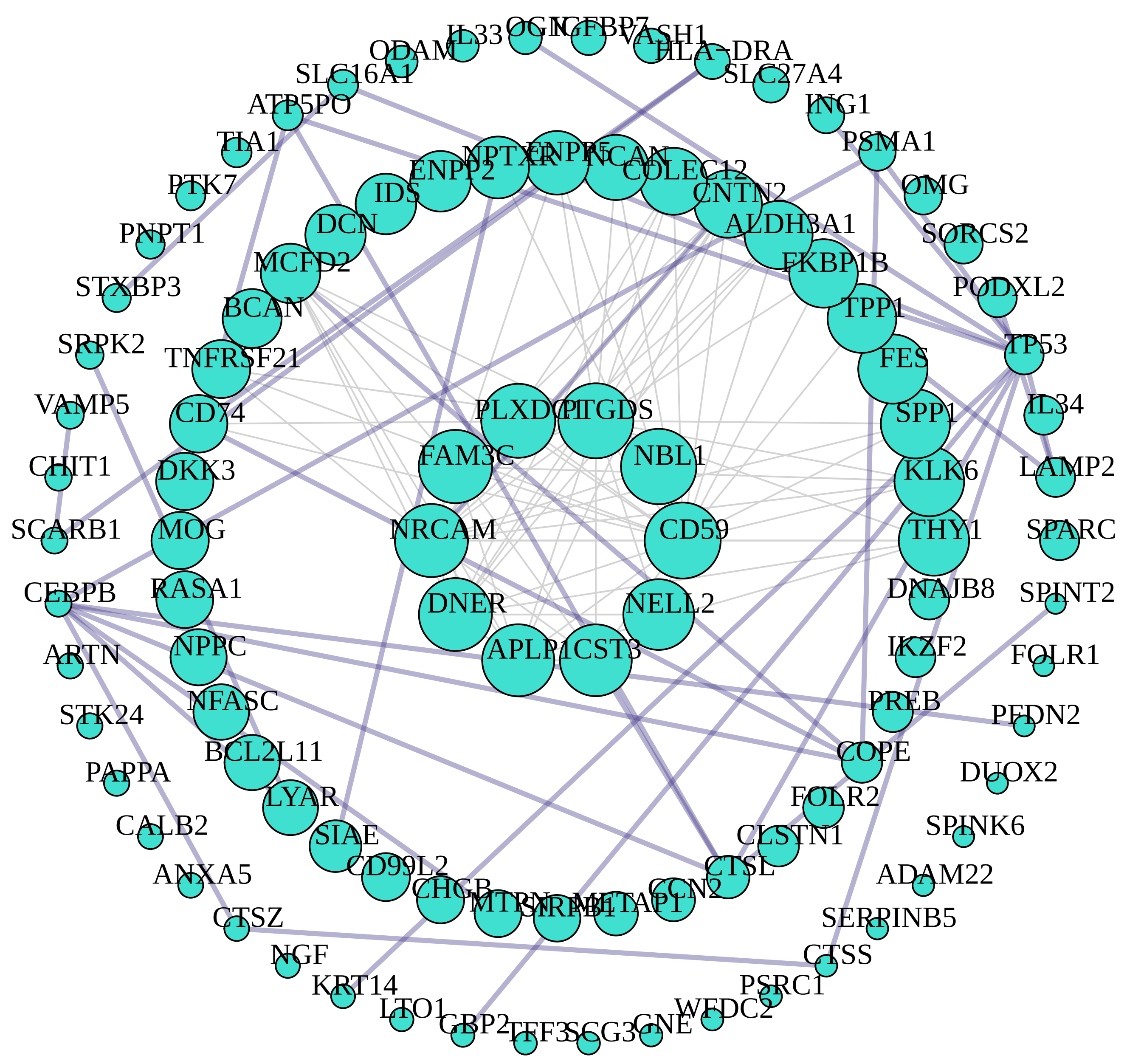


M1 turquoise module hubs connected by top 150 TOM edges: HUB$^{degree}$

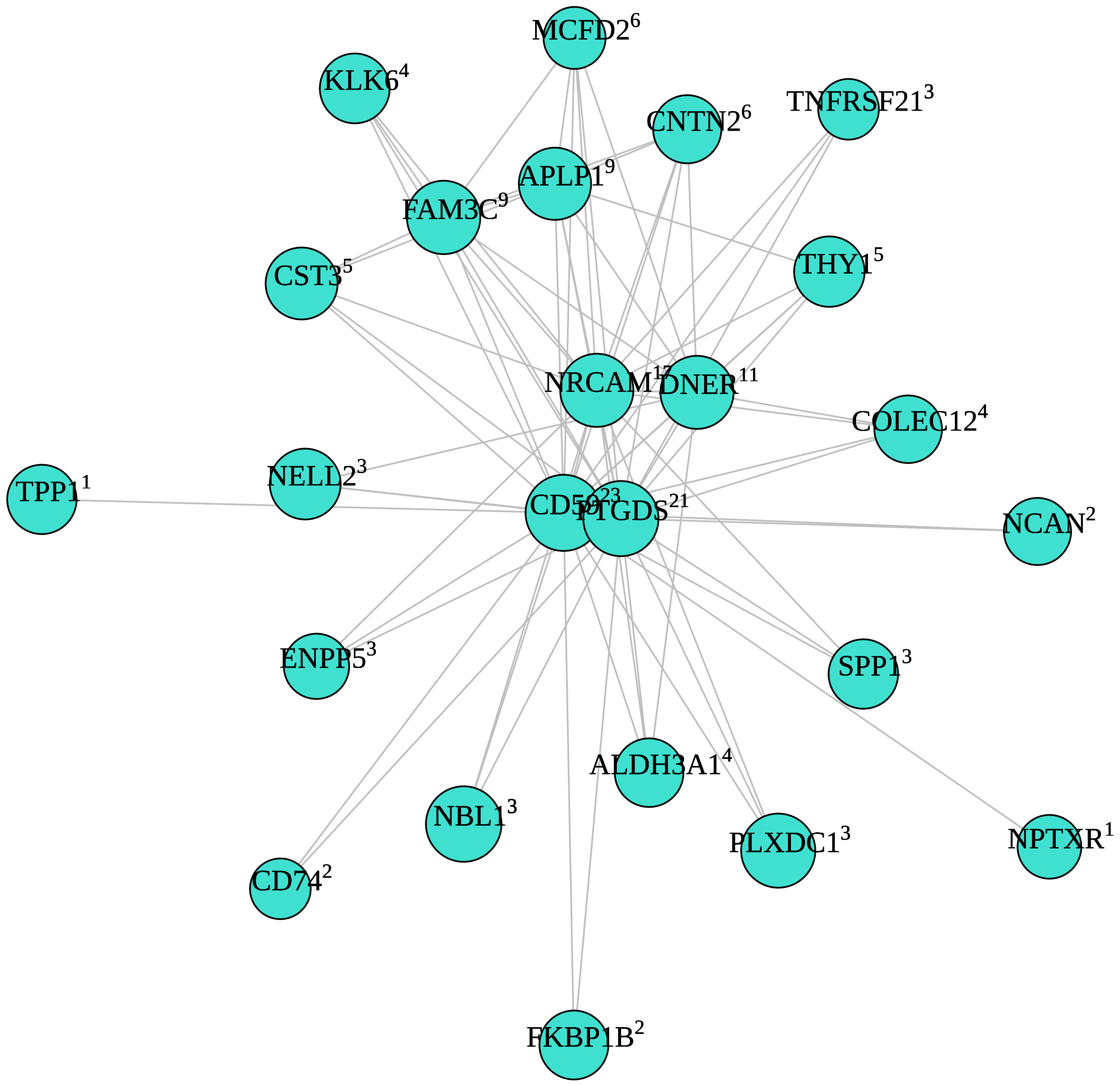

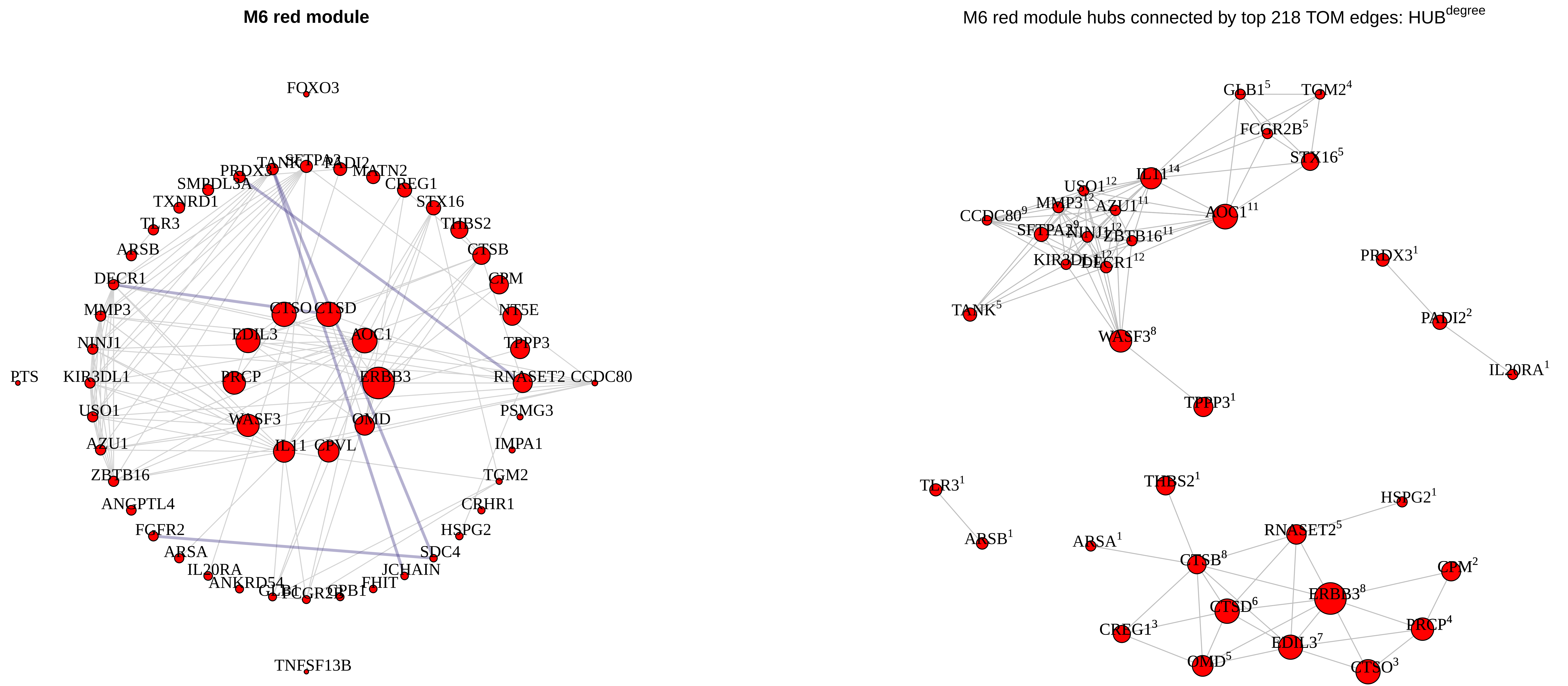
M6 red module
FOXO3
TANK
SFTPA2
PADI2
PRDX3
MATN2
SMPDL3A
CREG1
TXNRD1
STX16
TLR3
THBS2
ARSB
CTSB
DECR1
CPM
MMP3
CTSO
CTSD
NT5E
NINJ1
EDIL3
AOC1
TPPP3
PTS
KIR3DL1
PRCP
ERBB3
RNASET2
CCDC80
USO1
WASF3
OMD
PSMG3
AZU1
IL11
CPVL
IMPA1
ZBTB16
TGM2
ANGPTL4
CRHR1
FGFR2
HSPG2
ARSA
SDC4
IL20RA
JCHAIN
ANKRD54
FHIT
GLB1
FCGR2B
CPB1
TNFSF13B
M6 red module hubs connected by top 218 TOM edges: HUB^degree
GLB1 5
TGM2 4
FCGR2B 5
STX16 5
IL11 14
USO1 12
MMP3 12
AZU1 11
AOC1 11
CCDC80 9
SFTPA2 9
NINJ1 12
ZBTB16 11
KIR3DL1 12
DECR1 12
PRDX3 1
TANK 5
PADI2 2
WASF3 8
IL20RA 1
TPPP3 1
TLR3 1
THBS2 1
HSPG2 1
ARSB 1
ARSA 1
RNASET2 5
CTSB 8
CPM 2
ERBB3 8
CTSD 6
CREG1 3
PRCP 4
EDIL3 7
OMD 5
CTSO 3

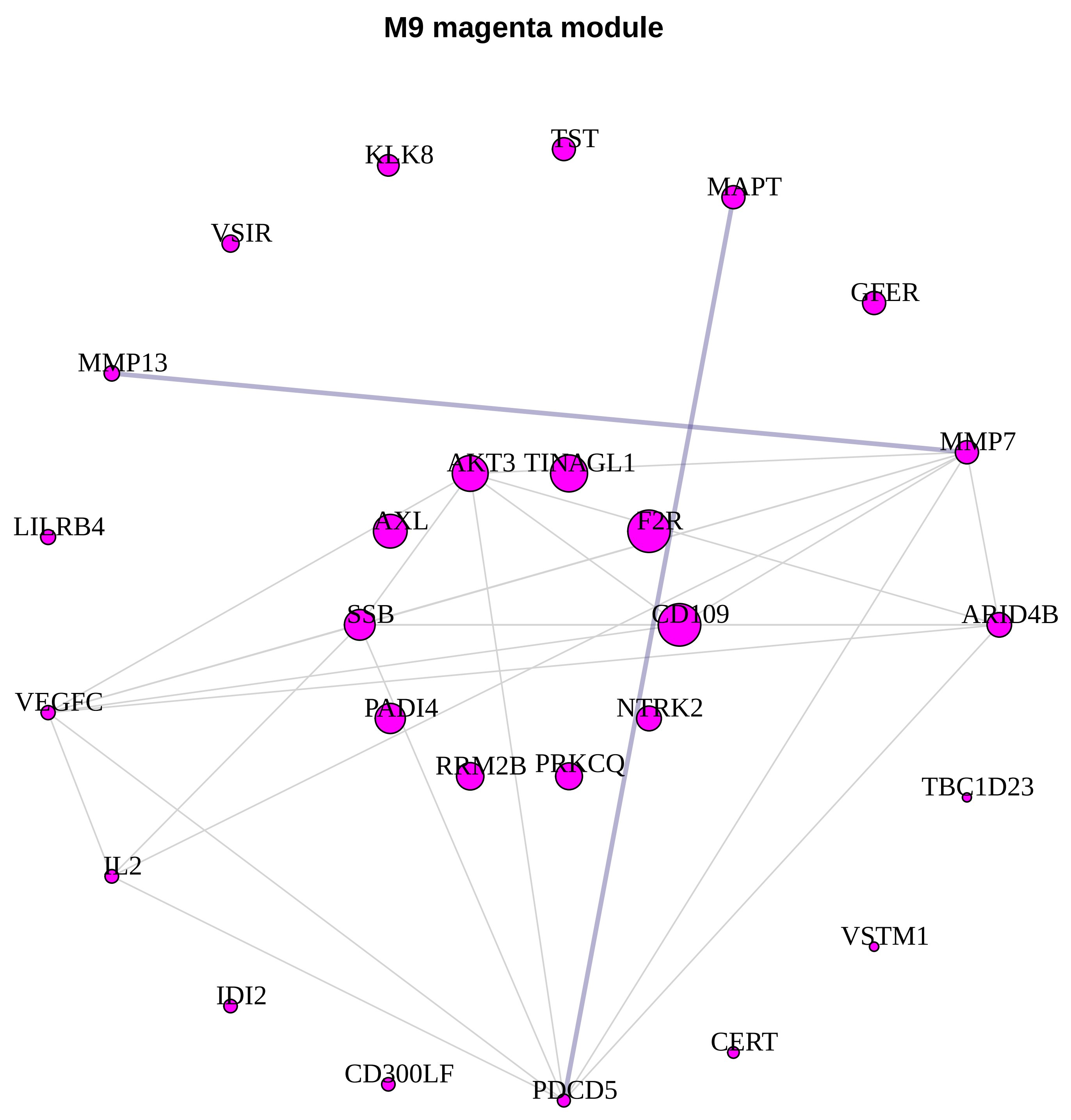
M9 magenta module
KLK8
TST
MAPT
VSIR
GFER
MMP13
MMP7
AKT3
TINAGL1
LILRB4
AXL
F2R
SSB
CD109
ARID4B
VEGFC
PADI4
NTRK2
RRM2B
PRKCQ
TBC1D23
IL2
VSTM1
IDI2
CERT
CD300LF
PDCD5

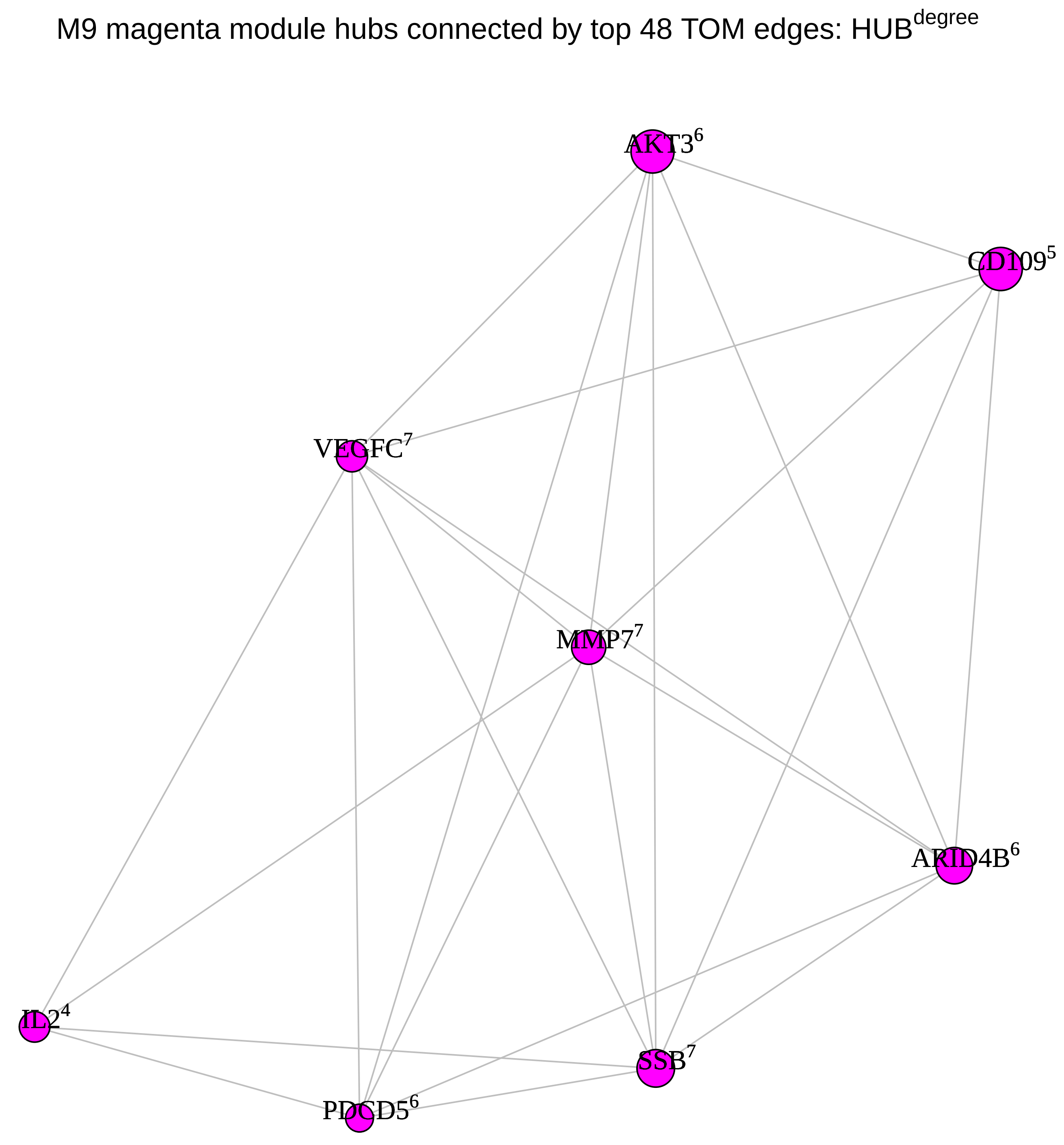
M9 magenta module hubs connected by top 48 TOM edges: HUB$^{degree}$
AKT3$^{6}$
CD109$^{5}$
VEGFC$^{7}$
MMP7$^{7}$
ARID4B$^{6}$
IL2$^{4}$
SSB$^{7}$
PDCD5$^{6}$

**M10 purple module**

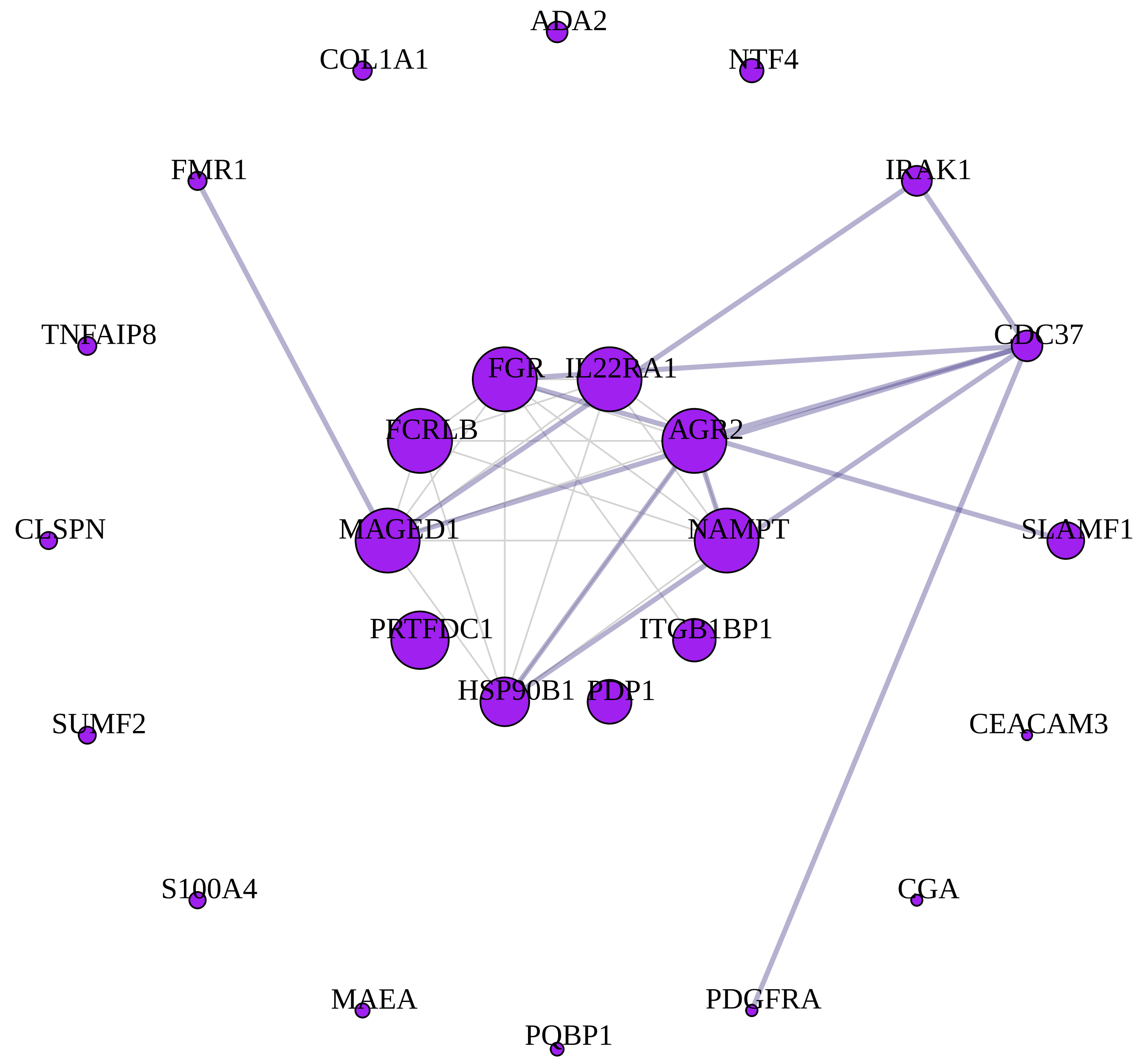


M10 purple module hubs connected by top 44 TOM edges: $HUB^{degree}$

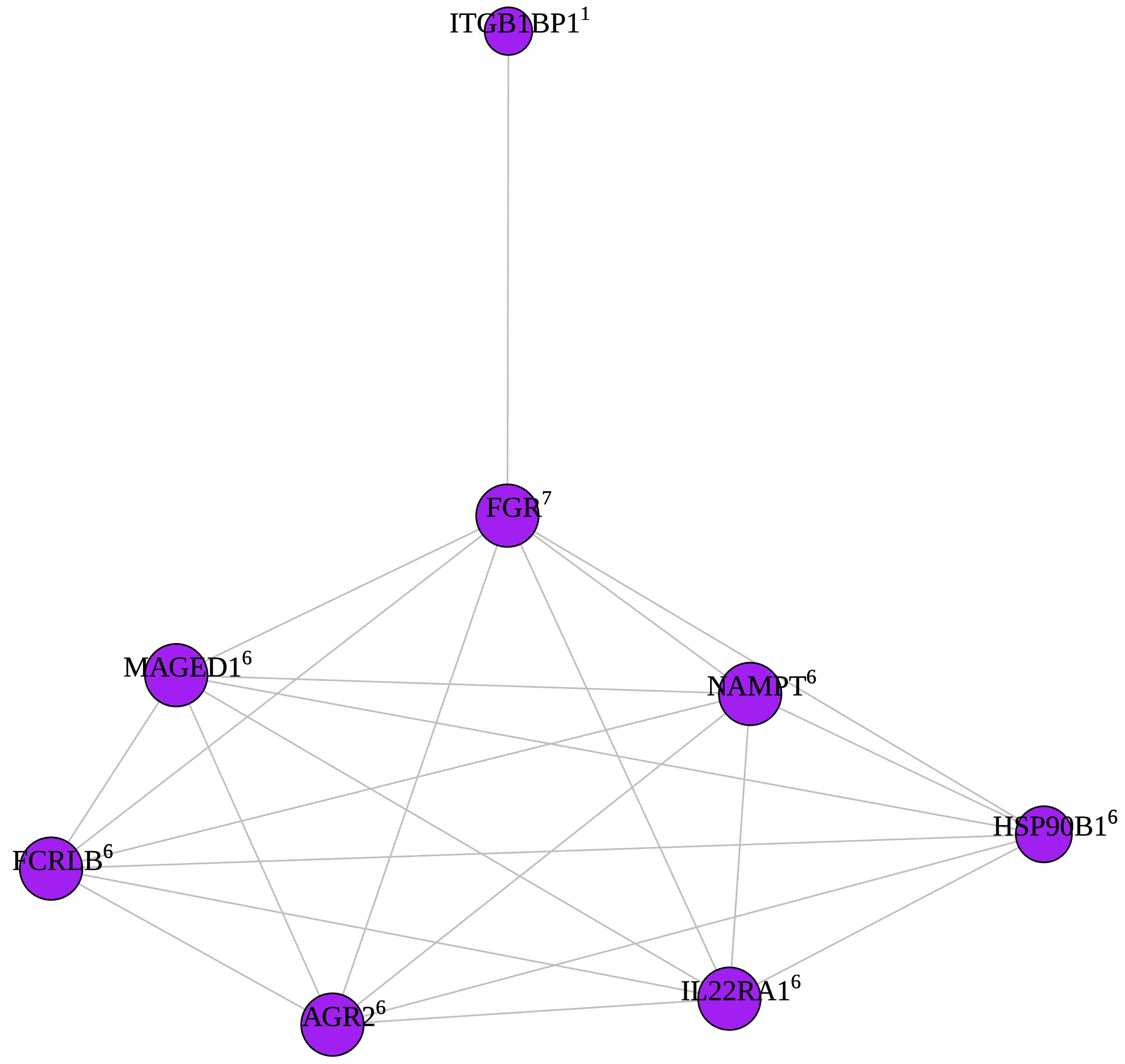

Supplement: Supplement 7 [file media-7.pdf]
