# Supplementary figures and images for: Maintenance of chronic neuroinflammation in multiple sclerosis via interferon signaling and CD8 T cell-mediated cytotoxicity
Source: bioRxiv. 2025 Jun 11:2025.06.09.658729. Preprint. [Version 1] doi: 10.1101/2025.06.09.658729 (PMC12190403; doi:10.1101/2025.06.09.658729)

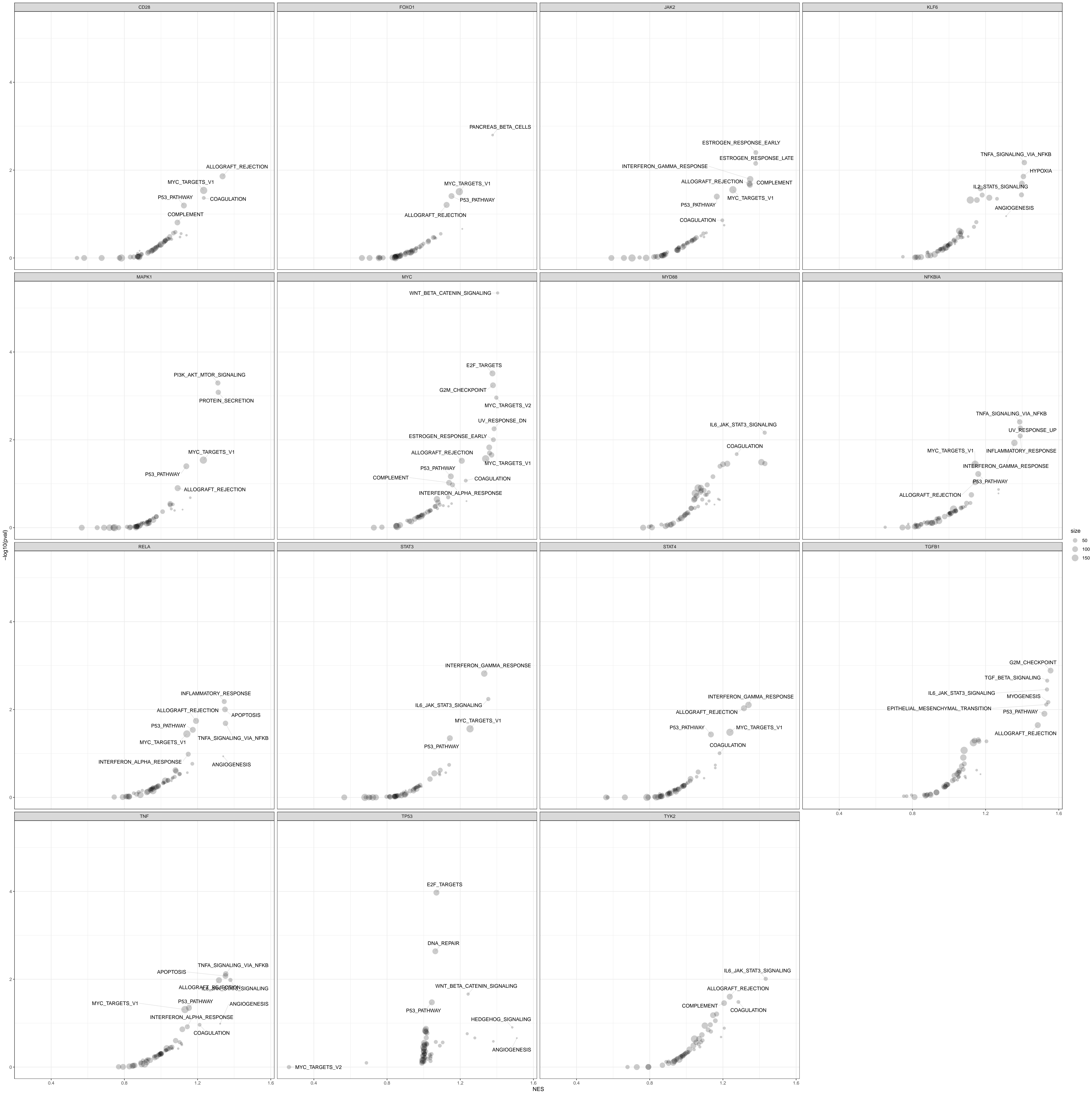

Supplement: Supplement 8 [file media-8.pdf]
